# Shared genetic link and causal inference between blood lipids, lipid-lowering drugs and amyotrophic lateral sclerosis

**DOI:** 10.4103/NRR.NRR-D-25-00266

**Published:** 2025-08-13

**Authors:** Kailin Xia, Ninghao Huang, Yajun Wang, Gan Zhang, Lu Tang, Linjing Zhang, Minhao Yao, Zhonghua Liu, Tao Huang, Dongsheng Fan

**Affiliations:** 1 Department of Neurology, Peking University Third Hospital, Beijing, China; 2 Beijing Key Laboratory of Biomarker and Translational Research in Neurodegenerative Diseases, Beijing, China; 3 Key Laboratory for Neuroscience, National Health Commission/Ministry of Education, Peking University, Beijing, China; 4 Department of Epidemiology and Biostatistics, School of Public Health, Peking University, Beijing, China; 5 Department of Statistics and Actuarial Science, The University of Hong Kong, Hong Kong Special Administrative Region, China

**Keywords:** amyotrophic lateral sclerosis, genetic correlation, genetics, instrumental variables, lipid-lowering drug, lipids, Mendelian randomization, metabolism, nerve regeneration, neurodegenerative disease, risk factor

## Abstract

Growing evidence suggests that abnormal lipid metabolism occurs in amyotrophic lateral sclerosis, even in the presymptomatic stage, implying an etiologic link. However, the genetic mechanism underlying altered lipid levels in amyotrophic lateral sclerosis remains elusive. Therefore, in this study, we performed genetic correlation analysis, a cross-trait meta-analysis, tissue-specific enrichment analysis, and bidirectional two-sample Mendelian randomization analysis of European population to explore whether there is a genetic and causal relationship between lipids and amyotrophic lateral sclerosis. The effect of lipid-lowering drugs on amyotrophic lateral sclerosis was also evaluated using a drug target Mendelian randomization approach. The results showed a positive genetic correlation between amyotrophic lateral sclerosis and both high-density lipoprotein cholesterol and apolipoprotein A1 and identified 71 independent shared loci between amyotrophic lateral sclerosis and high-density lipoprotein cholesterol, as well as 55 independent shared loci between amyotrophic lateral sclerosis and apolipoprotein A1. These shared loci were enriched in the lipid metabolic pathway and the alcohol metabolic pathway. Further Mendelian randomization analysis targeting lipid-lowering drugs showed that single nucleotide polymorphisms within the *ACLY* and *PCSK9* genes had a protective effect against amyotrophic lateral sclerosis risk by decreasing low-density lipoprotein cholesterol. The combination of ACLY and PCSK9 inhibitors has a greater protective effect on amyotrophic lateral sclerosis risk than that of PCSK9 inhibitors alone. In summary, there is a common genetic structure between lipids and amyotrophic lateral sclerosis. Mendelian randomization analysis supports an association between elevated blood lipids and the risk of developing amyotrophic lateral sclerosis, and the use of ACLY or PCSK9 inhibitors may improve disease prognosis.

## Graphical abstract

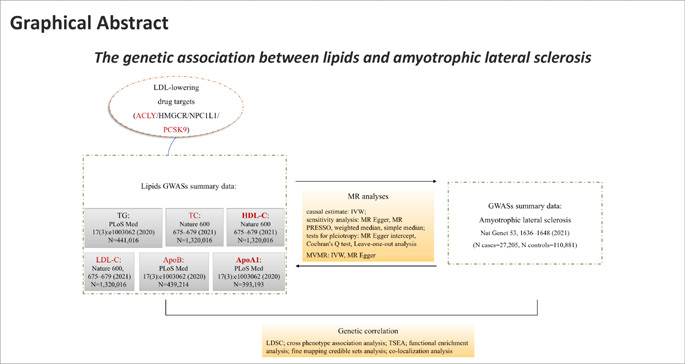

## Introduction

Amyotrophic lateral sclerosis (ALS) is a fatal neurodegenerative disease that involves irreversible loss of upper and lower motor neurons (Masrori and Van Damme, 2020). Patients with ALS die within 3 to 5 years of diagnosis due to respiratory failure (Masrori and Van Damme, 2020). The cause of ALS is largely unknown, making it difficult to develop effective therapeutic interventions. The cumulative lifetime risk of developing ALS is reported to be approximately 1/400 (Brown and Al-Chalabi, 2017). A twin and family study showed that ALS heritability is approximately 50% (Ryan et al., 2019), prompting researchers to explore the interaction between genes and the environment. There is growing evidence of abnormal lipid metabolism in ALS, even in the presymptomatic stage, implying an etiologic link. Some ALS-related genes, including *TDP-43* (Ho et al., 2021), *SOD1* (De Felice et al., 2004), and *CYP27A1* (Björkhem, 2006), help regulate lipid metabolism. Whole-body deletion of *LXR*β, a gene encoding an essential regulator of cholesterol efflux, in male mice produced phenotypes similar to those of ALS (Andersson et al., 2005). These results suggest the possibility of a shared genetic structure between lipid metabolism and ALS. Although classic genome-wide association studies (GWASs) have identified more than 20 ALS-associated genes (Chia et al., 2018), they have failed to identify loci that are significantly shared by a trait of interest and ALS; thus, the genetic mechanism underlying altered lipid metabolism in ALS remains unclear.

Genetic correlation analysis is a novel approach for investigating the correlation of genetic effects and shared genetic variants between two potentially related traits using methodologies such as linkage disequilibrium score regression (LDSC) (Bulik-Sullivan et al., 2015) and multi-trait analysis of GWAS (MTAG) (Turley et al., 2018) and databases such as summary-level statistics from large GWASs and the Human Protein Atlas (HPA) (Thul and Lindskog, 2018). A previous post-GWAS analysis suggested genetic enrichment between ALS and high-density lipoprotein cholesterol (HDL-C) or low-density lipoprotein cholesterol (LDL-C) (Li et al., 2021). However, to our knowledge, no large-scale GWAS has systematically assessed the genetic correlations and shared genetic loci between various lipid fractions and ALS.

In addition, in previous observational studies, evidence for the association between dyslipidemia and ALS has been inconsistent (Seelen et al., 2014; Mariosa et al., 2017; Bjornevik et al., 2021; Thompson et al., 2022; **Additional Table 1**). These inconsistencies may have arisen from differences in the time from baseline measurements to disease onset and from limitations of the methodology (such as residual confounding). The Mendelian randomization (MR) framework is a genetic tool that can be used to estimate the causal association between an exposure and an outcome, using single nucleotide polymorphisms (SNPs) as instrumental variables (IVs). It can provide causal estimates with strong statistical power and inform the prioritization of interventions in a clinical trial (O’Donnell and Sabatine, 2018). Several studies have focused on the association between lipids and ALS, and the conclusions were in general agreement. With much larger sample sizes of recent GWASs and the overlapping IVs among lipid fractions, these results need to be confirmed.

**Additional Table 1:** Summary of previous studies on the values of circulating lipids on ALS as biomarkers

Study	Factors	Setting	Population	Main finding
For ALS incidence observational studies				
Thompson et al. (2022)	TG, TC, TC/HDL-C ratio, HDL-C, LDL-C, ApoAl, ApoB	343 ALS patients vs. 502066 HC	European	Higher HDL-C (HR = 0.84, 95% CI 0.73-0.96, *P* = 0.010) and ApoA1 (HR = 0.83, 95% CI 0.72-0.94, *P* = 0.005) were associated with a reduced risk of ALS. Higher TC/HDL-C was associated with an increased risk of ALS (HR = 1.17, 95% CI 1.05-1.31, *P* = 0.006).
Bjornevik et al. (2021)	.TG, TC, HDL-C, LDL-C	275 ALS patients vs. 20000 HC	European	Higher levels of HDL-C were associated with higher ALS risk (RR Q4 vs.Q1= 1.78, 95% CI 1.18-2.69, *P* = 0.007).
Mariosa et al. (2017)	HDL-C, LDL-C, ApoAl, ApoB	257 ALS patients from a trait consisting of 636132 healthy individuals	European (Sweden)	Increased LDL-C (HR = 1.14; 95%CI 1.02-1.27), ApoB (HR = 1.68; 95% CI 1.17-2.42), ApoB/ApoA1 (HR = 1.90; 95% CI 1.29-2.78) was associated with a higher incidence of ALS.
Seelen et al. (2014)	Hyperchole-sterolemia	722 sporadic ALS patients and 2268 age- and gender-matched controls	European (Netherland)	Hypercholesterolemia was associated with a decreased risk of ALS (OR = 0.76, 95% CI 0.63-0.92, *P* = 0.006).
MR study				
van Rheenen et al. (2021)	TG, TC, HDL-C, LDL-C	N_lipids_ = 188577; N_als_ = 27205 cases & 110881 controls	European	A high level of TC (OR = 1.094, 95%CI 1.032-1.160, *P* = 0.005) increased the risk of ALS, while a high level of LDL-C (OR = 1.073, 95% CI 1.011-1.137, *P* = 0.030) potentially increased the risk of ALS. No evidence of reverse causation.
Zeng and Zhou (2019)	TG, TC, HDL-C, LDL-C	N_lipids_ = 188577; N_als_ = 12577 cases & 23475 controls	European	Higher LDL-C level increases the risk of ALS (OR = 1.14, 95% CI 1.05-1.24, *P* = 1.38E-3).
		N_tg_= 105597; N_tc_= 128305; N_hdl-c_ = 70657; N_ldl-c_= 72866; N_als_ = 1234 cases & 2850 controls	Japanese	Higher LDL-C level increases the risk of ALS (OR = 1.06, 95% CI = 1.00-1.12, *P* = 0.044).
Chen et al. (2018)	TG, TC, HDL-C, LDL-C	N_tg_= 96598; N_tc_= 100184; N_hdl-c_= 99900; N_ldl-c_ = 95454 N_als_ = 12577 cases & 23475 controls	European	High levels of LDL-C (OR= 1.16, 95%CI= 1.10-1.22, *P* = 8.82E-8) and TC (OR= 1.15, 95% CI = 1.09-1.21, *P* = 8.17E-7) are risk factors for ALS.
For ALS prognosis				
Chelstowska et al. (2021)	TG, TC, HDL-C, LDL-C	650 ALS patients	European (Poland)	LDL-C/HDL-C (r = 0.12, *P* = 0.03) and TC (*r* = 0.1003, *P* = 0.02) are associated with longer disease duration
Ingre et al. (2020)	TC, HDL-C, LDL-C, ApoAl, ApoB	99 ALS patients	European (Sweden)	Increased TC (HR = 0.60, 95% CI 0.41-0.89), LDL-C (HR = 0.64, 95% CI 0.44-0.92), LDL-C/HDL-C ratio (HR = 0.65, 95% CI 0.46-0.92), ApoB (HR = 0.62, 95% CI 0.44-0.88), and ApoB/ApoA1 ratio (HR = 0.61, 95% CI 0.43-0.86) are associated with better ALS prognosis.
Liu et al. (2020)	TG, TC, HDL-C, LDL-C	3291 ALS patients and 3367 controls	Mixed population	The levels of all lipids were not associated with mortality of ALS.
Huang et al. (2015)	TG, TC, HDL-C, LDL-C	413 ALS patients and 400 controls	China	Serum lipid levels in ALS patients did not differ significantly from those in healthy controls. ALS patients with higher TG had a longer survival time.
Ikeda et al. (2012)	TG, TC, HDL-C, LDL-C	92 ALS patients	Japanese	TC (*P* < 0.001) and LDL-C (*P* < 0.01) are correlated with clinical deterioration in ALS patients.
Sutedja et al. (2011)	TC, HDL-C, LDL-C	303 ALS patients	European (Netherland)	In the univariate analysis, a higher LDL-C/HDL-C ratio correlated with increased survival (HR = 0.9, *P* = 0.04); after adjusting for the confounders age, site and FVC, no difference was observed.
Dorst et al. (2011)	TG, HDL-C, LDL-C	488 ALS patients	European (Germany)	The prevalence of hypertriglyceridemia (37.9%) is above the common prevalence. High serum levels of both TC (*P* = 0.047) and TG (*P* = 0.006) had a significantly positive effect on survival.
Chio et al. (2009)	TG, TC, HDL-C, LDL-C	658 ALS patients, 658 healthy controls	European (Italy)	The mean levels of TG, TC, HDL-C, LDL-C, and the LDL-C/HDL-C ratio were similar in patients with ALS and controls. LDL-C/HDL-C ratio had no effect on survival. The respiratory impairment, but not a worse clinical status or a lower body mass index, is related to a decrease in blood lipids and LDL-C/HDL-C ratio.
Dupuis et al. (2008)	TG, TC, HDL-C, LDL-C	369 ALS patients, 286 healthy controls	European (France)	Plasma levels of TC and LDL-C were 2-fold higher in patients with ALS. Elevated LDL-C/HDL-C ratio significantly increased survival by more than 12 month.

ALS: Amyotrophic lateral sclerosis; ApoAl: apolipoprotein A1; ApoB: apolipoprotein B; CI: confidence interval; GWAS: genome-wide association study; HDL-C: high-density lipoprotein cholesterol; HR: hazard ratio; LDL-C: low-density lipoprotein cholesterol; MR: Mendelian randomization; OR: odds ratio; RR: relative risk; TC: total cholesterol; TG: total triglycerides.

In this study, we performed genetic correlation analysis, multivariable two-sample MR analysis, and drug target MR analysis of lipid-related traits and ALS using GWAS summary-level data from the latest studies with the largest sample sizes, with the following aims: (i) to improve our understanding of genetic overlap and causality between blood lipids and ALS; (ii) to identify shared loci and potential biological processes contributing to this relationship; and (iii) to explore the potential of regulating lipid levels to decrease ALS risk.

## Methods

### Study design and data collection

The overall study consisted of three parts: (i) to find genetically related trait pairs, we performed large-scale, genome-wide, genetic correlation analysis to calculate the genetic effects of lipids and ALS; (ii) we carried out cross-trait meta-analyses to identify shared loci between each correlated trait pair identified in the first step and to determine whether there were specifically enriched tissues and functional pathways for these loci; and (iii) we conducted bidirectional multivariable two-sample MR analysis to evaluate whether lipids and ALS are causally associated, by drug target MR analysis of lipid-lowering drugs and ALS.

All data analyzed in this study were GWAS summary results from a European population. We used genetic data from a large number of participants for triglycerides (TG, *n* = 441,016) (Richardson et al., 2020), total cholesterol (TC, *n* = 1,320,016) (Graham et al., 2021), HDL-C (*n* = 1,320,016) (Graham et al., 2021), LDL-C (*n* = 1,320,016) (Graham et al., 2021), apolipoprotein A1 (ApoA1, *n* = 393,193) (Richardson et al., 2020), apolipoprotein B (ApoB, *n* = 439,214) (Richardson et al., 2020), and ALS (*n* cases = 27,205; *n* controls = 110,881) (van Rheenen et al., 2021). Patients were diagnosed as having possible, probable, or definite ALS according to the (revised) El Escorial Criteria (van Rheenen et al., 2021). All studies included in our analysis were adjusted for age and sex after quality control, as previously reported (Richardson et al., 2020; Graham et al., 2021; van Rheenen et al., 2021). No patients were directly involved in our study; our study was based on publicly available data only. All human studies included in our analysis were conducted according to the *Declaration of Helsinki*.

### Linkage disequilibrium score regression analysis

We used LDSC to assess the genetic correlation between lipids and ALS, with scores ranging from –1 to 1, assuming that the effect size of a variant is proportional to the number of variants in linkage disequilibrium with it (Bulik-Sullivan et al., 2015). LDSC has high power and can assess the correlation between clinically suggested related traits without the influence of sample overlap. We referred to the 1000 Genomes Project (https://www.internationalgenome.org/1000-genomes-summary/) to obtain a well-established linkage disequilibrium structure of European ancestry. Traits with mean chi-square statistics lower than 1.02 would be likely to generate biased results and insufficient statistical power, and we calculated the mean chi-square statistics of all the traits to ensure that the selected GWAS data met the requirements.

### Cross-trait meta-analysis

First, we conducted large-scale, secondary genome-wide association analysis of lipid GWAS and ALS GWAS summary data to identify significant loci for the two traits using the MTAG approach, which yields lower genome-wide mean-squared error and higher statistical power than single-trait GWAS (Turley et al., 2018). Second, cross-phenotype association (CPASSOC) analysis was performed to identify shared loci between HDL-C and ALS and between ApoA1 and ALS (Li and Zhu, 2017). Considering potential heterogeneity between traits, we calculated scaled heterogeneity (sHet) as the main result. A *P*_sHet_ value less than 5E-8 combined with a *P*-value less than 0.01 for each trait indicated significant shared SNP cross-traits. Independent shared loci (the SNP with the smallest *P*-value and other SNPs at *r*^2^ < 0.001 within 10 MB) were included as shared locus sets. Their gene names are listed, referring to the HUGO Gene Nomenclature Committee (HGNC). We used Harmonizome 3.0 (https://maayanlab.cloud/Harmonizome/gene_set/amyotrophic+lateral+sclerosis/GWASdb+SNP-Disease+Associations) (Diamant et al., 2025) to determine whether these shared genes have previously been reported to be associated with ALS and GeneCards (https://www.genecards.org/) (Stelzer et al., 2016) to determine gene function.

### Fine-mapping credible set analysis

Because a credible variant set can identify causal regions more precisely, we aimed to establish a credible set of causal SNPs and further reveal the underlying molecular mechanisms underlying the association between HDL-C or ApoA1 and ALS. We used independent shared loci as index SNPs, extracted SNPs exhibiting strong linkage near index SNPs (within 500 kb, *r*^2^ > 0.4) and employed a Bayesian likelihood fine-mapping algorithm to identify a 99% credible set of causal loci (Farh et al., 2015).

### Colocalization analysis

We conducted sequential genetic colocalization analysis to determine the potential of an independent shared locus to be a common causal genetic variant. All analyses were performed with the “coloc” R package (University of Cambridge, Cambridge, UK) (Wang et al., 2020). A significant colocalized posterior probability (H4) was defined as a value greater than 0.7.

### Tissue-specific enrichment analysis

We examined whether the shared loci were highly enriched or expressed in a specific tissue. The “TissueEnrich” R package (Iowa State University, Ames, IA, USA) was used to calculate tissue-specific gene enrichment statistics using embedded RNA-Seq data from the HPA program.

### Overexpression analysis

The publicly available online tool WebGestalt (http://www.webgestalt.org/option.php) was used to assess overexpression of these gene sets using Gene Ontology (GO) terms (biological process) (Liao et al., 2019). Benjamini–Hochberg correction was used to control for multiple testing (false discovery rate < 0.05).

### Bidirectional Mendelian randomization analysis

Bidirectional MR analysis provides insight into the causal relationship between lipid levels and ALS and the probable vertical pleiotropy of the shared loci. We included independent, genome-wide associated SNPs (*r*^2^ < 0.001, *P* < 5E-8) from each GWAS as IVs for the corresponding traits. The online tool SNiPA (https://www.snipa.org/snipa3/) (Arnold et al., 2015) was used to search for proxies (*r*^2^ > 0.8) for genetic variants that were unavailable in the outcome dataset.

Inverse-variance weighted (IVW) analysis was the main method used to examine causality (Burgess et al., 2017), involving assessment of the IVW multiplicative random effect and IVW fixed effect. To validate the robustness of the IVW results, we used the simple median method, the weighted median method, and the MR–Egger method as sensitivity analyses, which are more widely applicable than the IVW method, at the cost of reduced statistical power. Focusing on the results that are consistent between IVW and the sensitivity analyses can minimize bias in MR analysis. Heterogeneity and directional pleiotropy were tested using Cochran’s *Q* test (*P* < 0.05) and the Mendelian Randomization Pleiotropy RESidual Sum and Outlier (MR–PRESSO) method (Verbanck et al., 2018); the “MR–PRESSO outlier test” can be used to detect outliers in IVs and provides recalculated results omitting the outliers. The MR–Egger regression intercept was additionally implemented to examine horizontal pleiotropy. A nonsignificant distance from the origin to the intercept indicated the absence of pleiotropy. To determine whether the results were driven by specific SNPs, we conducted a leave-one-out analysis.

**Figure 1:**
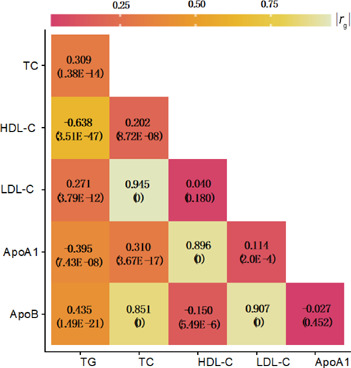
Genetic correlation among lipids, as assessed by LDSC. The size of the *P*-value is indicated by the color, and the number in the grid represents |*r*_g_|. ApoA1: Apolipoprotein A1; ApoB: apolipoprotein B; HDL-C: high-density lipoprotein cholesterol; LDL-C: low-density lipoprotein cholesterol; LDSC: linkage disequilibrium score regression; TC: total cholesterol; TG: total triglycerides.

Considering the close genetic association among lipid fractions, some IVs overlapped in two or more lipid traits, potentially influencing the causal effects of lipids on ALS estimated by univariable MR design. Therefore, we used a multivariable MR (MVMR) framework to diminish the interference caused by overlapping IVs (Burgess and Thompson, 2015). The multivariable IVW approach was adopted as the primary method to examine causality, and the MVMR–Egger method was regarded as the sensitivity analysis. We established two models for the MVMR analyses to avoid collinearity according to genetic correlations among lipids (**Figure 1**). We included all the IVs used for univariable MR analysis of each lipid fraction in this model and clumped them with *r*^2^ = 0.1 to serve as IVs for MVMR. Strict Bonferroni correction was adopted for the MR analyses.

### Drug target Mendelian randomization analysis

The lipid-lowering drugs used in this study included bempedoic acid, statins, ezetimibe, and evolocumab, which primarily target the genes for ATP citrate lyase (*ACLY*), 3-hydroxy-3-methylglutaryl coenzyme A reductase (*HMGCR*), Niemann–Pick C1-like 1 (*NPC1L1*), and proprotein convertase subtilisin/kexin type 9 (*PCSK9*), respectively. To mimic their lipid-lowering effects, we set the criteria as follows: i) genetic variants from LDL-C GWAS within a drug target gene window (within 100 kb of the target gene for each drug); (ii) *P*-values in LDL-C GWAS less than 5E-8; (iii) minor allele frequencies greater than 0.01; and (iv) IVs in relatively weak linkage disequilibrium (*r*^2^ < 0.40 within 100 kb). The effect allele was defined as the allele associated with a reduced LDL-C level. Because it was not defined whether the protective effect of gene-driven lowered LDL-C levels on ALS was indirect (dependent on lipids), we reconducted MR analyses using all IVs outside the target gene region for LDL-C as a sensitivity analysis. If both the MR estimates based on IVs located in drug target genes and IVs in the rest of the genome were significant, the causal effect of the drugs on ALS was classified as indirect.

## Results

### Genome-wide genetic correlations in amyotrophic lateral sclerosis

We estimated SNP-based heritability on the observed scale with the GWAS summary-level genetic statistics included in our study (**Table 1**). Using the LDSC method, we found a positive genetic correlation between ALS and both HDL-C (*r*_g_ = 0.068, *P* = 0.027) and ApoA1 (*r*_g_ = 0.091, *P* = 0.013) (**Table 1**). However, we did not find a similar correlation between ALS and TG, TC, LDL-C, or ApoB (all *P* > 0.05).

**Table 1:** The features of the GWAS datasets used in the study

Trait	Sample size	Heritability			Genetic correlation with ALS		
		
		Mean chi-square	h^2^		r_g_	se	*P*-value
TG	441016	2.795	0.166		–0.051	0.035	0.156
TC	1320016	3.533	0.087		0.043	0.034	0.199
HDL-C	1320016	4.031	0.103		0.068	0.031	0.027
LDL-C	1320016	3.08	0.074		0.031	0.037	0.404
ApoA1	393193	2.719	0.171		0.091	0.036	0.013
ApoB	439214	2.017	0.087		0.014	0.048	0.78
ALS	138086	1.131	0.038				

ALS: Amyotrophic lateral sclerosis; ApoA1: apolipoprotein A1; ApoB: apolipoprotein B; GWAS: genome-wide association study; HDL-C: high-density lipoprotein cholesterol; LDL-C: low-density lipoprotein cholesterol; TC: total cholesterol; TG: total triglycerides.

### Cross-trait meta-analysis of high-density lipoprotein cholesterol- or apolipoprotein A1-related traits and amyotrophic lateral sclerosis

**Figure 2:**
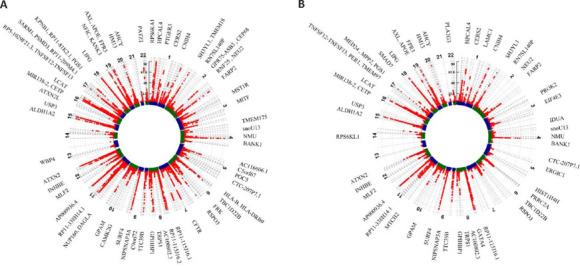
Cross-trait meta-analysis circus Manhattan plots of HDL-C- and ApoA1-related traits and ALS. (A) HDL-C and ALS. (B) ApoA1 and ALS. The outer layer illustrates independent shared genes between the two traits, the intermediate layer marks the chromosome position, and the inner layer indicates the significance level –log10 (*P* value) shared markers from cross-trait meta-analysis (e.g., the numbers 22.5, 45, 67.5, and 90 represents the –log10 (*P* value) of SNPs). Red dots indicate genome-wide significance (*P* < 5E–8). *P*-values out of range (1E–90) are treated as the maximum of the range. Genes located close together are assigned the same gene label separated by commas. Blue and green are used to distinguish adjacent chromosomes. ALS: Amyotrophic lateral sclerosis; ApoA1: apolipoprotein A1; HDL-C: high-density lipoprotein cholesterol.

First, we performed MTAG analysis to generate lower genome-wide mean-squared error and higher statistical power for the corresponding single-trait GWAS. Given the observed close genetic links between HDL-C or ApoA1 and ALS, we conducted CPASSOC analysis to identify significant shared genetic loci influencing the overall correlation of the meta-analysis (*P*_sHet_ < 5E-8 and single trait *P*_MTAG_ < 0.01, *r*^2^ = 0.001 within 10 MB). Manhattan plots were generated to show the results, with the shared genetic loci marked (**Figure 2**). We identified 71 independent shared loci for HDL-C and ALS with genome-wide significance (**Additional Table 2**). Fifty-six shared loci were classified as protein-coding genes, 52 of which have not been reported in previous ALS GWASs. The strongest signal mapped to chromosome 16 in *CETP* (index SNP rs72771478, *P*_sHet_ = 1.52E-153), the gene encoding cholesteryl ester transfer protein. The second strongest signal was a missense variant in *APOE* (index SNP rs7412, *P*_sHet_ = 2.84E-150), a gene closely related to lipoprotein glomerulopathy and hyperlipoproteinemia. We found 55 significant shared loci between ApoA1 and ALS through the CPASSOC approach (**Additional Table 3**). Forty-seven shared loci were protein-coding genes, of which 45 loci have not been mentioned in published ALS GWASs. The locus with the most significant *P*_sHet_ was also located in *APOE* (index SNP rs7412, *P*_sHet_ = 1.44E-177), showing genetic overlap between HDL-C-related traits and ALS.

**Additional Table 2:** Characteristics of the shared loci between ALS and HDL-C

SNP	hg19_coordinates	a1	A1 frequency	*P*	Consequence	HGNC	Gene function
rs7726	chr1:26901319	A	0.2584	2.13E-10	3’UTR	*RPS6KA1*	Protein coding gene
rs72665235	chr1:40157643	C	0.9254	1.26E-17	Upstream	*HPCAL4*	Protein coding gene
rs650194	chr1:71414863	A	0.333	6.83E-12	Intron	*PTGER3*	Protein coding gene
rs267738	chr1:150940625	T	0.8091	5.36E-32	Missense	*CERS2*	Protein coding gene
rs56105022	chr1:224544746	A	0.0338	1.32E-11	Intron	*CNIH4*	Protein coding gene
rs7595075	chr2:264019	A	0.334	1E-21	5’UTR	*SH3YL1*	Protein coding gene
rs66906321	chr2:630070	C	0.8201	4E-10	Intergenic	*TMEM18*	Protein coding gene
rs1107850	chr2:20371772	G	0.5517	3.36E-17	Upstream	*RN7SL140P*	Pseudogene
rs6724214	chr2:54034881	G	0.8787	3.95E-09	Intron	*GPR75-ASB3*	Protein coding gene
rs2540948	chr2:65284623	T	0.6421	7.95E-20	Intron	*CEP68*	Protein coding gene
rs2303564	chr2:219529881	A	0.0219	2.11E-10	Synonymous	*RNF25*	Protein coding gene
rs1515114	chr2:227098387	A	0.5239	1.01E-65	Intergenic	*NEU2*	Protein coding gene
rs4675812	chr2:242395674	G	0.3857	7.34E-17	Intron	*FARP2*	Protein coding gene
rs111873781	chr3:49930316	T	0.0954	3.32E-08	Intron	*MST1R*	Protein coding gene
rs4855447	chr3:69803328	A	0.2237	6.33E-09	Intron	*MITF*	Protein coding gene
rs34311866	chr4:951947	T	0.8131	2.14E-17	Missense	*TMEM175*	Protein coding gene
rs56122753	chr4:26031073	A	0.1779	8.66E-23	Intergenic	*snoU13*	Pseudogene
rs7684939	chr4:55509189	A	0.4712	1.14E-10	Intergenic	*NMU*	Protein coding gene
rs114614648	chr4:102810526	C	0.6958	1.2E-15	Intron	***BANK1***	Protein coding gene
rs7736891	chr5:52808873	A	0.5686	3.27E-08	Intergenic	*AC1166061*	No results
rs74557927	chr5:55825745	A	0.0278	2.73E-09	Intron	*C5orf67*	RNA gene
rs2112347	chr5:75015242	G	0.3678	8.17E-21	Upstream	*POC5*	Protein coding gene
rs2434612	chr5:158022041	A	0.7903	5.5E-27	Intergenic	*CTC-207P7.1*	No results
rs62396278	chr6:31329119	C	0.0626	1.89E-20	Upstream	*HLA-B*	Protein coding gene
rs9268839	chr6:32428772	A	0.5487	2.53E-11	Intron	*HLA-DRB9*	Pseudogene
rs3800406	chr6:35133074	G	0.1024	3.17E-33	Intergenic	*TBC1D22B*	Protein coding gene
rs3798241	chr6:116292883	G	0.2803	3.06E-13	Intron	*FRK*	Protein coding gene
rs2186037	chr6:127455029	A	0.5149	1.56E-33	Intron	*RSPO3*	Protein coding gene
rs3808184	chr7:117214125	T	0.661	2.87E-09	Intron	***CFTR***	Protein coding gene
rs17717355	chr8:9198695	G	0.9294	3.2E-20	Intron	*RPU-U5J16.1*	No results
rs77705851	chr8:9293292	C	0.9453	8.74E-09	Intron	*RP11-115J16.2*	No results
rs77913157	chr8:19949198	G	0.9483	3.37E-30	Intron	*AC100802.3*	No results
rs3808461	chr8:116597716	C	0.4195	5E-94	Intron	*TRPS1*	Protein coding gene
rs6420185	chr8:144303071	A	0.4056	1.28E-33	Downstream	*GPIHBP1*	Protein coding gene
rs686030	chr9:15304782	A	0.8698	6.7E-109	Intron	*TTC39B*	Protein coding gene
rs2492816	chr9:27565105	A	0.4145	8.16E-18	Intron	***C9orf72***	Protein coding gene
rs117323391	chr9:107511975	C	0.9732	2.77E-15	Intron	*NIPSNAP3A*	Protein coding gene
rs56343119	chr9:136237672	A	0.1491	2.15E-12	Intron	*SURF4*	Protein coding gene
rs7098444	chr10:75580042	C	0.3787	9.13E-09	Intron	*CAMK2G*	Protein coding gene
rs2792751	chr10:113940329	C	0.6918	1.65E-70	Missense	*GPAM*	Protein coding gene
rs11039389	chr11:47796062	C	0.3588	2.2E-85	Downstream	*NUP160*	Protein coding gene
rs117186302	chr11:61456420	C	0.0199	3.22E-08	Intron	*DAGLA*	Protein coding gene
rs12575636	chr11:95311260	G	0.2117	1.56E-09	Intergenic	*RP11-338H14.1*	No results
rs4938340	chr11:116980622	A	0.1352	7.58E-66	Downstream	*AP000936.4*	No results
rs11064373	chr12:6871709	A	0.2227	1.1E-10	Intron	*MLF2*	Protein coding gene
rs3809114	chr12:57848639	G	0.4672	6.52E-37	5’UTR	*INHBE*	Protein coding gene
rs10774625	chr12:111910219	A	0.4771	6.59E-40	Intron	*ATXN2*	Protein coding gene
rs17532413	chr13:41644956	A	0.9563	4.65E-12	Intron	*WBP4*	Protein coding gene
rs113629348	chr15:58545668	C	0.9732	1.33E-22	Intron	*ALDH1A2*	Protein coding gene
rs2058914	chr15:63831984	A	0.6879	1.84E-11	Intron	*USP3*	Protein coding gene
rs62036658	chr16:28850371	G	0.338	9.51E-10	Downstream	*ATXN2L*	Protein coding gene
rs117254618	chr16:56892818	A	0.9851	1.33E-40	Downstream	*MIR138-2*	RNA gene
rs72771478	chr16:57002118	G	0.9881	1.52E-153	Intron	*CETP*	Protein coding gene
rs1109166	chr16:67977382	T	0.8151	1.61E-142	5’UTR	*LCAT*	Protein coding gene
rs16952930	chr17:401847	C	0.1352	9.89E-12	Upstream	*RP5-1029F21.3*	No results
rs3803800	chr17:7462969	A	0.2326	3.21E-24	Missense	*TNFSF12-TNFSF13*	Protein coding gene
rs10853128	chr17:26721304	A	0.4841	1.52E-09	Intron	***SARMI***	Protein coding gene
rs12453334	chr17:38153473	C	0.6421	1.99E-28	Intron	*PSMD3*	Protein coding gene
rs7213935	chr17:41801263	A	0.3489	1.32E-13	Upstream	*RP11-209M4.1*	No results
rs8078686	chr17:45735706	C	0.5129	1.09E-17	Intron	*KPNB1*	Protein coding gene
rs28412876	chr17:47454515	G	0.6243	3.93E-08	Intron	*RP11-81K21*	No results
rs4969182	chr17:76393030	C	0.5099	1.94E-80	Intron	*PGS1*	Protein coding gene
rs3819166	chr18:47102102	A	0.1849	5.71E-46	Intron	*LIPG*	Protein coding gene
rs7254731	chr19:3405692	A	0.8996	1.1E-12	Intron	*NFIC*	Protein coding gene
rs35390907	chr19:8393592	A	0.1243	6.58E-14	Intron	*KANK3*	Protein coding gene
rs73045226	chr19:41760913	A	0.8966	5.56E-11	Intron	*AXL*	Protein coding gene
rs7412	chr19:45412079	C	0.9374	2.84E-150	Missense	*APOE*	Protein coding gene
rs28850399	chr19:52314227	C	0.1103	7.16E-21	Intron	*FPR3*	Protein coding gene
rs6058035	chr20:30120782	A	0.1571	1.57E-11	Intron	*HM13*	Protein coding gene
rs7268701	chr20:32901960	C	0.499	4.82E-16	Upstream	*AHCY*	Protein coding gene
rs9609261	chr22:31743032	A	0.9423	1.32E-08	Upstream	*PATZ1*	Protein coding gene

*P* value in cross-trait GWAS analysis. Bold, gene reported to be associated with ALS before, by consulting an online tool Harmonizome 3.0 (https://maavanlab.doud/Harmonizome/aene_set/amyotrophic+lateral+sclerosis/GWASdb+SNP-Disease+Associations). No results, not found in GeneCards (https://www.aenecards.org/). ALS: amyotrophic lateral sclerosis; HDL-C: high-density lipoprotein cholesterol; HGNC: HUGO Gene Nomenclature Committee; SNP: single nucleotide polymorphism; UTR: untranslated region.

**Additional Table 3:** Characteristics of the shared loci between ALS and ApoA1

SNP	hg19_coordinates	a1	A1 frequency	*P*	Consequence	HGNC	Gene function
rs72665235	chr1:40157643	C	0.9254	1.75E-11	Upstream	*HPCAL4*	Protein coding gene
rs267738	chr1:150940625	T	0.8091	6.59E-40	Missense	*CERS2*	Protein coding gene
rs2877984	chr1:183090497	G	0.5636	1.87E-09	Intron	*LAMC1*	Protein coding gene
rs56105022	chr1:224544746	A	0.0338	4.48E-11	Intron	*CNIH4*	Protein coding gene
rs7595075	chr2:264019	A	0.334	5.52E-09	5’UTR	*SH3YL1*	Protein coding gene
rs1107850	chr2:20371772	G	0.5517	1.32E-26	Upstream	*RN7SL140P*	Pseudogene
rs1515114	chr2:227098387	A	0.5239	2.38E-30	Intergenic	*NEU2*	Protein coding gene
rs4675812	chr2:242395674	G	0.3857	7.42E-11	Intron	*FARP2*	Protein coding gene
rs9837992	chr3:70959438	A	0.3231	4.09E-08	Intergenic	*PROK2*	Protein coding gene
rs73090625	chr3:71780696	A	0.0865	9.72E-09	Intron	*EIF4E3*	Protein coding gene
rs11248061	chr4:980896	A	0.4274	2.09E-10	Missense	*IDUA*	Protein coding gene
rs1022186	chr4:26037388	T	0.1779	6.61E-12	Intergenic	*snoU13*	Pseudogene
rs11133338	chr4:55513275	C	0.2972	1.38E-08	Intergenic	*NMU*	Protein coding gene
rs34166099	chr4:102778580	A	0.3062	6.32E-13	Intron	*BANK1*	Protein coding gene
rs2434612	chr5:158022041	A	0.7903	2.58E-11	Intergenic	*CTC-207P7.1*	No results
rs2446192	chr5:172352369	T	0.6133	3.77E-08	Intron	*ERGIC1*	Protein coding gene
rs4412192	chr6:26290377	A	0.5417	3.57E-08	Upstream	*HIST1H4H*	Protein coding gene
rs3130070	chr6:31591808	A	0.8539	2.81E-22	Intron	*PRRC2A*	Protein coding gene
rs11755266	chr6:35162141	C	0.9155	4.07E-20	Intergenic	*TBC1D22B*	Protein coding gene
rs2489623	chr6:127455821	A	0.505	2.34E-11	Intron	*RSPO3*	Protein coding gene
rs17717355	chr8:9198695	G	0.9294	5.31E-11	Intron	*RP11-115J16.1*	No results
rs17153747	chr8:11611353	C	0.1083	4.62E-10	Intron	*GATA4*	Protein coding gene
rs77913157	chr8:19949198	G	0.9483	9E-11	Intron	*AC100802.3*	No results
rs10505258	chr8:116602855	C	0.5855	6.24E-56	Intron	*TRPS1*	Protein coding gene
rs6420185	chr8:144303071	A	0.4056	4.82E-34	Downstream	*GPIHBP1*	Protein coding gene
rs686030	chr9:15304782	A	0.8698	2.72E-70	Intron	*TTC39B*	Protein coding gene
rs117323391	chr9:107511975	C	0.9732	3.81E-10	Intron	*NIPSNAP3A*	Protein coding gene
rs56343119	chr9:136237672	A	0.1491	7.9E-18	Intron	*SURF4*	Protein coding gene
rs2792751	chr10:113940329	C	0.6918	7.01E-52	Missense	*GPAM*	Protein coding gene
rs11039324	chr11:47665686	A	0.4225	1.32E-43	Upstream	*MTCH2*	Protein coding gene
rs11021232	chr11:95320808	C	0.1978	1.34E-09	Intergenic	*RP11-338H14.1*	No results
rs4938340	chr11:116980622	A	0.1352	1.48E-140	Downstream	*AP000936.4*	No results
rs67385163	chr12:6864890	A	0.662	8.88E-10	Intron	*MLF2*	Protein coding gene
rs3809114	chr12:57848639	G	0.4672	4.33E-25	5’ utr	*INHBE*	Protein coding gene
rs10774625	chr12:111910219	A	0.4771	2E-13	Intron	*ATXN2*	Protein coding gene
rs4899539	chr14:75384162	C	0.5527	4.92E-08	Intron	*RPS6KL1*	Protein coding gene
rs113629348	chr15:58545668	C	0.9732	3.59E-27	Intron	*ALDH1A2*	Protein coding gene
rs2058914	chr15:63831984	A	0.6879	1.67E-08	Intron	*USP3*	Protein coding gene
rs117254618	chr16:56892818	A	0.9851	1.96E-15	Downstream	*MIR138-2*	RNA gene
rs72771478	chr16:57002118	G	0.9881	1.51E-37	Intron	*CETP*	Protein coding gene
rs1109166	chr16:67977382	T	0.8151	9.93E-49	5’ utr	*LCAT*	Protein coding gene
rs78608249	chr17:7455707	C	0.7256	2.48E-11	Intron	*TNFSF12-TNFSF13*	Protein coding gene
rs2518026	chr17:8052363	T	0.3827	1.99E-10	Intron	*PER1*	Protein coding gene
rs241776	chr17:26644308	T	0.4264	4.56E-23	Upstream	*TMEM97*	Protein coding gene
rs2302774	chr17:38183090	T	0.3748	1.93E-11	Intron	*MED24*	Protein coding gene
rs231517	chr17:41961789	T	0.832	5.74E-10	Intron	*MPP2*	Protein coding gene
rs17561950	chr17:76392144	A	0.4841	6.82E-41	Intron	*PGS1*	Protein coding gene
rs4464148	chr18:46459032	C	0.3419	6.64E-10	Intron	*SMAD7*	Protein coding gene
rs7237346	chr18:47058417	A	0.1988	1.2E-40	Intergenic	*LIPG*	Protein coding gene
rs144025261	chr19:41756563	G	0.8966	1.06E-09	Intron	*AXL*	Protein coding gene
rs7412	chr19:45412079	C	0.9374	1.44E-177	Missense	*APOE*	Protein coding gene
rs28850399	chr19:52314227	C	0.1103	2.95E-08	Intron	*FPR3*	Protein coding gene
rs6059958	chr20:30143278	C	0.832	6.33E-10	Intron	*HM13*	Protein coding gene
rs7268701	chr20:32901960	C	0.499	7.88E-09	Upstream	*AHCY*	Protein coding gene
rs2232170	chr22:31537122	G	0.7883	3.95E-08	Upstream	*PLA2G3*	Protein coding gene

*P* value in cross-trait GWAS analysis. Bold, gene reported to be associated with ALS before, by consulting an online tool Harmonizome 3.0 (https://maavanlab.doud/Harmomzome/aene_set/amyotrophic+lateral+sclerosis/GWASdb+SNP-Disease+Associations). No results, not found in GeneCards (https://www.aenecards.ora/). ALS: Amyotrophic lateral sclerosis; ApoAl: apolipoprotein A1; HGNC: HUGO Gene Nomenclature Committee; SNP: single nucleotide polymorphism; UTR: untranslated region.

### Tissue-specific enrichment analysis of high-density lipoprotein cholesterol-related traits and amyotrophic lateral sclerosis

**Figure 3:**
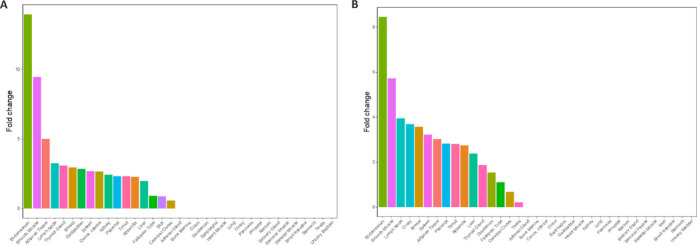
Tissue enrichment analysis of the expression of cross-trait-associated genes for ALS and HDL-C and ALS and ApoA1. (A) Enriched tissues for loci shared between HDL-C and ALS. (B) Enriched tissues for loci shared between ApoA1 and ALS. Fold change refers to the ratio of the expression level of the target genes in a specific tissue to the average expression level of all the genes detected in that tissue. ALS: Amyotrophic lateral sclerosis; ApoA1: apolipoprotein A1; HDL-C: high-density lipoprotein cholesterol.

To evaluate whether the shared genes between HDL-C–related traits and ALS were enriched for expression in specific tissues, we performed tissue-specific enrichment analysis using the HPA platform. There were 17 significantly enriched tissues for loci shared between HDL-C and ALS, including endometrium, smooth muscle, adipose tissue, lymph node, thyroid gland, breast, gallbladder, spleen, cervix and uterus, kidney, placenta, tonsil, appendix, liver, fallopian tube, skin, and cerebral cortex (**Figure 3A**). The genes shared between ApoA1 and ALS were specifically enriched in 16 tissues, including endometrium, smooth muscle, lymph node, ovary, breast, spleen, adipose tissue, placenta, tonsil, appendix, liver, thyroid gland, duodenum, fallopian tube, cerebral cortex, and testis (**Figure 3B**). In summary, tissues enriched for both HDL-C or ApoAl and ALS were mainly those belonging to the reproductive system and the immune system.

### Overexpression analysis of the high-density lipoprotein cholesterol or apolipoprotein A1 and amyotrophic lateral sclerosis shared loci sets

To investigate the functional mechanisms of the shared loci, we used GO terms to identify potential shared biological processes between ALS and HDL-C or ApoA1. The top 10 significant processes enriched in the HDL-C and ALS shared loci set were generally associated with the lipid metabolic pathway and the alcohol metabolic pathway, such as the acylglycerol metabolic process, neutral lipid metabolic process, cholesterol metabolic process, and secondary alcohol metabolic process (**Table 2**). However, we did not observe any enriched processes in the ApoA1 and ALS shared loci set (**Table 2**).

**Table 2:** Biological processes of shared gene sets between ALS and HDL-C or ApoA1

Trait	Gene set	Description	Size	Expect	Ratio	Raw P value	FDR
HDL-C	GO:0055091	Phospholipid homeostasis	10	0.02	165.32	6.44E-07	4.68E-03
	GO:0006639	Acylglycerol metabolic process	119	0.22	23.154	2.22E-06	4.68E-03
	GO:0006638	Neutral lipid metabolic process	120	0.22	22.961	2.31E-06	4.68E-03
	GO:0055081	Anion homeostasis	55	0.1	40.077	2.84E-06	4.68E-03
	GO:0043691	Reverse cholesterol transport	16	0.03	103.32	2.98E-06	4.68E-03
	GO:0034375	High-density lipoprotein particle remodeling	16	0.03	103.32	2.98E-06	4.68E-03
	GO:0008203	Cholesterol metabolic process	136	0.25	20.26	4.27E-06	5.75E-03
	GO:1902652	Secondary alcohol metabolic process	141	0.26	19.541	5.10E-06	6.00E-03
	GO:0006695	Cholesterol biosynthetic process	67	0.12	32.899	6.28E-06	6.27E-03
	GO:1902653	Secondary alcohol biosynthetic process	68	0.12	32.416	6.66E-06	6.27E-03
ApoA1	GO:0006639	Acylglycerol metabolic process	119	0.18	22.09	2.97E-05	1.13E-01
	GO:0006638	Neutral lipid metabolic process	120	0.18	21.9	3.07E-05	1.13E-01
	GO:0055088	Lipid homeostasis	125	0.19	21.03	3.61E-05	1.13E-01
	GO:0055081	Anion homeostasis	55	0.08	35.84	7.79E-05	1.56E-01
	GO:0055091	Phospholipid homeostasis	10	0.02	131.41	9.95E-05	1.56E-01
	GO:0034372	Very-low-density lipoprotein particle remodeling	10	0.02	131.41	9.95E-05	1.56E-01
	GO:0034370	Triglyceride-rich lipoprotein particle remodeling	11	0.02	119.46	1.21E-04	1.63E-01
	GO:0050866	Negative regulation of cell activation	179	0.27	14.68	1.46E-04	1.71E-01
	GO:0034374	Low-density lipoprotein particle remodeling	13	0.02	101.08	1.72E-04	1.75E-01
	GO:0042632	Cholesterol homeostasis	75	0.11	26.28	1.97E-04	1.75E-01

ALS: Amyotrophic lateral sclerosis; ApoA1: apolipoprotein A1; FDR: false discovery rate; HDL-C: high-density lipoprotein cholesterol.

### Fine-mapping and colocalization analysis of amyotrophic lateral sclerosis and high-density lipoprotein cholesterol or apolipoprotein A1

We made lists of the SNPs for ALS and HDL-C or ApoA1 with 99% credibility by using each shared locus as an index SNP; the results are shown in **Additional Tables 4** and **5**.

For the colocalization analysis, we calculated the posterior probability that a shared locus was a common causal genetic variant of a correlated trait pair. We found that 3 (rs34311866, rs3803800, and rs4969182) out of 71 loci and 1 (rs17561950) out of 55 shared index SNPs that were possible common causal genetic variants between ALS and HDL-C or ApoA1, respectively (**Additional Table 6**).

### Bidirectional Mendelian randomization between lipids and amyotrophic lateral sclerosis

**Figure 4:**
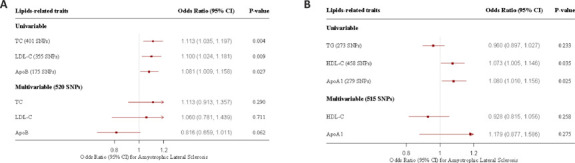
MVMR analysis of the associations between genetically predicted increased lipid levels and ALS risk. (A) Model 1, consisting of TC, LDL-C, and ApoB. (B) Model 2, consisting of HDL-C and ApoA1. ApoA1: Apolipoprotein A1; ApoB: apolipoprotein B; HDL-C: high-density lipoprotein cholesterol; LDL-C: low-density lipoprotein cholesterol; TC: total cholesterol; TG: total triglycerides.

Next, we conducted bidirectional MR analysis to identify causal associations between lipids and ALS. We extracted 273 IVs for TG, 401 IVs for TC, 458 IVs for HDL-C, 355 IVs for LDL-C, 279 IVs for ApoA1, 175 IVs for ApoB, and 12 IVs for ALS. First, we calculated the causal effect of lipids on ALS via a univariable MR approach, similar to that used in previous studies (Zeng and Zhou, 2019; van Rheenen et al., 2021; Xia et al., 2023). The results are displayed in **Additional Table 7** and **Additional Figure 1**. We found a significant causal effect of TC on ALS. LDL-C, HDL-C, ApoA1, and ApoB were nominally associated with ALS. Bidirectional MR analysis showed that ALS did not causally affect the levels of any concerned lipids (**Additional Table 8** and **Additional Figure 2**). Owing to the close association among lipid fractions, univariable MR estimates may be biased by potential pleiotropy. We therefore used a novel approach called MVMR to provide more precise evidence for causality links between blood lipids and ALS. We grouped lipid fractions according to their genetic correlation, where lipid fractions with rg > 0.6 were included in the same model. Based on this criterion, model 1 consisted of TC, LDL-C, and ApoB, and model 2 consisted of HDL-C and ApoA1. However, these results (**Table 3** and **Figure 4**) did not agree with the findings from the univariable MR analyses. No trait was causally associated with ALS in either model, indicating that the related lipid fractions acted interdependently. The MR–Egger method yielded results that were similar to those of the multivariable IVW approach.

**Additional Table 7:** Summary of effect of lipids on ALS via univariable MR methods

		TG	TC	HDL-C	LDL-C	ApoA1	ApoB
NSNPs		273	401	458	355	279	175
IVW	OR (95% CI)	0.96 (0.897, 1.027)	1.113 (1.035,1.197)	1.073 (1.005,1.146)	1.1 (1.024,1.181)	1.08(1.01,1.156)	1.081 (1.009,1.158)
	*P* value	0.233	**0.004**	**0.035**	**0.009**	**0.025**	**0.027**
Simple median	OR (95% CI)	0.955 (0.858, 1.063)	1.053 (0.942,1.178)	1.018(0.919,1.128)	1.089 (0.971, 1.221)	1.051 (0.951,1.162)	1.107 (0.99,1.237)
	*P* value	0.395	0.362	0.725	0.145	0.328	0.073
Weighted median	OR (95% CI)	0.978 (0.883, 1.083)	1.202(1.087,1.328)	1.01 (0.904,1.127)	1.089 (0.984, 1.206)	1.013(0.916,1.119)	1.017(0.922,1.121)
	*P* value	0.666	**0**	0.863	0.1	0.804	0.742
MR Egger	OR (95% CI)	1.053 (0.945, 1.174)	1.136(1.016,1.27)	1.055 (0.955,1.165)	1.104(0.987, 1.235)	1.02(0.912, 1.14)	1.016(0.913,1.13)
	*P* value	0.349	**0.025**	0.292	0.083	0.727	0.777
MR Egger	intercept	-0.003	-0.0006	0.0005	-0.0001	0.002	0.003
	*P* value	0.033	0.638	0.651	0.922	0.208	0.141
Cochran's Q	Q	360.897	571.767	635.912	487.431	396.144	206.337
	*P* value	**2.43E-04**	**3.43E-08**	**5.59E-08**	**3.10E-06**	**4.01E-06**	4.70E-02
MR-PRESSO	RS Sobs	363.118	574.641	638.455	490.095	398.808	208.268
	*P*-global test	2.00E-04	**<1E -04**	**<1E -04**	**<1E -04**	**<1E -04**	0.048
	*P*-outlier	0.321	**0.004**	**0.04**	NA	0.042	NA
	*P*-distortion	0.722	0.92	0.945	NA	0.797	NA

Bold, *P* value less than 0.05. ALS: Amyotrophic lateral sclerosis; ApoA1: apolipoprotein A1; ApoB: apolipoprotein B; CI: confidence interval; GWAS: genome-wide association study; HDL-C: high-density lipoprotein cholesterol; IVW: inverse-variance weighted method; LDL-C: low-density lipoprotein cholesterol; MR: Mendelian randomization; MR-PRESSO: Mendelian Randomization Pleiotropy RESidual Sum and Outlier; OR: odds ratio; TC: total cholesterol; TG: total triglycerides; SNP: single nucleotide polymorphism.

**Additional Table 8:** Summary of effect of ALS on lipid levels via univariable MR methods

		TG	TC	HDL-C	LDL-C	ApoA1	ApoB
NSNPs		12	12	12	12	12	12
IVW	OR (95% CI)	1.006 (0.967, 1.046)	1.002 (0.978,1.027)	0.991 (0.964, 1.019)	1.005 (0.98, 1.03)	0.994 (0.949,1.041)	1.005 (0.97,1.041)
	*P* value	0.761	0.867	0.515	0.693	0.794	0.772
Simple median	OR (95% CI)	0.989 (0.968, 1.01)	1.001 (0.986,1.016)	1.002(0.986, 1.018)	0.994 (0.977,1.011)	1.008 (0.985,1.032)	0.98 (0.955, 1.006)
	*P* value	0.312	0.91	0.814	0.469	0.495	0.129
Weighted median	OR (95% CI)	0.982 (0.964, 1)	1.006 (0.993,1.019)	1.003 (0.989, 1.018)	1.001 (0.986,1.015)	0.983 (0.964,1.002)	0.979 (0.957, 1.002)
	*P* value	0.055	0.399	0.65	0.922	0.083	0.071
MR Egger	OR (95% CI)	0.965 (0.881, 1.058)	0.999 (0.941,1.061)	0.96 (0.901,1.024)	1.006 (0.946,1.069)	0.93 (0.839, 1.031)	0.998 (0.915, 1.088)
	*P* value	0.468	0.972	0.242	0.859	0.196	0.957
MR Egger	intercept	0.005	4.00E-04	4.00E-03	-1.00E-04	0.008	0.001
	*P* value	0.35	0.91	0.312	0.982	0.19	0.852
Cochran's Q	Q	101.132	85.881	101.7	82.441	1.43E+02	75.614
	*P* value	**1.07E-16**	**1.06E-13**	**8.22E-17**	**4.97E-13**	**4.81E-25**	**1.03E-11**
MR-PRESSO	RS Sobs	114.031	95.182	119.184	94.734	164.092	92.279
	*P*-global test	**<2E-04**	**<2E-04**	**<2E-04**	**<2E-04**	**<2E-04**	**<2E-04**
	*P*-outlier	0.545	0.954	0.664	0.999	0.688	0.376
	*P*-distortion	0.164	0.059	0.158	**0.002**	0.371	0.202

Bold, *P* value less than 0.05. ALS: Amyotrophic lateral sclerosis; ApoA1: apolipoprotein A1; ApoB: apolipoprotein B; CI: confidence interval; GWAS: genome-wide association study; HDL-C: high-density lipoprotein cholesterol; IVW: inverse-variance weighted method; LDL-C: low-density lipoprotein cholesterol; MR: Mendelian randomization; MR-PRESSO: Mendelian Randomization Pleiotropy RESidual Sum and Outlier; OR: odds ratio; TC: total cholesterol; TG: total triglycerides; SNP: single nucleotide polymorphism.

**Table 3:** Summary of the effects of lipids on ALS estimated by MVMR methods

Models	Traits	IVW			MR–Egger	
		
		OR (95% CI)	*P*-value		95% CI	*P*-value
Model 1 (TC, LDL-C, ApoB)	TC	1.113 (0.913, 1.357)	0.29		1.123 (0.916, 1.377)	0.268
	LDL-C	1.06 (0.781, 1.439)	0.711		1.063 (0.781, 1.446)	0.699
	ApoB	0.816 (0.659, 1.011)	0.062		0.815 (0.659, 1.01)	0.061
Model 2 (HDL-C, ApoA1)	HDL-C	0.928 (0.815, 1.056)	0.258		0.93 (0.815, 1.06)	0.273
	ApoA1	1.179 (0.877, 1.586)	0.275		1.259 (0.855, 1.852)	0.244

ALS: Amyotrophic lateral sclerosis; ApoA1: apolipoprotein A1; ApoB: apolipoprotein B; CI: confidence interval; IVW: inverse-variance weighted method; LDL-C: low-density lipoprotein cholesterol; MR: Mendelian randomization; MVMR: multivariable Mendelian randomization; OR: odds ratio; TC: total cholesterol.

### Genetic prediction of the impact of lipid-lowering treatments on amyotrophic lateral sclerosis

Given that high lipid levels appear to increase the risk of developing ALS, next we investigated the potential of lipid-lowering drugs to prevent ALS. The overall effects of lipid level reduction driven by the target genes of lipid-lowering drugs on ALS are shown in **Table 4** and **Additional Figures 3** and **4**. Two SNPs in *ACLY* and 55 SNPs in *PCSK9* that mimicked the effects of their inhibitors lowered the risk of ALS by 90.1% (IVW-ACLY: odds ratio [OR] = 0.099, 95% confidence interval [CI] 0.012–0.854, *P* = 0.035) and 13.5% (IVW-PCSK9: OR = 0.865, 95% CI 0.774–0.966, *P* = 0.010), respectively, along with a genetically predicted SD decrease in LDL-C levels. In contrast, reducing LDL-C levels via 25 SNPs i*n HMGCR* and 14 SNPs in *NPC1L1* had no effect on ALS (IVW-HMGCR: OR = 0.971, 95% CI 0.820–1.149, *P* = 0.728; NPC1L1: OR = 0.997, 95% CI 0.663–1.499, *P* = 0.989). Considering that different lipid-lowering drugs are commonly used together in the clinic, we evaluated the efficacy of combining ACLY and PCSK9 inhibitors using a previously described method (Xiao et al., 2024). This approach protected against ALS more effectively than PCSK9 inhibitors alone (IVW: OR = 0.859, 95% CI 0.769–0.960, *P* = 0.007). Owing to the limited number of SNPs in *ACLY*, we were unable to perform further analysis of these two SNPs and ALS. Next, we performed a sensitivity analysis using lipid-lowering SNPs in non-*PCSK9* regions in ALS. Reducing LDL-C levels via LDL-C–associated SNPs outside of *PCSK9* significantly protected against ALS (IVW: OR = 0.903, 95% CI 0.882–0.924, *P* = 3.49E-18), suggesting that the effect of PCSK9 inhibitors on ALS may be partly dependent on the LDL pathway. The combination of ACLY and PCSK9 inhibitors had similar off-target effects on ALS as PCSK9 inhibitors alone (IVW: OR = 0.903, 95% CI 0.869–0.956, *P* = 2.25E-6).

**Table 4:** Summary of the effects of LDL-C lowering on ALS estimated by MR approaches

Drug-targeted genes		ACLY	HMGCR	PCSK9	NPC1L1	Combination of ACLY and PCSK9 inhibitors
N SNPs		2	25	55	14	57
IVW	OR (95% CI)	0.099 (0.012, 0.854)	0.971 (0.82, 1.149)	0.865 (0.774, 0.966)	0.997 (0.663, 1.499)	0.859 (0.769, 0.960)
	*P*-value	0.035	0.728	0.01	0.989	0.007
Simple median	OR (95% CI)	NA	0.999 (0.719, 1.386)	0.792 (0.608, 1.031)	1.172 (0.497, 2.761)	0.795 (0.600, 1.053)
	*P*-value	NA	0.994	0.089	0.722	0.115
Weighted median	OR (95% CI)	NA	0.929 (0.749, 1.151)	0.840 (0.712, 0.990)	1.145 (0.664, 1.976)	0.839 (0.705, 0.998)
	*P*-value	NA	0.499	0.038	0.625	0.048
MR-Egger	OR (95% CI)	NA	1.206 (0.726, 2.004)	1.006 (0.841, 1.202)	1.295 (0.367, 4.571)	1.025 (0.861, 1.221)
	*P*-value	NA	0.476	0.951	0.695	0.779
	intercept (*P*-value)	NA	–0.012 (0.382)	–0.008 (0.039)	–0.008 (0.676)	–0.009 (0.013)

ACLY: ATP citrate lyase; ALS: amyotrophic lateral sclerosis; CI: confidence interval; HMGCR: 3-hydroxy-3-methylglutaryl coenzyme A reductase; IVW: inverse-variance weighted method; LDL-C: low-density lipoprotein cholesterol; MR: Mendelian randomization; NA: not available; NPC1L1: proprotein convertase subtilisin/kexin type 9; OR: odds ratio; PCSK9: niemann-pick C1-like 1; SNPs: single nucleotide polymorphisms.

## Discussion

In this study, for the first time, we comprehensively evaluated the shared genetic architecture between blood lipid fractions and ALS using GWAS statistics calculated from a large sample size. Our study demonstrated that dyslipidemia and ALS within a single patient are caused in part by a shared genetic basis. We further elucidated the shared genes and common biological processes underlying these disorders, which may provide new insight into the largely unknown causes of ALS and suggest potential treatments. In addition, this work emphasized the feasibility and importance of lowering lipid levels to help prevent ALS, which may alleviate the increasing socioeconomic burden caused by the rising number of patients with ALS.

Combining the results from the genetic correlation analysis with those from the MR analysis strengthens the argument (as suggested by previous observational studies) that altered lipids metabolism and ALS are genetically associated conditions. Therefore, we interpreted our results as that the genetic association between HDL-C and ALS was driven by causality, while other ALS causally related lipid fractions may be inconsistent with the correlation of ALS in different genome regions, leading to the lack of overall genetic association. This vertical pleiotropic relationship between lipids and ALS does not necessarily imply that altered lipid levels cause ALS; rather, we suggest that increased lipid levels significantly affect the risk of developing ALS in the future or influence the response to other environmental risk factors.

Accumulating evidence has highlighted the role of HDL-C in metabolism. Our genetic correlation analysis supported the results of a recent observational study indicating that high HDL-C levels increase the risk of ALS (Bjornevik et al., 2021), and that this effect may be substantially driven by shared SNPs. Our MTAG and CPASSOC analyses showed that the highest meta-GWAS signal for HDL-C and ALS was rs72771478 near *CETP*, the gene encoding plasma cholesteryl ester transfer protein. This protein is a key enzyme involved in HDL-C remodeling and mainly binds to circulating HDL-C to mediate reverse cholesterol transport (Armitage et al., 2019). *CTEP* overexpression increases superoxide and hydrogen peroxide production, while silencing *CETP* effectively reduces ROS-mediated oxidative stress (Wanschel et al., 2021). A previous study suggested that excessive ROS production may be a pathological feature of ALS (Tam et al., 2019). The involvement of oxidative stress may partly account for the role of CETP in ALS. In our meta-analysis, the highest signal between ALS and ApoA1 was detected for rs7412 near *APOE*, and this was also the SNP with the second highest signal between ALS and HDL-C. ApoE is a major lipid transporter in the central nervous system that promotes cholesterol efflux. A high plasma ApoE level is an important risk factor for decreased survival time in patients with ALS (Lacomblez et al., 2002). It has been suggested that there is a gene dose–dependent association between APOE ε4 variants and lower age of ALS onset (Zetterberg et al., 2008), which may be related to increased TDP-43 burden (Yang et al., 2018). Praline et al. (2011) confirmed that *APOE* ε4 variants are a risk factor for bulbar-onset ALS; this association was only significant in men, possibly because ε4 variants interfere with the metabolism of androgens, which otherwise protect against ALS. However, we were unable to determine the effect of *APOE* ε4 variants on ALS risk due to the absence of critical data.

Our tissue-specific expression analysis showed that tissues enriched in both the HDL-C-ALS and ApoA1-ALS trait pairs were mainly those associated with the reproductive system and the immune system, suggesting an underlying correlation between these biological pathways and tissues. The relationships between estrogen levels or lipid peroxidation and ALS have been studied extensively, but the results are not clear. Exposure to estrogen affects ALS development, and men are more likely to develop ALS than are women (Trojsi et al., 2020). In addition, the relationship between ALS and immunity is highly intriguing. Disruption of crosstalk between the peripheral immune system and the central immune system contributes to ALS pathogenesis (Yu et al., 2022); indeed, an MR study reported that an increase in peripheral immune cell counts increases the risk of developing ALS (Li et al., 2020). Although we did not evaluate the potential of immune-related traits to act as mediators in the lipid–ALS casualty chain, our results provide circumstantial evidence for this conjecture. Furthermore, lipid metabolism and alcohol metabolism were enriched in loci shared by HDL-C and ALS. It has been suggested that alcohol consumption has a J-shaped relationship with the accumulation of lipid products (Wakabayashi, 2013) and may be a vital risk factor for ALS, as an alcohol intake of 10 g/d leads to a 1.5-fold higher risk of the disease (Yu et al., 2020). Altered responses to environmental risk factors such as alcohol intake may be part of a pathological cascade from lipids to ALS.

To test whether the observed correlation between lipids and ALS is causal, we performed a two-sample MR analysis. First, we conducted a bidirectional univariable MR analysis and found that increased TC, HDL-C, LDL-C, and apolipoprotein levels increased ALS risk, but that ALS had no effect on lipid levels; these findings mostly align with those of previous studies (Zeng and Zhou, 2019; van Rheenen et al., 2021; Xia et al., 2023). To reduce the potential effects of horizontal pleiotropy caused by overlapping IVs among different lipid fractions, we created two MVMR models based on genetic similarity. No ALS risk factors were identified among the lipid candidates, implying that similar lipid molecules do not independently affect ALS risk; rather, their interactions do. However, the results of the MVMR analysis may be influenced by the possibility of overadjustment. Considering the decrease in cholesterol content and the decreasing effect sizes of TC, LDL-C, and HDL-C on ALS, we still conservatively speculate that cholesterol is the molecular risk factor for ALS. Previous studies have shown that elevated cholesterol levels can, on the one hand, upregulate the downstream molecule oxysterol, which can cross the blood–brain barrier and affect neuronal survival (Hartmann et al., 2022). On the other hand, cholesterol can induce aggregation of proteins, thereby affecting their functions (de Meyer et al., 2010). Although altered energy metabolism in ALS has been widely reported, evidence for the value of lipids as potential biomarkers for ALS varies among studies (Mariosa et al., 2017; Bjornevik et al., 2021; Thompson et al., 2022). These clinical studies were susceptible to confounding and collinearity owing to the interdependent effects and common genetic structures of lipid fractions, as confirmed in our study. A recent study showed that ApoB is a more accurate marker than TC or LDL-C for cardiovascular events (Richardson et al., 2020). However, our results do not support this conclusion. In addition, the effect of different lipoprotein molecule particle sizes on ALS risk should be explored.

Based on our findings, we sought to identify lipid-lowering strategies that could help prevent ALS. We observed a strong effect of ACLY and PCSK9 inhibitors in terms of decreasing ALS risk. However, owing to the limited number and quality of IVs mimicking the effects of ACLY inhibitors, the impact of bempedoic acid on ALS risk should be interpreted with caution. PCSK9 can promote neuronal apoptosis by degrading ApoER2 in several cellular models (Kysenius et al., 2012). Because our sensitivity analyses of LDL-C–related variants outside of *PCSK9* also indicated a strong protective effect against ALS, the potential protective effect of PCSK9 inhibitors against ALS may be mediated by a variety of molecular pathways, including those involved in lipid metabolism. Although we found that lowering LDL-C decreases ALS risk, lowering lipid levels with statins has not been linked to a decreased risk of ALS, which could be explained by a direct effect of statins on ALS. A previous study suggested that statins can inhibit motor neuron survival by reducing mevalonate and pro-survival signals mediated by downstream isoprenoids (Alfaqih et al., 2017). A recent clinical trial found that a high-calorie diet benefits patients with rapidly progressing ALS (Ludolph et al., 2020), which seems to contradict our findings. However, according to a study describing the effect of life course body mass index on ALS risk and prognosis (Peter et al., 2017), it is possible that lipids have a similar bidirectional effect on ALS. Hartmann et al. (2022) also mentioned this controversy in a recent review, speculating that increased permeability of the blood–brain barrier in ALS may make the effect of circulating cholesterol on ALS prognosis difficult to interpret. Further studies focusing on the role of the blood–brain barrier in different ALS stages and differences in lipid regulatory/compensation mechanisms between the peripheral nervous system and central nervous system may shed light on this topic. There is no available GWAS for ALS prognosis to date; performing such a study in the future could help estimate the association between lipids and ALS prognosis. Our study was performed using data from a European population. In the future, establishing a link between genetic features and specific lipid-modification goals may guide clinical interventions. In addition, genetically predicted susceptibility to disruption of particular molecular pathways may help define clinical subgroups of ALS and, therefore, corresponding treatments.

Although we have provided comprehensive and relatively high-quality evidence for relationships among lipids, lipid-lowering drugs, and ALS, our study still has some limitations. First, both the genetic correlation and MR analyses assumed a linear relationship, and a U-shaped relationship could not be evaluated. Therefore, we are unable to identify specific lipid-lowering targets for decreasing ALS risk. Second, our results suggested that lowering lipid levels could help prevent ALS based on genetic variance reflecting the effects of lifetime exposure to a biomarker. Therefore, it remains unclear whether lipid-lowering drugs would be effective in the short term. Third, many ALS risk factors have recently been identified by MR studies with different analysis quality, including smoking, obesity, blood pressure, and education (Julian et al., 2022). We cannot deny that some IVs used in this study are associated with other traits. However, owing to the unclear molecular mechanism underlying the interaction between lipid metabolism and ALS, it is difficult to determine whether these traits are part of the vertical pleiotropic effect of lipid metabolism on ALS. In addition, since no trait has been identified that has a robust and substantial effect on ALS risk (such as that of hypertension on the risk of intracranial hemorrhage (Brott et al., 1986)), there was no basis for removing SNPs related to a particular trait. In fact, the IVs we adopted were widely dispersed across the genome and had low individual effects, reducing the possibility of introducing offsets. Therefore, to provide more powerful evidence and avoid overly conservative results, we referred to most MR studies of ALS without removing SNPs that overlapped with other traits (Julian et al., 2022). Lastly, we studied the relationship between lipids and ALS from a genetic perspective, which explained only a small proportion of their real-world correlation. Complex gene–environment interactions may account for the rest of the observed phenotypic variance (Hunter, 2005).

In conclusion, our genetic correlation analysis identified a shared genetic structure between lipids and ALS, demonstrating shared etiologies and common biological processes that contribute to abnormal lipid levels and ALS at the molecular and functional levels. MR analysis supported the hypothesis that genetically predicted elevated lipids were associated with ALS risk and that the use of ACLY or PCSK9 inhibitors may improve disease outcome. Overall, this work advances our understanding of the genetic basis of ALS and highlights the potential benefits of population-wide targeted lipid-lowering strategies in preventing ALS. In the future, studies with a larger sample size or implement other methods should be performed to further explore the beneficial effects of lipid-lowering drugs on ALS risk.

## Additional files:

***Additional Figure 1:*** *Scatterplot for the effect of lipids on ALS via univariable MR method.*

Additional Figure 1 Scatterplot for the effect of lipids on ALS via univariable MR method.
(A) Scatterplot of SNP effects on TG *versus* ALS. (B) Scatterplot of SNP effects on TC *versus* ALS. (C) Scatterplot of SNP effects on HDL-C *versus* ALS. (D) Scatterplot of SNP effects on LDL-C *versus* ALS. (E) Scatterplot of SNP effects on ApoA1 *versus* ALS. (F) Scatterplot of SNP effects on ApoB *versus* ALS. ALS: Amyotrophic lateral sclerosis; ApoA1: apolipoprotein A1; ApoB: apolipoprotein B; GWAS: genome-wide association study; HDL-C: high-density lipoprotein cholesterol; LDL-C: low-density lipoprotein cholesterol; MR: Mendelian randomization; TC: total cholesterol; TG: total triglycerides; SNP: single nucleotide polymorphism.

***Additional Figure 2:*** *Scatterplot for the effect of ALS on lipids via univariable MR method.*

Additional Figure 2 Scatterplot for the effect of ALS on lipids via univariable MR method.
(A) Scatterplot of SNP effects on ALS *versus* TG. (B) Scatterplot of SNP effects on ALS *versus* TC. (C) Scatterplot of SNP effects on ALS *versus* HDL-C. (D) Scatterplot of SNP effects on ALS *versus* LDL-C. (E) Scatterplot of SNP effects on ALS *versus* ApoA1. (F) Scatterplot of SNP effects on ALS *versus* ApoB. ALS: Amyotrophic lateral sclerosis; ApoA1: apolipoprotein A1; ApoB: apolipoprotein B; GWAS: genome-wide association study; HDL-C: high-density lipoprotein cholesterol; LDL-C: low-density lipoprotein cholesterol; MR: Mendelian randomization; TC: total cholesterol; TG: total triglycerides; SNP: single nucleotide polymorphism.

***Additional Figure 3:*** *Scatterplot of the effect of lipid-lowering interventions on ALS via univariable MR method.*

Additional Figure 3 Scatterplot of the effect of lipid-lowering interventions on ALS via univariable MR method.
(A) Scatterplot of SNP effects on ACLY versus ALS. (B) Scatterplot of SNP effects on HMGCR versus ALS. (C) Scatterplot of SNP effects on NPC1L1 versus ALS. (D) Scatterplot of SNP effects on PCSK9 versus ALS. ALS: Amyotrophic lateral sclerosis; ACLY: ATP citrate lyase; HMGCR: 3-hydroxy-3-methylglutaryl coenzyme A reductase; MR: Mendelian randomization; NPC1L1: proprotein convertase subtilisin/kexin type 9; PCSK9: Niemann-Pick C1-like 1; SNP: single nucleotide polymorphism.

***Additional Figure 4:*** *Forest plot of the effect of lipid-lowering drugs on ALS.*

Additional Figure 4 Forest plot of the effect of lipid-lowering drugs on ALS.
(A) Forest plot of NPC1L1 and ALS. (B) Forest plot of PCSK9 and ALS. (C) Forest plot of HMGCR and ALS. ALS: Amyotrophic lateral sclerosis; HMGCR: 3-hydroxy-3-methylglutaryl coenzyme A reductase; NPC1L1: proprotein convertase subtilisin/kexin type 9; PCSK9: Niemann-Pick C1-like 1.

***Additional Table 1:*** *Summary of previous studies on the values of circulating lipids on ALS as biomarkers.*

***Additional Table 2:*** *Characteristics of the shared loci between ALS and HDL-C.*

***Additional Table 3:*** *Characteristics of the shared loci between ALS and ApoA1.*

***Additional Table 4:*** *List of credible set SNPs in each shared locus between ALS and HDL-C from fine mapping.*

Additional Table 4 List of credible set SNPs in each shared locus between ALS and HDL-C from fine mapping

***Additional Table 5:*** *List of credible set SNPs in each shared locus between ALS and ApoA1 from fine mapping.*

Additional Table 5 List of credible set SNPs in each shared locus between ALS and ApoA1 from fine mapping

***Additional Table 6:*** *Co-localization analysis for loci shared between ALS and HDL-C or ApoA1.*

Additional Table 6 Co-localization analysis for loci shared between ALS and HDL-C or ApoA1

***Additional Table 7:*** *Summary of effect of lipids on ALS via univariable MR methods.*

***Additional Table 8:*** *Summary of effect of ALS on lipid levels via univariable MR methods.*

## Data Availability

*All data generated or analyzed during this study are included in this published article and its Additional files.*
