# Supplementary material for: Shared genetic link and causal inference between blood lipids, lipid-lowering drugs and amyotrophic lateral sclerosis
Source: Neural Regen Res. 2025 Aug 13;21(8):3824–30. doi: 10.4103/NRR.NRR-D-25-00266 (PMC13452702; doi:10.4103/NRR.NRR-D-25-00266)
Supplement: Supplementary file 1 [file NRR-21-3824_Suppl1.pdf]

Additional Table 4. List of credible set SNPs in each shared locus between ALS and HDL-C from fine mapping

SNP	No. credible set SNPs	List of credible set SNPs (posterior probability in brackets)
rs10774625	16	rs2013002 (1), rs847888 (0.94), rs11065987 (0.88), rs597808 (0.82), rs7137828 (0.76), rs1265564 (0.71), rs653178 (0.65), rs7310615 (0.59), rs4766578 (0.53), rs11065979 (0.47), rs11513729 (0.41), rs10774624 (0.35), rs3184504 (0.29), rs1265565 (0.24), rs4766897 (0.18), rs11065991 (0.06)
rs10853128	41	rs2227736 (1), rs11080055 (0.98), rs2239907 (0.95), rs708100 (0.93), rs12947270 (0.9), rs2239910 (0.88), rs1007398 (0.86), rs9913833 (0.83), rs1128161 (0.81), rs2073867 (0.79), rs241779 (0.76), rs7212510 (0.74), rs967645 (0.71), rs241780 (0.69), rs3093692 (0.67), rs241777 (0.64), rs3093680 (0.62), rs7212349 (0.6), rs2227735 (0.57), rs6505077 (0.55), rs241776 (0.52), rs8079943 (0.5), rs1128162 (0.48), rs4795433 (0.45), rs4795434 (0.43), rs13469 (0.38), rs2239911 (0.36), rs614877 (0.33), rs11080054 (0.31), rs2071379 (0.29), rs8081240 (0.26), rs704 (0.24), rs6505081 (0.21), rs4795435 (0.19), rs2239908 (0.17), rs733914 (0.14), rs11869677 (0.12), rs241775 (0.1), rs6505079 (0.07), rs2027993 (0.05), rs4794828 (0.02)
rs11039389	314	rs10838760 (0.3), rs11039392 (0.3), rs7111957 (0.3), rs4992357 (0.29), rs7129237 (0.29), rs12785833 (0.29), rs4752792 (0.28), rs60075622 (0.28), rs59360790 (0.28), rs11039434 (0.27), rs11039342 (0.27), rs7925299 (0.27), rs2869534 (0.26), rs6485786 (0.26), rs35985502 (0.26), rs11601603 (0.25), rs10838697 (0.25), rs1056387 (0.25), rs11039419 (0.24), rs35705029 (0.24), rs4752857 (0.24), rs4752827 (0.23), rs11039354 (0.23), rs35805829 (0.23), rs7947730 (0.22), rs11039373 (0.22), rs11039195 (0.22), rs7936948 (0.21), rs3781625 (0.21), rs11039308 (0.21), rs7107356 (0.2), rs55677087 (0.2), rs10838753 (0.2), rs12798346 (0.19), rs4752838 (0.19), rs3781627 (0.19), rs11039212 (0.19), rs4752797 (0.18), rs11039433 (0.18), rs4752990 (0.18), rs12361031 (0.17), rs10838775 (0.17), rs78187521 (0.17), rs4543938 (0.16), rs11570094 (0.16), rs2856650 (0.16), rs7942074 (0.15), rs12418852 (0.15), rs11039265 (0.15), rs7950674 (0.14), rs7941399 (0.14), rs61895760 (0.14), rs11604825 (0.13), rs1057233 (0.13), rs11039327 (0.13), rs7124681 (0.12), rs112060627 (0.12), rs2125838 (0.12), rs7112248 (0.11), rs1542321 (0.11), rs11602395 (0.11), rs11039379 (0.1), rs4752798 (0.1), rs7935521 (0.1), rs10838724 (0.09), rs10838740 (0.09), rs10769285 (0.09), rs4752801 (0.08), rs12421563 (0.08), rs10838766 (0.08), rs7925389 (0.08), rs1563821 (0.07), rs10838773 (0.07), rs7928163 (0.07), rs7110300 (0.06), rs12419692 (0.06), rs35184771 (0.06), rs11039336 (0.05), rs10838769 (0.05), rs7945473 (0.05), rs9633830 (0.04), rs12576933 (0.04), rs4752858 (0.04), rs10838768 (0.03), rs10838741 (0.03), rs3886262 (0.03), rs7114813 (0.03), rs1064608 (0.02), rs34923397 (0.02), rs12787330 (0.02), rs7120333 (0.01), rs3781626 (0.01), rs10838771 (0.01), rs10838757 (0)

rs11064373	37	rs1882551 (1), rs7134028 (0.97), rs17887160 (0.95), rs6489732 (0.92), rs2365089 (0.89), rs1997510 (0.87), rs1882550 (0.84), rs7296292 (0.82), rs11064372 (0.79), rs4764513 (0.76), rs67706382 (0.74), rs2302366 (0.71), rs11064370 (0.68), rs2286731 (0.63), rs7487291 (0.61), rs34470255 (0.58), rs2302370 (0.55), rs6489734 (0.53), rs2302368 (0.5), rs3168600 (0.47), rs7315926 (0.45), rs2365567 (0.42), rs11227 (0.39), rs2365100 (0.37), rs4764621 (0.34), rs12313899 (0.32), rs11064369 (0.29), rs4492872 (0.26), rs871365 (0.24), rs7299012 (0.21), rs59226301 (0.18), rs11064371 (0.16), rs2365092 (0.13), rs1882547 (0.11), rs7954666 (0.08), rs67385163 (0.05), rs7135106 (0.03)
rs1107850	40	rs907866 (1), rs2881925 (0.98), rs870526 (0.95), rs57096893 (0.93), rs11096646 (0.9), rs907872 (0.88), rs2137718 (0.85), rs870527 (0.83), rs35633876 (0.8), rs13010903 (0.78), rs12478327 (0.76), rs907869 (0.73), rs907868 (0.71), rs11096640 (0.68), rs11899121 (0.66), rs11895352 (0.63), rs11687037 (0.61), rs6531214 (0.59), rs6728072 (0.56), rs977130 (0.54), rs79279183 (0.51), rs7584270 (0.46), rs7569900 (0.44), rs2090034 (0.41), rs10495702 (0.39), rs6531216 (0.37), rs7577335 (0.34), rs11693335 (0.32), rs907867 (0.29), rs11687133 (0.27), rs10495703 (0.24), rs11096641 (0.22), rs13009332 (0.2), rs2090035 (0.17), rs35135293 (0.15), rs1107851 (0.12), rs7600854 (0.1), rs11894335 (0.07), rs2090032 (0.05), rs1473886 (0.02)
rs1109166	338	rs28428518 (1), rs255049 (1), rs7199699 (0.99), rs28587148 (0.99), rs113258243 (0.99), rs3826164 (0.99), rs80346622 (0.98), rs58588228 (0.98), rs7187476 (0.98), rs11555011 (0.97), rs8044328 (0.97), rs76841123 (0.97), rs56076526 (0.96), rs59708959 (0.96), rs1971546 (0.96), rs36086450 (0.96), rs112192397 (0.95), rs9934405 (0.95), rs111701446 (0.95), rs112900803 (0.94), rs148537303 (0.94), rs9928531 (0.94), rs9674089 (0.93), rs113579235 (0.93), rs113505570 (0.93), rs112358753 (0.92), rs9928783 (0.92), rs12445396 (0.92), rs7193701 (0.92), rs8047343 (0.91), rs2107369 (0.91), rs12600199 (0.91), rs111430913 (0.9), rs1074143 (0.9), rs2292316 (0.9), rs2271293 (0.89), rs2285910 (0.89), rs12600062 (0.89), rs73612208 (0.89), rs111721260 (0.88), rs111402974 (0.88), rs7187202 (0.88), rs1124993 (0.87), rs55790290 (0.87), rs7199487 (0.87), rs73597582 (0.87), rs8057119 (0.86), rs13334918 (0.86), rs112556034 (0.86), rs6499161 (0.85), rs73594556 (0.85), rs73597581 (0.85), rs7196076 (0.85), rs60638792 (0.84), rs11075670 (0.84), rs2003099 (0.84), rs118035472 (0.83), rs9923710 (0.83), rs55661503 (0.83), rs12449157 (0.82), rs117761434 (0.82), rs7199878 (0.82), rs20549 (0.82), rs8060686 (0.81), rs113563886 (0.81), rs9889207 (0.81), rs186642615 (0.8), rs73612222 (0.8), rs7187289 (0.8), rs112894876 (0.8), rs73597578 (0.79), rs12447080 (0.79), rs77996485 (0.79), rs116821144 (0.78), rs75796305 (0.78), rs73612697 (0.78), rs7203742 (0.77), rs55897859 (0.77), rs12448227 (0.77), rs76053508 (0.77), rs111934528 (0.76), rs12447931 (0.76), rs55757502 (0.76), rs147667023 (0.75), rs145158766 (0.75), rs72556537 (0.75), rs1125331 (0.75), rs35432211 (0.74), rs74629553 (0.74), rs113493894 (0.74), rs12446198 (0.73), rs28563117

(0.73), rs7193537 (0.73), rs56219053 (0.73), rs16957696 (0.72), rs113502835 (0.72), rs75909028 (0.72), rs79323429 (0.71), rs8057986 (0.71), rs9931987 (0.71), rs1134760 (0.7), rs67114979 (0.7), rs9928653 (0.7), rs6499162 (0.7), rs8044558 (0.69), rs12445533 (0.69), rs1125332 (0.69), rs56138523 (0.68), rs56374641 (0.68), rs4567701 (0.68), rs113731015 (0.68), rs12445876 (0.67), rs4359427 (0.67), rs8061500 (0.67), rs9934328 (0.66), rs1476306 (0.66), rs1985437 (0.66), rs112310696 (0.65), rs7204192 (0.65), rs113804408 (0.65), rs7191129 (0.65), rs113201209 (0.64), rs11553287 (0.64), rs10852439 (0.64), rs73597580 (0.63), rs8047978 (0.63), rs2292318 (0.63), rs78229587 (0.63), rs11075665 (0.62), rs7195605 (0.62), rs76501820 (0.62), rs8182201 (0.61), rs255053 (0.61), rs12599238 (0.61), rs8046975 (0.61), rs73591976 (0.6), rs3743735 (0.6), rs200054031 (0.6), rs73599521 (0.59), rs12444313 (0.59), rs12600144 (0.59), rs255052 (0.58), rs1975802 (0.58), rs76419201 (0.58), rs12447640 (0.57), rs7189155 (0.57), rs8046355 (0.57), rs2418739 (0.56), rs255054 (0.56), rs12446007 (0.56), rs7188900 (0.56), rs8048034 (0.55), rs73591955 (0.55), rs3809630 (0.55), rs13334205 (0.54), rs255048 (0.54), rs8044014 (0.54), rs10500543 (0.54), rs6499160 (0.53), rs8048365 (0.53), rs9888986 (0.53), rs7189381 (0.52), rs11043 (0.52), rs7199443 (0.52), rs9746872 (0.51), rs111465361 (0.51), rs9922934 (0.51), rs118005841 (0.51), rs111606836 (0.5), rs7184821 (0.5), rs12597573 (0.5), rs8060893 (0.49), rs8051587 (0.49), rs57408712 (0.49), rs2418736 (0.49), rs7198357 (0.48), rs139784051 (0.48), rs73598006 (0.48), rs12448991 (0.47), rs113790673 (0.47), rs3743739 (0.47), rs79577483 (0.46), rs140979755 (0.46), rs7194894 (0.46), rs56845922 (0.46), rs113162813 (0.45), rs2008173 (0.45), rs12444188 (0.45), rs1124324 (0.44), rs73593810 (0.44), rs12446667 (0.44), rs9939821 (0.44), rs111820418 (0.43), rs2418737 (0.43), rs7204208 (0.43), rs7193935 (0.42), rs58388686 (0.42), rs36046686 (0.42), rs56070533 (0.42), rs56230350 (0.41), rs74024145 (0.41), rs193192671 (0.41), rs56303487 (0.4), rs12598630 (0.4), rs12598274 (0.4), rs3785100 (0.39), rs12447119 (0.39), rs2290699 (0.39), rs28508024 (0.39), rs28465467 (0.38), rs113704872 (0.38), rs117751972 (0.38), rs13816 (0.37), rs60769312 (0.37), rs56047901 (0.37), rs8064114 (0.37), rs76495028 (0.36), rs55725810 (0.36), rs13339471 (0.36), rs111227007 (0.35), rs73612690 (0.35), rs8047119 (0.35), rs73594581 (0.35), rs7199588 (0.34), rs7204974 (0.34), rs9932414 (0.34), rs1125333 (0.33), rs17688076 (0.33), rs7190079 (0.33), rs16942887 (0.32), rs12599880 (0.32), rs73591968 (0.32), rs8056649 (0.32), rs112086775 (0.31), rs7206671 (0.31), rs7201591 (0.31), rs150850722 (0.3), rs6499145 (0.3), rs111315946 (0.3), rs146250480 (0.3), rs28548264 (0.29), rs112529313 (0.29), rs12448677 (0.29), rs11075669 (0.28), rs7197726 (0.28), rs8059575 (0.28), rs9938553 (0.27), rs6499143 (0.27), rs7188085 (0.27), rs112002856 (0.27), rs7189794 (0.26), rs9931366 (0.26), rs12444968 (0.26), rs79719939 (0.25), rs3785108 (0.25), rs141991634 (0.25), rs6499157 (0.25), rs113066419 (0.24), rs111967459 (0.24), rs56071820 (0.24), rs9923003 (0.23), rs9922594 (0.23), rs112434994 (0.23), rs7206792 (0.22), rs6499163

		(0.22), rs8044995 (0.22), rs7201742 (0.21), rs79375669 (0.21), rs7195172 (0.21), rs112099181 (0.2), rs12599178 (0.2), rs8048364 (0.2), rs3785098 (0.2), rs9674047 (0.19), rs7192187 (0.19), rs3815173 (0.19), rs191996617 (0.18), rs7194694 (0.18), rs10468274 (0.18), rs111948678 (0.18), rs4474673 (0.17), rs7196789 (0.17), rs76984161 (0.17), rs35723182 (0.16), rs56097211 (0.16), rs4640173 (0.16), rs2290700 (0.15), rs12448923 (0.15), rs76836953 (0.15), rs60626639 (0.15), rs4517790 (0.14), rs6499134 (0.14), rs75297764 (0.14), rs60990105 (0.13), rs73599506 (0.13), rs8059305 (0.13), rs2418735 (0.13), rs8054091 (0.12), rs7197756 (0.12), rs56377113 (0.12), rs59600980 (0.11), rs8057577 (0.11), rs75735284 (0.11), rs75286242 (0.11), rs61421564 (0.1), rs255056 (0.1), rs12598256 (0.1), rs73591961 (0.09), rs8045898 (0.09), rs12596883 (0.09), rs8046907 (0.08), rs75229939 (0.08), rs55781197 (0.08), rs3785109 (0.08), rs73612297 (0.07), rs8060967 (0.07), rs77884900 (0.07), rs2418738 (0.06), rs9938020 (0.06), rs7185308 (0.06), rs76595854 (0.06), rs55682260 (0.05), rs55699031 (0.05), rs75450962 (0.05), rs13336189 (0.04), rs111632263 (0.04), rs7205935 (0.04), rs28700885 (0.04), rs113896227 (0.03), rs16957489 (0.03), rs2271294 (0.03), rs9922165 (0.02), rs11864819 (0.02), rs78537727 (0.01), rs8058690 (0.01), rs73594554 (0.01), rs9932251 (0.01), rs76646352 (0),
rs111873781	14	rs74283344 (1), rs74473238 (0.93), rs2352969 (0.87), rs111389326 (0.8), rs150478785 (0.73), rs74665992 (0.67), rs4688688 (0.6), rs111226181 (0.53), rs6799579 (0.47), rs12107768 (0.4), rs11714324 (0.33), rs76194913 (0.2), rs2624821 (0.13), rs112524388 (0.07)
rs113629348	1	rs143102005 (0.5)
rs114614648	186	rs3733197 (1), rs4699259 (0.99), rs4530633 (0.98), rs11097760 (0.98), rs13136118 (0.97), rs2851333 (0.97), rs2851336 (0.96), rs4698979 (0.96), rs2850383 (0.95), rs12331849 (0.95), rs3974650 (0.94), rs13107612 (0.94), rs11940244 (0.93), rs13129744 (0.93), rs35416717 (0.92), rs10856963 (0.91), rs4698972 (0.91), rs11938185 (0.9), rs34499378 (0.9), rs4698971 (0.89), rs9999440 (0.89), rs2631246 (0.88), rs7679882 (0.88), rs36128945 (0.87), rs35446399 (0.87), rs80136231 (0.86), rs189908943 (0.86), rs2002758 (0.85), rs35388091 (0.85), rs34761248 (0.84), rs11940934 (0.84), rs13142517 (0.83), rs4295265 (0.82), rs2851320 (0.82), rs11944577 (0.81), rs11933157 (0.81), rs35979946 (0.8), rs138641783 (0.8), rs13119516 (0.79), rs2850387 (0.79), rs35476121 (0.78), rs13141934 (0.78), rs10446708 (0.77), rs2851328 (0.77), rs13103438 (0.76), rs6830565 (0.76), rs75088535 (0.75), rs1386176 (0.74), rs4235402 (0.74), rs10446682 (0.73), rs13434472 (0.73), rs6857428 (0.72), rs7682346 (0.72), rs34029191 (0.71), rs13126550 (0.71), rs7662572 (0.7), rs2080820 (0.7), rs4698978 (0.69), rs35838403 (0.69), rs28396651 (0.68), rs13131198 (0.68), rs35201947 (0.67), rs55768089 (0.66), rs7441177 (0.66), rs4443287 (0.65), rs13107572 (0.65), rs35748951 (0.64), rs1027013 (0.64), rs2851319 (0.63), rs12498921 (0.63), rs13101528 (0.62), rs11939568 (0.62), rs12506564 (0.61),

		rs34321727 (0.61), rs57799233 (0.6), rs28582881 (0.6), rs13135381 (0.59), rs4699258 (0.59), rs4365725 (0.58), rs1386173 (0.57), rs13128022 (0.57), rs111541987 (0.56), rs35860418 (0.56), rs7440537 (0.55), rs35194352 (0.55), rs4345184 (0.54), rs7656720 (0.54), rs2851334 (0.53), rs2631249 (0.53), rs4607219 (0.52), rs34261083 (0.52), rs11938245 (0.51), rs10013187 (0.51), rs4493533 (0.5), rs4698974 (0.49), rs1363067 (0.49), rs6850747 (0.48), rs67330256 (0.48), rs11944613 (0.47), rs13136796 (0.47), rs17266357 (0.46), rs4426778 (0.46), rs13146194 (0.45), rs13148076 (0.45), rs13152198 (0.44), rs34749007 (0.44), rs12331595 (0.43), rs11734871 (0.43), rs4463068 (0.42), rs58654655 (0.41), rs34166099 (0.41), rs17200433 (0.4), rs4639076 (0.4), rs11097757 (0.39), rs11930376 (0.39), rs34814827 (0.38), rs10016018 (0.38), rs11935577 (0.37), rs11931658 (0.37), rs1486572 (0.36), rs17266594 (0.36), rs71597109 (0.35), rs13108400 (0.35), rs2850378 (0.34), rs17031641 (0.34), rs4698839 (0.33), rs9790475 (0.32), rs2631244 (0.32), rs13116542 (0.31), rs67800004 (0.31), rs17266552 (0.3), rs923206 (0.3), rs6816787 (0.29), rs2850375 (0.29), rs3974649 (0.28), rs10446707 (0.28), rs2052445 (0.27), rs66638185 (0.27), rs4699260 (0.26), rs4270589 (0.26), rs17200824 (0.25), rs1843680 (0.24), rs34208976 (0.24), rs4637409 (0.23), rs10516483 (0.23), rs11940179 (0.22), rs11931087 (0.22), rs4572884 (0.21), rs2850377 (0.21), rs11097761 (0.2), rs4398527 (0.2), rs4496585 (0.19), rs4296682 (0.19), rs11932972 (0.18), rs976654 (0.18), rs13106926 (0.16), rs2851329 (0.16), rs4610336 (0.15), rs7686702 (0.15), rs4554079 (0.14), rs4698977 (0.14), rs143152246 (0.13), rs11736568 (0.13), rs11942437 (0.12), rs11929782 (0.12), rs10446706 (0.11), rs4699261 (0.11), rs4698975 (0.1), rs376206821 (0.1), rs11934097 (0.09), rs60448423 (0.09), rs4699262 (0.08), rs34851381 (0.07), rs1125271 (0.07), rs901406 (0.06), rs10516487 (0.06), rs2631247 (0.05), rs13124747 (0.05), rs2631248 (0.04), rs2216546 (0.04), rs4270588 (0.03), rs4698969 (0.03), rs1352174 (0.02), rs2850379 (0.02), rs1421627 (0.01), rs13117264 (0.01)
rs117186302	2	rs72918068 (1), rs72918076 (0.67),
rs117254618	4	rs117522749 (1), rs74845765 (0.8), rs78897229 (0.4), rs36049418 (0.2)
rs117323391	8	rs117953956 (1), rs143858063 (0.78), rs75242419 (0.67), rs75689126 (0.56), rs141057320 (0.44), rs117700123 (0.33), rs118166373 (0.22), rs80281960 (0.11)
rs12453334	143	rs60667221 (1), rs12943476 (0.99), rs8072548 (0.99), rs12602514 (0.98), rs2227333 (0.97), rs62068180 (0.97), rs3859187 (0.96), rs2241245 (0.95), rs4458030 (0.94), rs12602854 (0.94), rs12451547 (0.93), rs4134417 (0.92), rs62068183 (0.92), rs34648856 (0.91), rs11652139 (0.9), rs62068175 (0.9), rs3087852 (0.89), rs8077456 (0.88), rs7502971 (0.88), rs34342173 (0.87), rs8071037 (0.86), rs2227321 (0.86), rs12309 (0.85), rs3859192 (0.84), rs7502233 (0.83), rs3785549 (0.83), rs11078935 (0.82), rs2305482 (0.81), rs7222039 (0.81), rs2001476 (0.8), rs11078930 (0.79), rs3785550 (0.79), rs8078692 (0.78), rs12602513 (0.77), rs9915252

		(0.77), rs7209742 (0.76), rs17609240 (0.75), rs35982947 (0.74), rs56396280 (0.74), rs3907022 (0.73), rs62068177 (0.72), rs12453732 (0.72), rs34003767 (0.71), rs8067817 (0.7), rs28618095 (0.7), rs62068179 (0.69), rs9914973 (0.68), rs4065876 (0.68), rs12450688 (0.67), rs3935281 (0.66), rs67480438 (0.66), rs576295138 (0.65), rs543563352 (0.64), rs11870683 (0.63), rs2227319 (0.62), rs3859191 (0.61), rs1563103 (0.61), rs62068184 (0.6), rs12940405 (0.59), rs35272691 (0.59), rs4065321 (0.58), rs8075668 (0.57), rs12453343 (0.57), rs12601333 (0.56), rs62068176 (0.55), rs34670005 (0.54), rs8076474 (0.54), rs11869855 (0.53), rs8070444 (0.52), rs7207600 (0.52), rs7219080 (0.51), rs2302776 (0.5), rs11655264 (0.5), rs4134499 (0.49), rs2302777 (0.48), rs7218321 (0.48), rs11655655 (0.47), rs3848395 (0.46), rs7218742 (0.46), rs2305481 (0.45), rs8068260 (0.44), rs34540281 (0.43), rs10445310 (0.43), rs25645 (0.42), rs11078931 (0.41), rs12453341 (0.41), rs9898547 (0.4), rs4134501 (0.39), rs12939682 (0.39), rs12450800 (0.38), rs4134502 (0.37), rs2241244 (0.37), rs34933056 (0.36), rs56410675 (0.35), rs12451897 (0.34), rs12453249 (0.34), rs2302774 (0.33), rs55927420 (0.32), rs4134500 (0.31), rs7212944 (0.3), rs4072639 (0.3), rs9916158 (0.29), rs60137005 (0.28), rs62068178 (0.28), rs1007654 (0.27), rs11654208 (0.26), rs62068181 (0.26), rs3859189 (0.25), rs3816472 (0.24), rs60134943 (0.23), rs4239225 (0.23), rs56030650 (0.22), rs6503527 (0.21), rs2270401 (0.21), rs66826786 (0.2), rs11078936 (0.19), rs9889711 (0.19), rs62068174 (0.18), rs3902024 (0.17), rs12941811 (0.17), rs56326707 (0.16), rs8073254 (0.15), rs8081437 (0.14), rs12601929 (0.14), rs11658328 (0.13), rs8068522 (0.12), rs7223318 (0.12), rs11867302 (0.11), rs201474001 (0.1), rs12453764 (0.1), rs34453912 (0.09), rs2313640 (0.08), rs4433838 (0.08), rs9895948 (0.07), rs36071767 (0.06), rs1007655 (0.06), rs56301252 (0.05), rs4134498 (0.04), rs62068182 (0.03), rs9889716 (0.03), rs3744245 (0.02), rs8065126 (0.01), rs8066582 (0.01)
rs12575636	3	rs7933222 (0.75), rs4409785 (0.5), rs11021232 (0.25)
rs1515114	116	rs2943632 (1), rs2943636 (0.99), rs1813831 (0.98), rs2972155 (0.97), rs2894586 (0.97), rs2396314 (0.96), rs2972156 (0.95), rs1567973 (0.94), rs2972136 (0.93), rs2943652 (0.92), rs2972144 (0.92), rs2943647 (0.91), rs2673144 (0.9), rs2138161 (0.89), rs2713542 (0.88), rs2943644 (0.87), rs114616721 (0.86), rs201810030 (0.86), rs6436620 (0.85), rs925735 (0.84), rs2972153 (0.82), rs2203452 (0.81), rs2713557 (0.81), rs2943641 (0.8), rs1515102 (0.79), rs1399627 (0.78), rs2943645 (0.77), rs2396316 (0.76), rs1515104 (0.75), rs2943650 (0.75), rs2972150 (0.74), rs2176040 (0.73), rs2972152 (0.72), rs1515098 (0.71), rs4645008 (0.7), rs2943659 (0.69), rs2943653 (0.69), rs35787079 (0.67), rs2673134 (0.66), rs1399626 (0.65), rs2673141 (0.64), rs1399623 (0.64), rs1515108 (0.63), rs2972140 (0.62), rs2943657 (0.61), rs2943646 (0.6), rs1515116 (0.59), rs2713556 (0.58), rs2972145 (0.58), rs2673135 (0.57), rs2972137 (0.56), rs2894585 (0.55), rs1515110 (0.54), rs2943660 (0.53), rs2943658 (0.53), rs2176042

		(0.52), rs2673142 (0.51), rs2673128 (0.5), rs2713560 (0.49), rs2972143 (0.48), rs6757213 (0.47), rs2713553 (0.47), rs2713548 (0.46), rs6717669 (0.45), rs2396312 (0.44), rs2972135 (0.43), rs952227 (0.42), rs1399625 (0.42), rs2943635 (0.41), rs2713539 (0.4), rs6706153 (0.39), rs2943656 (0.38), rs2222018 (0.37), rs1515100 (0.36), rs2713540 (0.36), rs1024137 (0.35), rs1515099 (0.34), rs2673136 (0.33), rs2972147 (0.32), rs2138157 (0.31), rs2458993 (0.31), rs2943651 (0.3), rs2713544 (0.29), rs1913657 (0.28), rs2972157 (0.27), rs114562459 (0.26), rs1606791 (0.25), rs2972146 (0.25), rs908252 (0.24), rs2713549 (0.23), rs2943654 (0.22), rs2713558 (0.21), rs2943633 (0.2), rs2972138 (0.19), rs2673140 (0.19), rs2673137 (0.18), rs2713541 (0.17), rs537433434 (0.16), rs2396315 (0.15), rs4072096 (0.14), rs2943637 (0.14), rs1399624 (0.13), rs2713559 (0.12), rs2972158 (0.11), rs2713536 (0.1), rs2943640 (0.09), rs2943649 (0.08), rs2972149 (0.08), rs6713510 (0.07), rs2972154 (0.06), rs2673131 (0.05), rs2943648 (0.04), rs2713551 (0.03), rs62188784 (0.03), rs10439362 (0.02), rs13423088 (0.01)
rs16952930	42	rs55787709 (1), rs141062196 (0.98), rs62056471 (0.95), rs8066714 (0.93), rs147049164 (0.91), rs16953523 (0.88), rs62056499 (0.86), rs62056467 (0.84), rs76115469 (0.81), rs12937650 (0.79), rs71355271 (0.74), rs71355297 (0.72), rs77612020 (0.7), rs12948505 (0.67), rs12941393 (0.65), rs34448281 (0.63), rs140628747 (0.6), rs76062949 (0.58), rs55965336 (0.56), rs8077853 (0.53), rs34345377 (0.51), rs12939432 (0.49), rs10521105 (0.47), rs148766385 (0.44), rs11870735 (0.42), rs36027158 (0.4), rs71355278 (0.37), rs34311093 (0.35), rs34121882 (0.33), rs80162254 (0.3), rs34305525 (0.28), rs12951235 (0.26), rs12951098 (0.23), rs34135607 (0.21), rs12453108 (0.19), rs12948432 (0.16), rs12938449 (0.14), rs71206089 (0.12), rs71355275 (0.09), rs11558129 (0.07), rs76257494 (0.05), rs34711755 (0.02)
rs17532413	12	rs139144463 (1), rs17532371 (0.92), rs7336347 (0.85), rs55780081 (0.77), rs17447487 (0.69), rs56120794 (0.62), rs73176937 (0.54), rs117230571 (0.46), rs73176948 (0.38), rs78424108 (0.31), rs17532490 (0.23), rs17532301 (0.15)
rs17717355	7	rs188584068 (1), rs75120785 (0.88), rs75670664 (0.75), rs79067723 (0.5), rs76222503 (0.38), rs74710540 (0.25), rs76024223 (0.13)
rs2058914	279	rs11632718 (1), rs62014214 (1), rs4984307 (0.99), rs62012766 (0.99), rs12324009 (0.99), rs62012825 (0.98), rs8040757 (0.98), rs62011342 (0.98), rs12324419 (0.97), rs17766223 (0.97), rs13379688 (0.96), rs62014216 (0.96), rs12592616 (0.96), rs62011333 (0.95), rs2271241 (0.95), rs11857787 (0.95), rs8025240 (0.94), rs8037046 (0.94), rs62020761 (0.94), rs7178762 (0.93), rs62011298 (0.93), rs11639343 (0.92), rs4984253 (0.92), rs10152687 (0.91), rs55640532 (0.91), rs7175717 (0.91), rs62014167 (0.9), rs28517814 (0.9), rs11629641 (0.9), rs11631436 (0.89), rs7173984 (0.89), rs4984313 (0.89), rs1131647 (0.88), rs62012838 (0.88), rs11635472 (0.88), rs7180729 (0.87), rs28661116 (0.87), rs79076440 (0.87), rs62012833 (0.86), rs533785201 (0.86),

rs17185876 (0.85), rs62023114 (0.85), rs12708478 (0.85), rs62012765 (0.84), rs11854824 (0.84), rs12899671 (0.84), rs112593021 (0.83), rs62011335 (0.83), rs11633830 (0.83), rs76115381 (0.82), rs978363 (0.82), rs17773700 (0.82), rs62012830 (0.81), rs2228517 (0.81), rs7171858 (0.8), rs62012831 (0.8), rs117737827 (0.8), rs11635117 (0.79), rs2053594 (0.79), rs34143602 (0.79), rs17185673 (0.78), rs62014166 (0.78), rs76453046 (0.78), rs62020829 (0.77), rs17766325 (0.77), rs62014171 (0.77), rs76836538 (0.76), rs13379670 (0.76), rs62011302 (0.76), rs144877098 (0.75), rs62014165 (0.75), rs4074448 (0.74), rs17773244 (0.74), rs8035561 (0.74), rs74018158 (0.73), rs60618359 (0.73), rs982077 (0.73), rs2228510 (0.72), rs2099922 (0.72), rs4344682 (0.72), rs7177766 (0.71), rs62011325 (0.71), rs62012769 (0.71), rs56175545 (0.7), rs10438289 (0.7), rs35216087 (0.7), rs62012827 (0.69), rs11071759 (0.68), rs62012824 (0.68), rs62012773 (0.68), rs10519223 (0.67), rs62012826 (0.67), rs12914183 (0.67), rs62012782 (0.66), rs62012822 (0.66), rs75663391 (0.66), rs978362 (0.65), rs12900944 (0.65), rs11632919 (0.65), rs17186555 (0.64), rs34479262 (0.64), rs544334138 (0.63), rs12324645 (0.63), rs10152714 (0.63), rs11630289 (0.62), rs62014217 (0.62), rs4577025 (0.62), rs79566801 (0.61), rs146159842 (0.61), rs17772465 (0.61), rs12372939 (0.6), rs11639412 (0.6), rs12900621 (0.6), rs35360755 (0.59), rs11636351 (0.59), rs112305294 (0.59), rs11630720 (0.58), rs7180358 (0.58), rs62012768 (0.57), rs1470314 (0.57), rs12148660 (0.57), rs10851732 (0.56), rs7169217 (0.56), rs62012820 (0.56), rs2063281 (0.55), rs9972569 (0.55), rs62020789 (0.55), rs11071758 (0.54), rs9972527 (0.54), rs11855988 (0.54), rs56163012 (0.53), rs62014183 (0.53), rs17186681 (0.52), rs62023078 (0.52), rs11071770 (0.52), rs7172896 (0.51), rs62011344 (0.51), rs28496317 (0.51), rs4984303 (0.5), rs35943131 (0.5), rs62012829 (0.5), rs62014213 (0.49), rs79714693 (0.49), rs8040381 (0.49), rs11636758 (0.48), rs62012772 (0.48), rs11633180 (0.48), rs10152174 (0.47), rs62014215 (0.47), rs62014184 (0.46), rs7164926 (0.46), rs11071764 (0.46), rs11631176 (0.45), rs11071771 (0.45), rs11457 (0.45), rs7180823 (0.44), rs62012786 (0.44), rs1134684 (0.44), rs62020781 (0.43), rs62012775 (0.43), rs1011258 (0.43), rs62012836 (0.42), rs34675318 (0.42), rs9972479 (0.41), rs62014176 (0.41), rs1396713 (0.41), rs62014172 (0.4), rs2089619 (0.4), rs3764186 (0.4), rs4073096 (0.39), rs11635129 (0.39), rs62011346 (0.39), rs17186513 (0.38), rs3934552 (0.38), rs79730824 (0.38), rs2120252 (0.37), rs12913540 (0.37), rs35627589 (0.37), rs62022639 (0.36), rs12148623 (0.36), rs62011297 (0.35), rs62011345 (0.35), rs78029984 (0.35), rs74020820 (0.34), rs17184753 (0.34), rs78857043 (0.34), rs62014185 (0.33), rs34726252 (0.33), rs17184592 (0.33), rs10438347 (0.32), rs11631837 (0.32), rs8034342 (0.32), rs62011330 (0.31), rs17765642 (0.31), rs77715851 (0.3), rs77912104 (0.3), rs146311723 (0.3), rs11634705 (0.29), rs4984297 (0.29), rs3210904 (0.29), rs60779579 (0.28), rs12907238 (0.28), rs28627799 (0.28), rs62012837 (0.27), rs6494407 (0.27), rs62014182 (0.27), rs62014163 (0.26), rs143267946 (0.26), rs11857705 (0.26), rs12102137 (0.25), rs62012364 (0.25),

		rs80275182 (0.24), rs11635351 (0.24), rs8037694 (0.24), rs12903326 (0.23), rs62014174 (0.23), rs12102213 (0.23), rs4984249 (0.22), rs9972554 (0.22), rs77107306 (0.22), rs17185287 (0.21), rs60954093 (0.21), rs8036500 (0.21), rs12911939 (0.2), rs10152669 (0.2), rs4412925 (0.2), rs62012365 (0.19), rs62012839 (0.19), rs9972484 (0.18), rs80047978 (0.18), rs62012770 (0.18), rs2175088 (0.17), rs4536410 (0.17), rs62014164 (0.17), rs10162809 (0.16), rs34562625 (0.16), rs17184782 (0.16), rs2202867 (0.15), rs62011301 (0.15), rs61059171 (0.15), rs62011326 (0.14), rs4984251 (0.14), rs10163125 (0.13), rs79976004 (0.13), rs62012774 (0.13), rs67566652 (0.12), rs62011331 (0.12), rs16947343 (0.12), rs8034675 (0.11), rs10519213 (0.11), rs62014173 (0.11), rs7176089 (0.1), rs62014169 (0.1), rs2099920 (0.1), rs62012834 (0.09), rs576907047 (0.09), rs62014170 (0.09), rs11636604 (0.08), rs55941923 (0.08), rs62012787 (0.07), rs62012823 (0.07), rs2899695 (0.06), rs12324588 (0.06), rs10152416 (0.06), rs62011343 (0.05), rs57723091 (0.05), rs12324720 (0.05), rs16947468 (0.04), rs12912296 (0.04), rs2058913 (0.04), rs16947268 (0.03), rs4984296 (0.03), rs62012818 (0.02), rs77064122 (0.02), rs17184999 (0.02), rs17766277 (0.01), rs4984305 (0.01), rs57932859 (0.01), rs28438975 (0)
rs2112347	125	rs370654089 (1), rs4703677 (0.99), rs111937612 (0.98), rs59778668 (0.98), rs34340 (0.97), rs253393 (0.96), rs253409 (0.95), rs72633988 (0.94), rs2307113 (0.94), rs7381040 (0.93), rs373167598 (0.92), rs2047059 (0.91), rs4703679 (0.91), rs4704231 (0.9), rs3843482 (0.89), rs6453143 (0.88), rs17649362 (0.87), rs547528772 (0.87), rs42854 (0.86), rs145639541 (0.85), rs7715806 (0.84), rs34361 (0.83), rs10057967 (0.83), rs58255973 (0.82), rs2640646 (0.81), rs7381033 (0.8), rs367659190 (0.8), rs253412 (0.79), rs253414 (0.78), rs7713337 (0.77), rs253413 (0.76), rs2291630 (0.76), rs377290437 (0.75), rs253391 (0.74), rs166183 (0.73), rs6864091 (0.72), rs12109685 (0.71), rs2878419 (0.7), rs117252103 (0.69), rs253388 (0.69), rs72633987 (0.68), rs258494 (0.67), rs253415 (0.66), rs253392 (0.65), rs34359 (0.65), rs10462521 (0.64), rs10078021 (0.63), rs60493905 (0.62), rs369671918 (0.61), rs60423072 (0.61), rs4704210 (0.6), rs258495 (0.59), rs17563686 (0.58), rs66493518 (0.57), rs7732100 (0.57), rs17563863 (0.56), rs17649055 (0.55), rs4704230 (0.54), rs150247403 (0.54), rs4704235 (0.53), rs2307111 (0.52), rs144487569 (0.5), rs253407 (0.5), rs34905687 (0.49), rs6881648 (0.48), rs7730516 (0.47), rs16872768 (0.46), rs377603206 (0.46), rs10045497 (0.45), rs72633979 (0.44), rs4704234 (0.43), rs149283666 (0.43), rs72633989 (0.42), rs11749783 (0.41), rs40059 (0.4), rs7712499 (0.39), rs67054144 (0.39), rs6453131 (0.38), rs72633978 (0.37), rs6874626 (0.36), rs34341 (0.35), rs10462356 (0.35), rs17244834 (0.34), rs6868069 (0.33), rs42302 (0.32), rs253411 (0.31), rs40060 (0.31), rs11746063 (0.3), rs111647749 (0.29), rs145619427 (0.28), rs6453142 (0.28), rs61173369 (0.27), rs140583126 (0.26), rs12654264 (0.25), rs34360 (0.24), rs9942416 (0.24), rs34351 (0.23), rs1817869 (0.22), rs66787104 (0.21), rs194137 (0.2), rs888789 (0.2), rs111677149 (0.19), rs253408 (0.18), rs7707588

		(0.17), rs138490271 (0.17), rs4703678 (0.16), rs3843480 (0.15), rs4703680 (0.14), rs56912706 (0.13), rs11748378 (0.13), rs375574520 (0.12), rs10038095 (0.11), rs193344 (0.1), rs2546188 (0.09), rs6453133 (0.09), rs34358 (0.08), rs12109251 (0.07), rs3846663 (0.06), rs13356670 (0.06), rs3843481 (0.05), rs7703051 (0.04), rs41534344 (0.03), rs17563995 (0.02), rs3797580 (0.02), rs72633990 (0.01)
rs2186037	91	rs4424101 (1), rs2503326 (0.99), rs9491706 (0.98), rs9491710 (0.97), rs719726 (0.96), rs2503106 (0.95), rs7763784 (0.93), rs1936805 (0.92), rs2800710 (0.91), rs1892171 (0.9), rs2326566 (0.89), rs2800709 (0.88), rs6932207 (0.87), rs1936789 (0.86), rs4382293 (0.85), rs9482775 (0.84), rs7771990 (0.83), rs10457487 (0.82), rs1474696 (0.8), rs7775715 (0.79), rs7764034 (0.77), rs9491705 (0.76), rs1936807 (0.75), rs9491709 (0.74), rs2503322 (0.73), rs6919601 (0.72), rs2503111 (0.71), rs6915525 (0.7), rs1936806 (0.68), rs2489631 (0.67), rs4318902 (0.66), rs2326565 (0.65), rs3734626 (0.64), rs9491697 (0.63), rs2745353 (0.62), rs11154386 (0.61), rs6569474 (0.6), rs9491701 (0.59), rs2745352 (0.58), rs7741021 (0.57), rs9968918 (0.55), rs7742668 (0.54), rs1892172 (0.53), rs2489626 (0.52), rs1936801 (0.51), rs17054398 (0.5), rs1936802 (0.49), rs2154168 (0.48), rs1936800 (0.47), rs4559104 (0.46), rs7766106 (0.45), rs6916318 (0.43), rs62438503 (0.42), rs4566896 (0.41), rs6569475 (0.4), rs4404786 (0.39), rs2800708 (0.38), rs9482772 (0.37), rs2489629 (0.36), rs13204656 (0.35), rs9491698 (0.34), rs9491704 (0.33), rs1967685 (0.32), rs4509142 (0.3), rs2503320 (0.29), rs2154167 (0.28), rs9491703 (0.27), rs9482771 (0.26), rs9491696 (0.25), rs9482773 (0.24), rs2489628 (0.23), rs9968920 (0.22), rs9491702 (0.21), rs4620145 (0.2), rs2489625 (0.18), rs2489627 (0.17), rs2489630 (0.16), rs7744964 (0.15), rs2503323 (0.14), rs2489623 (0.13), rs4644087 (0.12), rs6914968 (0.11), rs9482770 (0.1), rs6938763 (0.09), rs7745274 (0.08), rs2503107 (0.07), rs9285458 (0.05), rs2503109 (0.04), rs9491699 (0.03), rs1936809 (0.02), rs9491700 (0.01)
rs2303564	165	rs34972248 (1), rs35965566 (0.99), rs7607836 (0.98), rs35122235 (0.98), rs35362395 (0.97), rs79399657 (0.96), rs71415844 (0.96), rs6714092 (0.95), rs13030236 (0.95), rs114499421 (0.94), rs188764949 (0.93), rs16859170 (0.93), rs13012678 (0.92), rs71415841 (0.92), rs35358217 (0.91), rs34734099 (0.9), rs6755376 (0.9), rs6743566 (0.89), rs35475810 (0.89), rs34100428 (0.88), rs12989964 (0.87), rs35526961 (0.87), rs13011560 (0.86), rs13003001 (0.86), rs35813908 (0.85), rs80200696 (0.84), rs77861966 (0.84), rs6750401 (0.83), rs78738375 (0.83), rs6729124 (0.82), rs35840045 (0.81), rs10498061 (0.81), rs13383705 (0.8), rs138574283 (0.8), rs35898356 (0.79), rs71415850 (0.78), rs114456768 (0.78), rs35472563 (0.77), rs3821043 (0.77), rs34104881 (0.76), rs3806556 (0.75), rs7591358 (0.75), rs35361101 (0.74), rs74738815 (0.74), rs71415849 (0.73), rs61382498 (0.72), rs2162526 (0.72), rs71415845 (0.71), rs71415853 (0.71), rs6734184 (0.7), rs13006838 (0.69), rs3731872 (0.69), rs71415843 (0.68), rs34887023 (0.68), rs35089758 (0.67), rs71415839 (0.66), rs6728701 (0.66), rs34683377 (0.65), rs1807165

		(0.65), rs13009016 (0.64), rs34735636 (0.63), rs60520272 (0.63), rs6436065 (0.62), rs1816213 (0.62), rs35059015 (0.61), rs61500159 (0.6), rs12995175 (0.6), rs13010106 (0.59), rs6711592 (0.59), rs35311533 (0.58), rs1078802 (0.57), rs36085075 (0.57), rs3821033 (0.56), rs145080501 (0.56), rs77859765 (0.55), rs3770212 (0.54), rs58018761 (0.54), rs12993599 (0.53), rs34237608 (0.53), rs6718166 (0.52), rs13014473 (0.51), rs33946522 (0.51), rs59341668 (0.5), rs3731869 (0.5), rs34313730 (0.49), rs58227790 (0.49), rs17601474 (0.48), rs35843327 (0.47), rs16859012 (0.47), rs34146317 (0.46), rs13022721 (0.46), rs6736648 (0.45), rs62191629 (0.44), rs61670831 (0.44), rs6720523 (0.43), rs4646534 (0.43), rs13011342 (0.42), rs10498059 (0.41), rs71415842 (0.41), rs13023453 (0.4), rs6756521 (0.4), rs3755040 (0.39), rs80279592 (0.38), rs35561320 (0.38), rs1863703 (0.37), rs6716642 (0.37), rs56025682 (0.35), rs71415857 (0.35), rs6709931 (0.34), rs12991539 (0.34), rs13026613 (0.33), rs71415846 (0.32), rs71415838 (0.32), rs34765129 (0.31), rs114354238 (0.31), rs71415840 (0.3), rs35795753 (0.29), rs2303562 (0.29), rs35620463 (0.28), rs35353159 (0.28), rs7558110 (0.27), rs7565544 (0.26), rs13022976 (0.26), rs35693258 (0.25), rs13032578 (0.25), rs1579401 (0.24), rs35588689 (0.23), rs11898059 (0.23), rs12991012 (0.22), rs111699701 (0.22), rs16859180 (0.21), rs35255403 (0.2), rs13032927 (0.2), rs13028814 (0.19), rs6748692 (0.19), rs6737706 (0.18), rs7572951 (0.17), rs34906275 (0.17), rs71415837 (0.16), rs73992536 (0.16), rs12998796 (0.15), rs13011338 (0.14), rs13004222 (0.14), rs35935245 (0.13), rs1803383 (0.13), rs2272192 (0.12), rs7603660 (0.11), rs34314758 (0.11), rs35021258 (0.1), rs2303563 (0.1), rs36068653 (0.09), rs35482367 (0.08), rs1996992 (0.08), rs6739509 (0.07), rs35990362 (0.07), rs35354127 (0.06), rs16859232 (0.05), rs3821044 (0.05), rs6712310 (0.04), rs2069333 (0.04), rs12989709 (0.03), rs34727952 (0.02), rs28384379 (0.02), rs35485583 (0.01), rs6748412 (0.01)
rs2434612	109	rs1428442 (1), rs2964497 (0.99), rs1651266 (0.98), rs975630 (0.97), rs2946156 (0.96), rs2162858 (0.95), rs1981973 (0.95), rs890940 (0.94), rs12520982 (0.93), rs2434264 (0.92), rs1650505 (0.91), rs970916 (0.9), rs7735131 (0.89), rs2914225 (0.88), rs2963469 (0.87), rs1835014 (0.86), rs2964501 (0.86), rs890942 (0.85), rs1023666 (0.84), rs2914229 (0.83), rs1651267 (0.82), rs2963470 (0.81), rs2946176 (0.8), rs1651270 (0.79), rs2963477 (0.78), rs2914221 (0.77), rs2419921 (0.77), rs2963480 (0.76), rs2914224 (0.75), rs890943 (0.74), rs7716662 (0.72), rs2914231 (0.71), rs748510 (0.7), rs2946163 (0.69), rs1650503 (0.68), rs2964503 (0.68), rs2901008 (0.67), rs2964484 (0.66), rs2946179 (0.65), rs2963481 (0.64), rs2914220 (0.63), rs2963434 (0.62), rs2964500 (0.61), rs2963433 (0.6), rs2963451 (0.59), rs2914233 (0.59), rs2963462 (0.57), rs2963476 (0.56), rs2946164 (0.55), rs1650502 (0.54), rs890944 (0.53), rs890939 (0.52), rs1030472 (0.51), rs56153134 (0.5), rs6881817 (0.5), rs1650504 (0.49), rs890945 (0.48), rs2914223 (0.47), rs1122249 (0.46), rs2963478 (0.45), rs2963475 (0.44), rs3934712 (0.43), rs1465573 (0.42),

		rs2964481 (0.41), rs1428444 (0.41), rs1428443 (0.4), rs2963471 (0.39), rs1615199 (0.38), rs1650506 (0.37), rs1122080 (0.36), rs2914228 (0.35), rs2419922 (0.34), rs2914222 (0.33), rs7729301 (0.32), rs2964502 (0.32), rs57371317 (0.31), rs1878936 (0.3), rs2964498 (0.29), rs2419923 (0.28), rs1017308 (0.27), rs2914219 (0.26), rs1650548 (0.25), rs1878935 (0.24), rs2963453 (0.23), rs2963474 (0.23), rs2963449 (0.22), rs1428445 (0.21), rs2963426 (0.2), rs201769762 (0.19), rs1651274 (0.18), rs1651268 (0.17), rs2963479 (0.16), rs1651269 (0.15), rs6881996 (0.14), rs1650531 (0.14), rs34023964 (0.13), rs2963468 (0.12), rs1650527 (0.11), rs1651272 (0.1), rs7701375 (0.09), rs2419909 (0.08), rs2946160 (0.07), rs2914226 (0.06), rs3111141 (0.05), rs2946162 (0.05), rs2963463 (0.04), rs2963445 (0.03), rs2963472 (0.02), rs2964496 (0.01)
rs2492816	69	rs2484319 (1), rs10812610 (0.99), rs774354 (0.97), rs774349 (0.96), rs10812611 (0.94), rs3849945 (0.91), rs10812605 (0.9), rs4609281 (0.89), rs12350076 (0.87), rs3736319 (0.86), rs10812609 (0.84), rs7864595 (0.83), rs11795154 (0.81), rs3202601 (0.8), rs10738775 (0.79), rs812858 (0.77), rs2453565 (0.76), rs2477523 (0.74), rs2453555 (0.73), rs773720 (0.71), rs10757664 (0.7), rs774355 (0.69), rs4879541 (0.67), rs868856 (0.66), rs2814708 (0.64), rs2453554 (0.63), rs10967976 (0.61), rs7469146 (0.6), rs774351 (0.59), rs7019847 (0.57), rs903603 (0.56), rs774352 (0.54), rs1537712 (0.53), rs2814706 (0.51), rs2484322 (0.5), rs3849937 (0.49), rs10812604 (0.47), rs7036117 (0.46), rs10738774 (0.44), rs3849942 (0.43), rs774357 (0.41), rs774353 (0.4), rs1319236 (0.39), rs1845699 (0.37), rs700828 (0.36), rs10812603 (0.34), rs10435784 (0.33), rs2484314 (0.31), rs7046653 (0.3), rs3849943 (0.29), rs1982915 (0.27), rs2783010 (0.26), rs2492817 (0.24), rs12345062 (0.23), rs774359 (0.21), rs700791 (0.2), rs2814707 (0.19), rs7041409 (0.17), rs4879540 (0.16), rs7029905 (0.14), rs7868845 (0.13), rs10967993 (0.11), rs3849938 (0.1), rs2244606 (0.09), rs10967973 (0.07), rs2484316 (0.06), rs2453556 (0.04), rs2453557 (0.03), rs774356 (0.01)
rs2540948	115	rs2052261 (1), rs2901749 (0.98), rs13006381 (0.97), rs2723086 (0.97), rs1228 (0.96), rs4671120 (0.95), rs1420185 (0.94), rs1420184 (0.93), rs113401532 (0.92), rs6736728 (0.91), rs1009358 (0.91), rs62140432 (0.9), rs984748 (0.89), rs12472873 (0.88), rs10197530 (0.87), rs12713532 (0.86), rs60136636 (0.85), rs2723085 (0.85), rs4671638 (0.84), rs6730986 (0.83), rs2723064 (0.82), rs7559813 (0.81), rs7603736 (0.8), rs11888531 (0.79), rs12713533 (0.79), rs11678917 (0.78), rs11893423 (0.77), rs74181299 (0.76), rs7579489 (0.75), rs6741255 (0.74), rs6546128 (0.74), rs12472718 (0.73), rs11126029 (0.72), rs1050676 (0.71), rs2723083 (0.7), rs2723065 (0.69), rs6546123 (0.68), rs75678687 (0.68), rs2540971 (0.67), rs6751786 (0.66), rs2540949 (0.65), rs2723067 (0.64), rs2422436 (0.63), rs4671119 (0.62), rs6705891 (0.62), rs6736982 (0.61), rs10173422 (0.6), rs2302631 (0.59), rs58342673 (0.58), rs7607025 (0.57), rs6546131 (0.56), rs6713746 (0.56), rs2723075 (0.55), rs13421845 (0.54), rs11126031 (0.53), rs2540945 (0.52), rs12465024 (0.5), rs6728523 (0.5), rs4671116 (0.49), rs6715178 (0.48), rs2540950 (0.47),

		rs1009360 (0.46), rs2540951 (0.45), rs13023859 (0.44), rs7607039 (0.44), rs6546130 (0.43), rs9989843 (0.42), rs2723066 (0.41), rs12713534 (0.4), rs2723091 (0.39), rs2422437 (0.38), rs6750146 (0.38), rs6756585 (0.37), rs2241161 (0.36), rs2422438 (0.35), rs10209083 (0.34), rs2249105 (0.33), rs3732096 (0.32), rs2302647 (0.32), rs6722972 (0.31), rs11684110 (0.3), rs2723063 (0.29), rs1420183 (0.28), rs7563881 (0.27), rs2252867 (0.26), rs2080385 (0.26), rs13005414 (0.25), rs6748462 (0.24), rs2422441 (0.23), rs17040050 (0.22), rs17849707 (0.21), rs11896168 (0.21), rs7565943 (0.2), rs10195785 (0.19), rs1894875 (0.18), rs10205598 (0.17), rs2723082 (0.16), rs11126028 (0.15), rs9797989 (0.15), rs12621608 (0.14), rs7590690 (0.13), rs6546129 (0.12), rs2723087 (0.11), rs7563866 (0.1), rs2241160 (0.09), rs12990465 (0.09), rs10173678 (0.08), rs4671117 (0.07), rs2540970 (0.06), rs4671640 (0.05), rs2723062 (0.04), rs71424153 (0.03), rs1055903 (0.03), rs4671118 (0.02), rs10185243 (0.01)
rs267738	21	rs17599629 (1), rs35427932 (0.95), rs11204676 (0.91), rs2925738 (0.86), rs267734 (0.82), rs198325 (0.77), rs41305070 (0.68), rs143976390 (0.64), rs35782301 (0.59), rs72702521 (0.55), rs66958685 (0.5), rs72992015 (0.45), rs607518 (0.41), rs34593358 (0.36), rs2055975 (0.32), rs61386199 (0.27), rs56661631 (0.23), rs267733 (0.18), rs2925739 (0.14), rs75056606 (0.09), rs78132593 (0.05)
rs2792751	61	rs1129555 (1), rs2803609 (0.98), rs2263608 (0.97), rs2792743 (0.95), rs2792703 (0.94), rs7089231 (0.92), rs10736219 (0.9), rs2792759 (0.89), rs2297992 (0.87), rs4918719 (0.85), rs7899096 (0.84), rs6585139 (0.82), rs2255141 (0.81), rs2254537 (0.79), rs4917627 (0.77), rs5024318 (0.76), rs1986438 (0.74), rs7084039 (0.73), rs7897449 (0.71), rs2803611 (0.69), rs10787428 (0.68), rs4918720 (0.66), rs2277207 (0.65), rs2803608 (0.63), rs10749111 (0.61), rs34820178 (0.6), rs2792736 (0.58), rs10787427 (0.56), rs2255400 (0.55), rs72836628 (0.53), rs2297991 (0.52), rs2803619 (0.5), rs7096937 (0.48), rs6585140 (0.47), rs4917628 (0.45), rs2803621 (0.44), rs3824627 (0.4), rs2254532 (0.39), rs10749109 (0.37), rs1807800 (0.35), rs2803606 (0.34), rs2487294 (0.32), rs2803616 (0.31), rs2251579 (0.29), rs10749108 (0.27), rs3824626 (0.26), rs10787429 (0.24), rs10749113 (0.23), rs2792735 (0.21), rs2419604 (0.19), rs10749110 (0.18), rs2250802 (0.16), rs7898897 (0.15), rs10749112 (0.13), rs2792744 (0.11), rs58175211 (0.1), rs4918722 (0.08), rs2251697 (0.06), rs6585138 (0.05), rs10885314 (0.03), rs2900942 (0.02)
rs28412876	91	rs17709919 (1), rs619726 (0.99), rs2960171 (0.98), rs71379351 (0.97), rs12953014 (0.96), rs9916779 (0.95), rs4794032 (0.93), rs4794036 (0.92), rs8081878 (0.91), rs4277393 (0.9), rs12940887 (0.89), rs9909827 (0.88), rs8075803 (0.87), rs12937404 (0.86), rs2906095 (0.85), rs12150526 (0.84), rs917054 (0.83), rs1124881 (0.82), rs850520 (0.8), rs4566223 (0.79), rs2411977 (0.78), rs11079867 (0.77), rs7225787 (0.76), rs2906101 (0.75), rs12948660 (0.74), rs55634267 (0.73), rs2960164 (0.72), rs3179840 (0.71), rs12950511 (0.7), rs28618326 (0.68), rs62076398 (0.67), rs28483960 (0.66), rs35073649 (0.65), rs35317317 (0.64),

		rs7225606 (0.63), rs55978930 (0.62), rs12602769 (0.61), rs28397663 (0.6), rs12185242 (0.59), rs11655466 (0.58), rs34255993 (0.57), rs4444384 (0.55), rs4794035 (0.54), rs747039 (0.53), rs28406364 (0.52), rs35406341 (0.51), rs72699 (0.5), rs2960169 (0.49), rs12943264 (0.48), rs9893777 (0.47), rs112502960 (0.46), rs9889262 (0.45), rs6504608 (0.43), rs686112 (0.42), rs646876 (0.41), rs4794034 (0.4), rs7207729 (0.39), rs8067459 (0.38), rs28394864 (0.37), rs7213090 (0.36), rs12937634 (0.35), rs7406234 (0.34), rs11079866 (0.33), rs4793614 (0.32), rs2240301 (0.3), rs17637472 (0.29), rs57345398 (0.28), rs11079868 (0.27), rs35587648 (0.26), rs34666276 (0.25), rs850521 (0.24), rs2011767 (0.23), rs2107634 (0.22), rs663576 (0.21), rs16948048 (0.2), rs12952581 (0.18), rs12949836 (0.17), rs4514717 (0.16), rs4794029 (0.15), rs12951873 (0.14), rs2906093 (0.13), rs62076439 (0.12), rs4644893 (0.11), rs1010322 (0.1), rs9899665 (0.09), rs12940278 (0.08), rs9895986 (0.07), rs7209400 (0.05), rs2960170 (0.03), rs7222840 (0.02), rs4794031 (0.01)
rs28850399	12	rs74256604 (1), rs28773614 (0.92), rs74256605 (0.85), rs56361256 (0.77), rs10421380 (0.69), rs74578418 (0.62), rs721891 (0.46), rs17761883 (0.38), rs28853598 (0.31), rs2191668 (0.23), rs2109657 (0.15), rs76748600 (0.08)
rs34311866	53	rs12651368 (1), rs62297063 (0.98), rs9994159 (0.96), rs11248059 (0.94), rs187658856 (0.93), rs62294484 (0.91), rs11248063 (0.89), rs28628421 (0.87), rs11726280 (0.85), rs55728587 (0.83), rs17783233 (0.81), rs4583705 (0.8), rs17855876 (0.78), rs11248060 (0.76), rs11248062 (0.74), rs2279181 (0.72), rs3755961 (0.7), rs28662837 (0.69), rs6842808 (0.67), rs62294519 (0.65), rs11726912 (0.63), rs546806869 (0.61), rs11732965 (0.59), rs12651271 (0.57), rs11733713 (0.56), rs11248057 (0.54), rs11730111 (0.52), rs34006051 (0.5), rs3755958 (0.48), rs62294491 (0.46), rs78590562 (0.44), rs3775126 (0.43), rs11248051 (0.41), rs2279182 (0.39), rs11728344 (0.37), rs62297061 (0.35), rs3755960 (0.33), rs748483 (0.31), rs62297087 (0.3), rs11725653 (0.28), rs3736089 (0.26), rs11734537 (0.24), rs10029038 (0.22), rs3775122 (0.2), rs11729777 (0.19), rs1564282 (0.17), rs6599388 (0.15), rs62297062 (0.13), rs10516158 (0.11), rs17781499 (0.09), rs370517469 (0.07), rs11726637 (0.06), rs11731387 (0.02)
rs35390907	25	rs12981073 (1), rs35252095 (0.96), rs11882029 (0.92), rs7257729 (0.88), rs11260079 (0.85), rs7248298 (0.81), rs11260080 (0.77), rs12982228 (0.73), rs11669559 (0.69), rs1807414 (0.65), rs737229 (0.62), rs12979153 (0.58), rs34985787 (0.54), rs2017507 (0.5), rs7249069 (0.46), rs7259802 (0.42), rs534256261 (0.38), rs34915321 (0.35), rs2227288 (0.27), rs11667076 (0.23), rs8106699 (0.19), rs34377224 (0.15), rs35943159 (0.12), rs737228 (0.08), rs374246306 (0.04)
rs3798241	23	rs3777424 (1), rs3777425 (0.96), rs2351598 (0.92), rs113668543 (0.83), rs9374588 (0.79), rs195533 (0.75), rs2351597 (0.71), rs195468 (0.67), rs195509 (0.63), rs195515 (0.58), rs6906127 (0.54), rs3798244 (0.5), rs12662901 (0.46), rs171866 (0.42), rs523101 (0.38), rs195491 (0.33), rs9374587 (0.29), rs375387 (0.25), rs3798243 (0.21), rs9320552 (0.17), rs9387379 (0.13),

		rs7762044 (0.08), rs184128865 (0.04)
rs3800406	66	rs28567450 (1), rs11759235 (0.99), rs1555773 (0.97), rs9469907 (0.96), rs9462025 (0.94), rs9462029 (0.93), rs11755266 (0.91), rs75398113 (0.9), rs2744972 (0.88), rs9469897 (0.87), rs11756392 (0.85), rs113310749 (0.84), rs9469913 (0.82), rs9469910 (0.81), rs2395607 (0.79), rs2815005 (0.78), rs9469860 (0.76), rs9296130 (0.75), rs4713824 (0.73), rs112735232 (0.72), rs7762485 (0.7), rs9469911 (0.69), rs9654581 (0.67), rs60643641 (0.66), rs2814962 (0.63), rs2814960 (0.61), rs2744947 (0.6), rs2764204 (0.58), rs56693020 (0.57), rs73419621 (0.55), rs6911549 (0.54), rs73419607 (0.52), rs114863007 (0.51), rs2744942 (0.49), rs112826162 (0.48), rs3822921 (0.46), rs9469900 (0.45), rs9469890 (0.43), rs61431557 (0.42), rs6930421 (0.4), rs34523546 (0.39), rs1883638 (0.37), rs9462012 (0.36), rs7755822 (0.34), rs2764203 (0.33), rs56930444 (0.31), rs6936177 (0.3), rs112563428 (0.28), rs36018387 (0.27), rs2764205 (0.25), rs2814948 (0.24), rs3800457 (0.22), rs9462031 (0.21), rs6457790 (0.19), rs9462014 (0.18), rs4472337 (0.16), rs2492859 (0.15), rs9462016 (0.13), rs6938239 (0.12), rs16894959 (0.1), rs9469895 (0.09), rs9688715 (0.07), rs9462007 (0.06), rs13205738 (0.04), rs59142559 (0.03), rs9469864 (0.01)
rs3803800	21	rs9900162 (1), rs75853747 (0.95), rs8073177 (0.91), rs11078694 (0.86), rs7358 (0.77), rs77294902 (0.73), rs9908405 (0.68), rs111633209 (0.64), rs9902027 (0.59), rs1143015 (0.55), rs9892862 (0.5), rs114255812 (0.45), rs9913914 (0.41), rs9896688 (0.36), rs28655557 (0.32), rs6503039 (0.27), rs11651783 (0.23), rs60920983 (0.18), rs11656013 (0.14), rs4602096 (0.09), rs9897535 (0.05)
rs3808184	195	rs43027 (1), rs172507 (0.99), rs7777807 (0.99), rs4727851 (0.98), rs13245795 (0.98), rs7805063 (0.97), rs4458806 (0.97), rs4730795 (0.96), rs34927137 (0.96), rs2246450 (0.95), rs6977665 (0.95), rs12333572 (0.94), rs13438309 (0.94), rs1208525 (0.93), rs1429568 (0.93), rs213976 (0.92), rs34637736 (0.92), rs213986 (0.91), rs885993 (0.9), rs994997 (0.9), rs718829 (0.89), rs6958655 (0.89), rs149416044 (0.88), rs214152 (0.88), rs2082056 (0.87), rs35216437 (0.87), rs213950 (0.86), rs213961 (0.86), rs117243 (0.85), rs6949974 (0.85), rs713134 (0.84), rs213988 (0.84), rs2051718 (0.83), rs10953848 (0.83), rs4730797 (0.82), rs10487367 (0.82), rs9792104 (0.81), rs213968 (0.81), rs11975114 (0.8), rs11975043 (0.8), rs17140297 (0.79), rs4727855 (0.79), rs213987 (0.78), rs213967 (0.78), rs11974545 (0.77), rs2059938 (0.77), rs213945 (0.76), rs214159 (0.75), rs2402229 (0.75), rs2023708 (0.74), rs213933 (0.74), rs6972168 (0.73), rs6979652 (0.73), rs1208524 (0.72), rs7789741 (0.72), rs725188 (0.71), rs213942 (0.71), rs213989 (0.7), rs975722 (0.7), rs2213958 (0.69), rs6966836 (0.69), rs17140352 (0.68), rs213965 (0.68), rs43035 (0.67), rs17139943 (0.66), rs11772396 (0.66), rs214160 (0.65), rs213953 (0.65), rs177069 (0.64), rs10441245 (0.64), rs1042077 (0.63), rs35771577 (0.63), rs213949 (0.62), rs6978581 (0.62), rs213964 (0.61), rs4500028 (0.61), rs1208528 (0.6), rs6955320

		(0.6), rs39316 (0.59), rs43025 (0.59), rs10276533 (0.58), rs213979 (0.58), rs43032 (0.57), rs10266116 (0.57), rs4730793 (0.56), rs6966838 (0.56), rs17549557 (0.55), rs43036 (0.55), rs12534129 (0.54), rs6970681 (0.54), rs213963 (0.53), rs213951 (0.53), rs11768272 (0.52), rs2518873 (0.52), rs756665 (0.51), rs43033 (0.51), rs6466612 (0.5), rs2237722 (0.5), rs11978434 (0.49), rs213983 (0.49), rs994996 (0.48), rs76041288 (0.48), rs43029 (0.47), rs7804816 (0.47), rs6957018 (0.46), rs6973257 (0.46), rs213944 (0.45), rs43026 (0.45), rs12534233 (0.44), rs213966 (0.44), rs4730794 (0.43), rs10249651 (0.43), rs39318 (0.42), rs6466611 (0.42), rs2518881 (0.41), rs35050470 (0.41), rs213936 (0.4), rs213977 (0.4), rs43034 (0.39), rs2157946 (0.39), rs214163 (0.38), rs10255917 (0.38), rs73480753 (0.37), rs1800136 (0.37), rs7784156 (0.36), rs34256873 (0.36), rs10225824 (0.35), rs177068 (0.35), rs34331324 (0.34), rs213970 (0.34), rs1366912 (0.33), rs1042180 (0.32), rs2896224 (0.32), rs11767185 (0.31), rs2078967 (0.31), rs213971 (0.3), rs35292549 (0.3), rs213974 (0.29), rs35403869 (0.29), rs213934 (0.28), rs1594189 (0.28), rs6967714 (0.27), rs10264075 (0.27), rs213984 (0.26), rs10278130 (0.26), rs214167 (0.25), rs10272233 (0.25), rs34665848 (0.24), rs43031 (0.24), rs4148711 (0.23), rs213948 (0.23), rs214165 (0.22), rs2106155 (0.22), rs213981 (0.21), rs2518879 (0.21), rs2188555 (0.2), rs213952 (0.2), rs214161 (0.19), rs4727849 (0.19), rs214164 (0.18), rs3735645 (0.18), rs213957 (0.17), rs11764070 (0.17), rs213935 (0.16), rs6960356 (0.16), rs2188150 (0.15), rs177284 (0.15), rs1468262 (0.14), rs6466619 (0.14), rs980574 (0.13), rs213943 (0.13), rs4730779 (0.12), rs213938 (0.12), rs6977789 (0.11), rs213980 (0.11), rs3926643 (0.1), rs1208526 (0.1), rs13222797 (0.09), rs10953846 (0.09), rs213946 (0.08), rs35941557 (0.08), rs213985 (0.07), rs213947 (0.07), rs7357267 (0.06), rs213937 (0.06), rs43030 (0.05), rs213973 (0.05), rs6980032 (0.04), rs11976705 (0.04), rs43028 (0.03), rs6943105 (0.03), rs4730798 (0.02), rs6943317 (0.02), rs177070 (0.01), rs7810506 (0.01)
rs3808461	177	rs7834300 (1), rs3802219 (0.99), rs2009382 (0.99), rs1483583 (0.98), rs12677858 (0.98), rs4599845 (0.97), rs2737225 (0.97), rs11785504 (0.96), rs2625674 (0.96), rs3779878 (0.95), rs2721932 (0.94), rs800883 (0.94), rs800880 (0.93), rs2737227 (0.93), rs2721926 (0.92), rs3802222 (0.92), rs2178949 (0.91), rs1963678 (0.91), rs1384812 (0.9), rs10505256 (0.89), rs2625675 (0.89), rs2245019 (0.88), rs10093834 (0.88), rs3808456 (0.87), rs6469606 (0.87), rs3849850 (0.86), rs800887 (0.85), rs6986967 (0.85), rs997806 (0.84), rs3808440 (0.84), rs800881 (0.83), rs1180647 (0.83), rs2103481 (0.82), rs1569364 (0.82), rs3808451 (0.81), rs6469603 (0.8), rs13266941 (0.8), rs800871 (0.79), rs7011494 (0.79), rs2049867 (0.78), rs1351744 (0.78), rs12549788 (0.77), rs3808437 (0.77), rs6998144 (0.76), rs13271228 (0.75), rs3808457 (0.75), rs12543799 (0.74), rs800909 (0.74), rs2884366 (0.73), rs13267535 (0.73), rs2293889 (0.72), rs4326410 (0.72), rs7832568 (0.71), rs719422 (0.7), rs3802220 (0.7), rs2737207 (0.69), rs800879 (0.69), rs2223103 (0.68), rs3808450 (0.68), rs1180624 (0.67), rs3808418 (0.66), rs2245221 (0.66), rs2721954 (0.65),

		rs6990008 (0.65), rs800884 (0.64), rs800888 (0.64), rs2737205 (0.63), rs2200218 (0.63), rs9297541 (0.62), rs7825679 (0.61), rs3824200 (0.61), rs2358019 (0.6), rs2170740 (0.6), rs2142331 (0.59), rs2178946 (0.59), rs3808435 (0.58), rs3802221 (0.58), rs3808444 (0.57), rs28447075 (0.56), rs2721933 (0.56), rs2721948 (0.55), rs1079822 (0.55), rs3808448 (0.54), rs6998448 (0.54), rs3808425 (0.53), rs3808428 (0.52), rs10093833 (0.51), rs2721939 (0.51), rs12056626 (0.5), rs2721938 (0.5), rs11989423 (0.49), rs2272619 (0.49), rs3808430 (0.48), rs3808460 (0.47), rs3808446 (0.46), rs10955752 (0.46), rs13269499 (0.45), rs6469601 (0.45), rs800870 (0.44), rs3779881 (0.44), rs3808426 (0.43), rs10097784 (0.42), rs1180648 (0.42), rs1963677 (0.41), rs2737215 (0.41), rs3808438 (0.4), rs7813434 (0.4), rs2737219 (0.39), rs2737217 (0.39), rs1483585 (0.38), rs3808447 (0.37), rs11778934 (0.37), rs1483589 (0.36), rs3808422 (0.36), rs800878 (0.35), rs3808454 (0.35), rs800882 (0.34), rs2737212 (0.34), rs6469597 (0.33), rs6469605 (0.32), rs12548221 (0.32), rs2721943 (0.31), rs2170741 (0.31), rs3808439 (0.3), rs11786044 (0.3), rs2178948 (0.29), rs800908 (0.28), rs2721936 (0.28), rs2721942 (0.27), rs1483582 (0.27), rs6993253 (0.26), rs800869 (0.26), rs12679749 (0.25), rs7012216 (0.25), rs800886 (0.24), rs2003643 (0.23), rs2721934 (0.23), rs2721929 (0.22), rs2358021 (0.22), rs800868 (0.21), rs2049865 (0.21), rs1028274 (0.2), rs809623 (0.2), rs2625672 (0.19), rs3779879 (0.18), rs9643105 (0.18), rs10955753 (0.17), rs10505258 (0.17), rs3808449 (0.16), rs2737203 (0.16), rs10955751 (0.15), rs800910 (0.15), rs2737216 (0.14), rs2178947 (0.13), rs2737206 (0.13), rs3808416 (0.12), rs1057517 (0.12), rs10808475 (0.11), rs2737220 (0.11), rs2737226 (0.1), rs2721946 (0.09), rs2625673 (0.09), rs2737214 (0.08), rs1384810 (0.08), rs3808415 (0.07), rs1905376 (0.07), rs13275142 (0.06), rs1160333 (0.06), rs12114740 (0.05), rs3808459 (0.04), rs3808436 (0.04), rs3808434 (0.03), rs7357472 (0.03), rs2049864 (0.02), rs2721940 (0.02), rs6469600 (0.01), rs7823575 (0.01)
rs3809114	2	rs3809113 (1), rs703845 (0.33)
rs3819166	17	rs2000812 (1), rs1943678 (0.94), rs3829632 (0.89), rs10083905 (0.83), rs8099312 (0.78), rs4245232 (0.72), rs56341437 (0.67), rs4556888 (0.61), rs75833060 (0.56), rs7237346 (0.5), rs9958947 (0.44), rs4121823 (0.39), rs8091573 (0.33), rs4939875 (0.28), rs4939583 (0.17), rs6507929 (0.11), rs7230119 (0.06)
rs4675812	10	rs905661 (1), rs13033734 (0.91), rs871375 (0.82), rs11684339 (0.73), rs2012385 (0.64), rs9679726 (0.55), rs9288726 (0.45), rs2055568 (0.36), rs6437249 (0.27), rs905660 (0.09)
rs4855447	112	rs13314892 (1), rs115233187 (0.99), rs9817946 (0.98), rs13318796 (0.97), rs56927902 (0.96), rs79563518 (0.96), rs57317066 (0.95), rs62252234 (0.94), rs62251025 (0.93), rs1549746 (0.92), rs56386221 (0.91), rs62251019 (0.9), rs1430604 (0.89), rs55738162 (0.89), rs57788691 (0.88), rs62252237 (0.87), rs62251032 (0.86), rs74945202 (0.85), rs79151317 (0.84), rs79658882

		(0.83), rs79502300 (0.82), rs74956589 (0.82), rs1549745 (0.81), rs62253177 (0.8), rs57829272 (0.79), rs12714757 (0.78), rs6800508 (0.77), rs58529025 (0.76), rs1897159 (0.75), rs6802804 (0.75), rs75308677 (0.74), rs6772383 (0.73), rs62250981 (0.72), rs62253174 (0.71), rs62253182 (0.7), rs59487570 (0.69), rs62253180 (0.68), rs62252232 (0.68), rs62251033 (0.67), rs6805306 (0.66), rs1367370 (0.65), rs60438183 (0.64), rs962775 (0.63), rs58244283 (0.62), rs79375047 (0.61), rs62252182 (0.6), rs74491124 (0.59), rs74280296 (0.58), rs62250976 (0.57), rs55735918 (0.56), rs62250980 (0.55), rs75300766 (0.54), rs113374742 (0.54), rs62253185 (0.53), rs59846970 (0.52), rs62252174 (0.51), rs79366329 (0.5), rs62252233 (0.49), rs2315497 (0.48), rs57078287 (0.47), rs2014255 (0.46), rs62251029 (0.46), rs56764448 (0.45), rs55890624 (0.44), rs57537586 (0.43), rs62253183 (0.42), rs55947901 (0.41), rs62253176 (0.4), rs79150208 (0.39), rs56007401 (0.39), rs62251027 (0.38), rs2114660 (0.37), rs62251040 (0.36), rs62252229 (0.35), rs4855323 (0.34), rs58711181 (0.33), rs60843510 (0.32), rs2163326 (0.32), rs62251026 (0.31), rs62252239 (0.3), rs62252226 (0.29), rs56155813 (0.28), rs60551165 (0.27), rs62253179 (0.26), rs17006419 (0.25), rs7623486 (0.25), rs56099248 (0.24), rs62252218 (0.23), rs61148813 (0.22), rs62251009 (0.21), rs62252219 (0.2), rs62253178 (0.19), rs62252225 (0.18), rs62251030 (0.18), rs17638538 (0.17), rs62253181 (0.16), rs62250982 (0.15), rs62252240 (0.14), rs62250978 (0.13), rs62250970 (0.12), rs62250977 (0.11), rs62252228 (0.11), rs62252238 (0.1), rs17043851 (0.09), rs56286049 (0.08), rs57634646 (0.07), rs9869047 (0.06), rs62252230 (0.04), rs78094954 (0.04), rs55946371 (0.03), rs62251031 (0.02), rs55808455 (0.01)
rs4938340	245	rs7941428 (1), rs77250716 (1), rs61907604 (0.99), rs76904522 (0.99), rs113030965 (0.98), rs112120205 (0.98), rs112111936 (0.98), rs61905687 (0.97), rs80259223 (0.97), rs11600552 (0.96), rs7941927 (0.96), rs17120347 (0.96), rs67201490 (0.95), rs11216244 (0.95), rs57582454 (0.94), rs17120237 (0.94), rs7935913 (0.94), rs1473327 (0.93), rs4620758 (0.93), rs59097294 (0.92), rs7107702 (0.92), rs1531707 (0.91), rs7939934 (0.91), rs11216162 (0.9), rs11823918 (0.9), rs7128071 (0.9), rs112704540 (0.89), rs61903418 (0.89), rs73008568 (0.88), rs17120280 (0.88), rs4938330 (0.88), rs7484045 (0.87), rs76136280 (0.87), rs61903419 (0.86), rs56134734 (0.85), rs688456 (0.85), rs61905680 (0.85), rs1473325 (0.84), rs1815789 (0.84), rs7946729 (0.83), rs7130531 (0.83), rs61907591 (0.83), rs12274388 (0.82), rs61905681 (0.82), rs61903427 (0.81), rs7946219 (0.81), rs78442878 (0.81), rs61624438 (0.8), rs12721030 (0.8), rs11216239 (0.79), rs7128772 (0.79), rs10892055 (0.79), rs723955 (0.78), rs6589580 (0.78), rs61903426 (0.77), rs59700451 (0.77), rs56371319 (0.77), rs59266239 (0.76), rs78857232 (0.75), rs7109649 (0.75), rs4938326 (0.75), rs56314469 (0.74), rs4938317 (0.74), rs7930264 (0.73), rs7112967 (0.73), rs144015791 (0.73), rs77953246 (0.72), rs61905689 (0.72), rs113560866 (0.71), rs1241655 (0.71), rs12721028 (0.71), rs56081989 (0.7), rs723953 (0.7),

rs11216266 (0.69), rs111901638 (0.69), rs61903394 (0.69), rs7931398 (0.68), rs113979111 (0.68), rs61905155 (0.67), rs11216255 (0.67), rs112151056 (0.67), rs77320801 (0.66), rs55703909 (0.66), rs79974099 (0.65), rs61903423 (0.65), rs59511712 (0.65), rs12099358 (0.64), rs61905678 (0.64), rs4938342 (0.63), rs74691084 (0.63), rs68137714 (0.63), rs7125788 (0.62), rs56227539 (0.62), rs4938321 (0.61), rs4388921 (0.61), rs11216153 (0.6), rs11216275 (0.6), rs1531706 (0.6), rs11823889 (0.59), rs721783 (0.59), rs10892054 (0.58), rs4938355 (0.58), rs112895963 (0.58), rs4938327 (0.57), rs61903395 (0.57), rs6589583 (0.56), rs17120251 (0.56), rs61905717 (0.56), rs4936361 (0.55), rs11216169 (0.55), rs1838757 (0.54), rs11826742 (0.54), rs983567 (0.54), rs10892066 (0.53), rs4548653 (0.53), rs7124996 (0.52), rs61905706 (0.52), rs76690043 (0.52), rs61905679 (0.51), rs7112111 (0.51), rs4936360 (0.5), rs7130213 (0.5), rs888246 (0.5), rs11216178 (0.49), rs2075547 (0.49), rs4938357 (0.48), rs61905716 (0.48), rs17120111 (0.48), rs11216187 (0.47), rs7109876 (0.47), rs61903429 (0.46), rs11216168 (0.46), rs61905677 (0.46), rs17120233 (0.45), rs3181 (0.45), rs7950093 (0.44), rs61907605 (0.44), rs11827828 (0.44), rs7950381 (0.43), rs11216245 (0.43), rs113784559 (0.42), rs4938341 (0.42), rs4936363 (0.42), rs4938333 (0.41), rs56016514 (0.41), rs1473326 (0.4), rs11216240 (0.4), rs7930783 (0.4), rs61905691 (0.39), rs7930409 (0.39), rs11826651 (0.38), rs7118591 (0.38), rs74341458 (0.38), rs7950213 (0.37), rs75904569 (0.37), rs670 (0.36), rs17120244 (0.36), rs4938329 (0.35), rs10082609 (0.35), rs61903398 (0.35), rs75920871 (0.34), rs4938336 (0.34), rs184090774 (0.33), rs75858236 (0.33), rs115787357 (0.33), rs2070666 (0.32), rs12279066 (0.32), rs117287138 (0.31), rs111327557 (0.31), rs10892042 (0.31), rs61903399 (0.3), rs12225230 (0.3), rs17120241 (0.29), rs76523703 (0.29), rs7124872 (0.29), rs4936359 (0.28), rs74328581 (0.28), rs10892044 (0.27), rs11823013 (0.27), rs7106662 (0.27), rs61905712 (0.26), rs7112191 (0.26), rs61903417 (0.25), rs6589595 (0.25), rs2513094 (0.25), rs7119185 (0.24), rs61905705 (0.24), rs682048 (0.23), rs7108912 (0.23), rs74428560 (0.23), rs10892071 (0.22), rs10790166 (0.22), rs625145 (0.21), rs3886960 (0.21), rs4938325 (0.21), rs2080586 (0.2), rs12271161 (0.2), rs4316540 (0.19), rs7950501 (0.19), rs76972960 (0.19), rs1940626 (0.18), rs11216241 (0.18), rs1473177 (0.17), rs2513097 (0.17), rs888245 (0.17), rs17120197 (0.16), rs61907594 (0.16), rs7924318 (0.15), rs7946399 (0.15), rs61905713 (0.15), rs113184611 (0.14), rs10466590 (0.14), rs61903416 (0.13), rs76868258 (0.13), rs7932655 (0.13), rs4936362 (0.12), rs1871756 (0.12), rs61905718 (0.11), rs7123336 (0.11), rs61905709 (0.1), rs7104023 (0.1), rs6589587 (0.1), rs7934763 (0.09), rs4938338 (0.09), rs59294170 (0.08), rs10892075 (0.08), rs45625638 (0.08), rs61903421 (0.07), rs61905719 (0.07), rs61903430 (0.06), rs11216180 (0.06), rs1473324 (0.06), rs61905145 (0.05), rs509171 (0.05), rs7946869 (0.04), rs7935834 (0.04), rs111560312 (0.04), rs4938339 (0.03), rs10892045 (0.03), rs12274465 (0.02), rs1815788 (0.02), rs79530380 (0.02), rs7101763 (0.01), rs11820814 (0.01), rs1145206 (0)

rs4969182	73	rs11653305 (1), rs17561950 (0.99), rs4969145 (0.97), rs3817294 (0.96), rs2376584 (0.95), rs4969142 (0.93), rs56658017 (0.92), rs11077364 (0.91), rs11077363 (0.89), rs12452576 (0.88), rs7208596 (0.86), rs12601079 (0.85), rs11656568 (0.84), rs6501208 (0.82), rs8075131 (0.81), rs12450500 (0.8), rs4580257 (0.78), rs8072632 (0.77), rs7212201 (0.76), rs55633207 (0.74), rs12948394 (0.73), rs4490057 (0.72), rs12450528 (0.7), rs11077361 (0.69), rs938351 (0.68), rs11077362 (0.66), rs3826323 (0.65), rs12602206 (0.64), rs2292643 (0.62), rs2292640 (0.61), rs4969187 (0.59), rs4969186 (0.58), rs9900564 (0.57), rs4969143 (0.55), rs12951460 (0.54), rs4969184 (0.53), rs4129767 (0.51), rs12451109 (0.49), rs55938653 (0.47), rs2292644 (0.46), rs4082919 (0.45), rs4969179 (0.43), rs7207782 (0.42), rs4366775 (0.41), rs8071889 (0.39), rs4969183 (0.38), rs11657865 (0.36), rs4969141 (0.35), rs12451715 (0.34), rs3744217 (0.32), rs12451791 (0.31), rs2292642 (0.3), rs7219625 (0.28), rs938350 (0.27), rs7225449 (0.26), rs16971107 (0.24), rs7215542 (0.23), rs62079153 (0.22), rs1976703 (0.2), rs938348 (0.19), rs12944051 (0.18), rs3817295 (0.16), rs16971105 (0.15), rs1976704 (0.14), rs11657987 (0.12), rs3744219 (0.11), rs11657860 (0.09), rs11653383 (0.08), rs2376583 (0.07), rs4969178 (0.05), rs8077859 (0.04), rs4969144 (0.03), rs12453598 (0.01)
rs56105022	62	rs12138866 (1), rs10799507 (0.98), rs75036533 (0.97), rs6694451 (0.95), rs12126815 (0.94), rs12140137 (0.92), rs10916458 (0.9), rs143868723 (0.89), rs6686449 (0.87), rs12128376 (0.86), rs72753956 (0.84), rs6701036 (0.83), rs72753994 (0.81), rs10799491 (0.79), rs10799543 (0.78), rs45583539 (0.76), rs60256604 (0.75), rs151143241 (0.73), rs10916498 (0.71), rs10916497 (0.7), rs10916481 (0.68), rs150967467 (0.67), rs112600662 (0.65), rs544428583 (0.63), rs150817716 (0.62), rs12133599 (0.6), rs12120308 (0.59), rs12083770 (0.57), rs12124547 (0.56), rs12125241 (0.54), rs12082619 (0.52), rs72753969 (0.51), rs76955951 (0.49), rs12184283 (0.48), rs139734197 (0.46), rs72751651 (0.44), rs10916414 (0.43), rs4463628 (0.41), rs12129540 (0.4), rs147479860 (0.38), rs573877473 (0.37), rs72746911 (0.35), rs7514140 (0.33), rs72753910 (0.32), rs12117152 (0.3), rs72753980 (0.29), rs72754000 (0.27), rs115310585 (0.25), rs72754001 (0.24), rs116590674 (0.22), rs141470848 (0.21), rs76962725 (0.19), rs12144137 (0.17), rs12117480 (0.16), rs7552239 (0.14), rs74750827 (0.13), rs12126765 (0.11), rs116232045 (0.1), rs72751628 (0.08), rs6660194 (0.06), rs10916462 (0.05), rs10916472 (0.03)
rs56122753	24	rs12502605 (1), rs1912897 (0.96), rs73243884 (0.92), rs6819735 (0.88), rs73243890 (0.84), rs2137234 (0.76), rs56147366 (0.72), rs10019888 (0.68), rs2137236 (0.64), rs1567651 (0.6), rs28673139 (0.56), rs2048508 (0.52), rs7698037 (0.48), rs1022186 (0.44), rs12513287 (0.4), rs4558863 (0.36), rs6448429 (0.32), rs2048507 (0.28), rs73243877 (0.24), rs17630194 (0.2), rs9884830 (0.16), rs12498488 (0.12), rs2137235 (0.08), rs6846421 (0.04)
rs56343119	27	rs635634 (1), rs11244061 (0.96), rs28602660 (0.93), rs9650778 (0.89), rs28602591 (0.86), rs28645493 (0.82), rs28538967 (0.79),

		rs17474001 (0.75), rs3124761 (0.71), rs532436 (0.68), rs28446901 (0.64), rs28658542 (0.61), rs507666 (0.57), rs115478735 (0.54), rs41302667 (0.5), rs41296107 (0.43), rs3758348 (0.39), rs41302673 (0.36), rs3124766 (0.32), rs2519093 (0.29), rs36222279 (0.25), rs4962153 (0.21), rs739468 (0.18), rs76238963 (0.14), rs3094379 (0.11), rs587600964 (0.07), rs3124764 (0.04)
rs6058035	325	rs6059846 (1), rs6088962 (1), rs58662941 (0.99), rs6121245 (0.99), rs6059841 (0.99), rs6059982 (0.98), rs2889528 (0.98), rs2811 (0.98), rs6058233 (0.98), rs6087701 (0.97), rs6088798 (0.97), rs6058148 (0.97), rs6120858 (0.96), rs6119539 (0.96), rs79072922 (0.95), rs6058450 (0.95), rs6120713 (0.95), rs6060203 (0.95), rs45557936 (0.94), rs6060912 (0.94), rs7270584 (0.94), rs6060262 (0.93), rs1994249 (0.93), rs6058261 (0.93), rs6120731 (0.92), rs6057911 (0.92), rs6059869 (0.92), rs6060403 (0.91), rs139441694 (0.91), rs6060174 (0.91), rs879597 (0.91), rs6058102 (0.9), rs6058116 (0.9), rs6060007 (0.9), rs6088749 (0.89), rs6120832 (0.89), rs2376992 (0.89), rs6120670 (0.88), rs6060142 (0.88), rs3181073 (0.88), rs6089055 (0.88), rs6119574 (0.87), rs79392518 (0.87), rs6579239 (0.87), rs2376864 (0.86), rs6058463 (0.86), rs6057998 (0.86), rs6088438 (0.85), rs6058122 (0.85), rs6119457 (0.85), rs6120609 (0.84), rs6060644 (0.84), rs6060295 (0.84), rs75950492 (0.84), rs6058023 (0.83), rs6060101 (0.83), rs1923026 (0.83), rs6060134 (0.82), rs8123569 (0.82), rs6060633 (0.82), rs6060821 (0.81), rs6087711 (0.81), rs6060923 (0.81), rs6060195 (0.81), rs6089093 (0.8), rs6119651 (0.8), rs6088462 (0.8), rs6120950 (0.79), rs115585940 (0.79), rs6121221 (0.79), rs6119729 (0.78), rs6060087 (0.78), rs6088796 (0.78), rs6060381 (0.78), rs6120970 (0.77), rs6058197 (0.77), rs57574578 (0.77), rs6088736 (0.76), rs6060344 (0.76), rs6060263 (0.76), rs58875461 (0.75), rs6058189 (0.75), rs6059727 (0.75), rs113400846 (0.74), rs7361893 (0.74), rs6060960 (0.74), rs6060323 (0.74), rs6057912 (0.73), rs6059952 (0.73), rs7271374 (0.73), rs6058086 (0.72), rs6060987 (0.72), rs7265413 (0.72), rs113933395 (0.71), rs6088858 (0.71), rs6060937 (0.71), rs6060066 (0.71), rs1075698 (0.7), rs2064818 (0.7), rs6120815 (0.7), rs6058004 (0.69), rs6121209 (0.69), rs6059696 (0.69), rs6060289 (0.68), rs7274269 (0.68), rs6059889 (0.68), rs6120607 (0.67), rs6120890 (0.67), rs1471782 (0.67), rs7266471 (0.67), rs73241710 (0.66), rs8120526 (0.66), rs6059981 (0.66), rs6060474 (0.65), rs6057968 (0.65), rs6058151 (0.65), rs6060929 (0.64), rs6060145 (0.64), rs6120719 (0.64), rs6058298 (0.64), rs6120618 (0.63), rs6060359 (0.63), rs6058141 (0.63), rs6058187 (0.62), rs144791144 (0.62), rs6060247 (0.62), rs2016742 (0.61), rs6058085 (0.61), rs1406920 (0.61), rs6059958 (0.6), rs80080420 (0.6), rs113103684 (0.6), rs6058088 (0.6), rs6059979 (0.59), rs6060291 (0.59), rs6089094 (0.59), rs6060114 (0.58), rs6088998 (0.58), rs6088857 (0.58), rs6119718 (0.57), rs6059806 (0.57), rs6088434 (0.57), rs6059983 (0.57), rs6088864 (0.56), rs6059894 (0.56), rs6059690 (0.56), rs6059930 (0.55), rs7266148 (0.55), rs6119610 (0.55), rs6088855 (0.54), rs6058110 (0.54), rs6059796 (0.54), rs368329232 (0.53), rs8121699 (0.53), rs6060075

		(0.53), rs6058140 (0.53), rs6059929 (0.52), rs7269866 (0.52), rs6060395 (0.52), rs6059795 (0.51), rs6059984 (0.51), rs6120868 (0.51), rs6088446 (0.5), rs2376989 (0.5), rs6059874 (0.5), rs1115713 (0.49), rs6060260 (0.49), rs7271671 (0.49), rs6060812 (0.48), rs6060089 (0.48), rs6059704 (0.48), rs6060297 (0.47), rs112200607 (0.47), rs752841 (0.47), rs6058058 (0.47), rs6121242 (0.46), rs6058033 (0.46), rs15817 (0.46), rs6058172 (0.45), rs2376996 (0.45), rs6088856 (0.45), rs6058087 (0.44), rs8126422 (0.44), rs6058131 (0.44), rs6088793 (0.43), rs1994250 (0.43), rs6060425 (0.43), rs6120862 (0.43), rs142923745 (0.42), rs6060249 (0.42), rs113882388 (0.41), rs7272062 (0.41), rs6119720 (0.41), rs2038397 (0.4), rs7346070 (0.4), rs6059906 (0.4), rs56090668 (0.4), rs7261354 (0.39), rs6060992 (0.39), rs6120704 (0.39), rs6087716 (0.38), rs6058448 (0.38), rs6059980 (0.38), rs73235714 (0.37), rs6089058 (0.37), rs2376863 (0.37), rs6120451 (0.36), rs6060793 (0.36), rs6058152 (0.36), rs6060979 (0.36), rs6119725 (0.35), rs2376990 (0.35), rs6060399 (0.35), rs6088790 (0.34), rs73235789 (0.34), rs6060211 (0.34), rs6060989 (0.33), rs61676755 (0.33), rs3932482 (0.33), rs2889487 (0.33), rs1994251 (0.32), rs6119472 (0.32), rs6060171 (0.32), rs4607 (0.31), rs77130187 (0.31), rs6089050 (0.31), rs6059761 (0.3), rs7362275 (0.3), rs6059830 (0.3), rs57726761 (0.29), rs6088374 (0.29), rs11906851 (0.29), rs6120937 (0.29), rs6087687 (0.28), rs6087778 (0.28), rs6058421 (0.28), rs6088806 (0.27), rs6088451 (0.27), rs6058022 (0.27), rs3203770 (0.26), rs6058103 (0.26), rs717064 (0.26), rs6060210 (0.26), rs6060763 (0.25), rs6060446 (0.25), rs6060144 (0.25), rs6120898 (0.24), rs6089070 (0.24), rs6121246 (0.24), rs6119442 (0.23), rs6088592 (0.23), rs6088669 (0.23), rs750144 (0.22), rs6119575 (0.22), rs6060409 (0.22), rs6121016 (0.21), rs78881751 (0.21), rs6120671 (0.21), rs2376987 (0.2), rs6060411 (0.2), rs112367115 (0.2), rs6059781 (0.19), rs6060870 (0.19), rs6059888 (0.19), rs6089071 (0.19), rs6060252 (0.18), rs6120835 (0.18), rs6059948 (0.18), rs6058259 (0.17), rs6120619 (0.17), rs6059899 (0.17), rs6058203 (0.16), rs6119449 (0.16), rs2889488 (0.16), rs6120905 (0.16), rs6060076 (0.15), rs6059702 (0.15), rs6120700 (0.15), rs6059873 (0.14), rs7263175 (0.14), rs6060002 (0.14), rs6088853 (0.13), rs6119489 (0.13), rs8126355 (0.13), rs11905777 (0.12), rs6059634 (0.12), rs113818546 (0.12), rs6058460 (0.12), rs6059870 (0.11), rs6579238 (0.11), rs113502509 (0.11), rs7273756 (0.1), rs186816175 (0.1), rs2839862 (0.1), rs6060198 (0.09), rs74392247 (0.09), rs6088814 (0.09), rs6060978 (0.09), rs7354225 (0.08), rs6120452 (0.08), rs6087710 (0.08), rs6058196 (0.07), rs6087700 (0.07), rs6060086 (0.07), rs6088854 (0.06), rs6059713 (0.06), rs6120879 (0.06), rs6058069 (0.05), rs6058470 (0.05), rs6060679 (0.05), rs6060296 (0.05), rs4518038 (0.04), rs6060286 (0.04), rs6060116 (0.04), rs138322105 (0.03), rs6060118 (0.03), rs2233834 (0.03), rs6058171 (0.02), rs2376862 (0.02), rs112805309 (0.02), rs17093657 (0.02), rs6060832 (0.01), rs58757492 (0.01), rs6060261 (0.01), rs56153385 (0)
rs62036658	327	rs181206 (1), rs181204 (1), rs62034324 (0.99), rs28410083 (0.99), rs12446589 (0.99), rs1690405 (0.98), rs62037365 (0.98),

rs4788080 (0.98), rs11646653 (0.98), rs4788078 (0.97), rs151303 (0.97), rs4788062 (0.97), rs34835 (0.96), rs12443881 (0.96), rs62036626 (0.95), rs6565229 (0.95), rs7191618 (0.95), rs151181 (0.95), rs550572318 (0.94), rs62037369 (0.94), rs7499337 (0.94), rs41278162 (0.93), rs3927084 (0.93), rs62032560 (0.93), rs35175818 (0.92), rs4788083 (0.92), rs7359397 (0.92), rs55719896 (0.92), rs8046545 (0.91), rs151174 (0.91), rs62036622 (0.91), rs4788073 (0.9), rs62032566 (0.9), rs1987472 (0.9), rs7193402 (0.89), rs62036614 (0.89), rs35626515 (0.89), rs9708958 (0.88), rs55991577 (0.88), rs149601295 (0.88), rs7206214 (0.87), rs9972693 (0.87), rs112047757 (0.87), rs4788100 (0.86), rs762634 (0.86), rs4149390 (0.86), rs6565259 (0.85), rs3020803 (0.85), rs56272201 (0.85), rs74752114 (0.85), rs153109 (0.84), rs7185232 (0.84), rs62034325 (0.84), rs8056890 (0.83), rs151179 (0.83), rs149299 (0.83), rs7500321 (0.82), rs4149406 (0.82), rs4788102 (0.82), rs56040780 (0.82), rs7188071 (0.81), rs62036621 (0.81), rs371918919 (0.81), rs151232 (0.8), rs12447461 (0.8), rs62031607 (0.8), rs4788099 (0.79), rs4788074 (0.79), rs7187575 (0.79), rs151301 (0.79), rs180743 (0.78), rs11642449 (0.78), rs371723244 (0.78), rs40837 (0.77), rs4149391 (0.77), rs2008514 (0.77), rs26528 (0.76), rs62037414 (0.76), rs111384198 (0.76), rs2106480 (0.76), rs1059491 (0.75), rs11861132 (0.75), rs4149392 (0.75), rs2077031 (0.74), rs151321 (0.74), rs4788064 (0.74), rs62036624 (0.73), rs11645306 (0.73), rs8049739 (0.73), rs7192601 (0.73), rs865719 (0.72), rs11864750 (0.72), rs8055982 (0.72), rs180744 (0.71), rs56186137 (0.71), rs11641216 (0.71), rs7498555 (0.7), rs56209193 (0.7), rs11864107 (0.7), rs62034323 (0.69), rs62037366 (0.69), rs11639897 (0.69), rs181205 (0.69), rs11074907 (0.68), rs62031458 (0.68), rs240702 (0.68), rs9282864 (0.67), rs138592330 (0.67), rs78613234 (0.67), rs55792032 (0.66), rs3785354 (0.66), rs4451951 (0.66), rs4788101 (0.66), rs202210003 (0.65), rs4787456 (0.65), rs6565293 (0.65), rs9931989 (0.64), rs7184597 (0.64), rs1126446 (0.64), rs12448902 (0.63), rs1690407 (0.63), rs62031560 (0.63), rs4788084 (0.63), rs7202948 (0.62), rs80275162 (0.62), rs3924376 (0.62), rs2650495 (0.61), rs62036620 (0.61), rs6839 (0.61), rs149278 (0.6), rs4115668 (0.6), rs72793811 (0.6), rs62034351 (0.6), rs181203 (0.59), rs62034355 (0.59), rs28676837 (0.59), rs7193733 (0.58), rs4788070 (0.58), rs56396270 (0.58), rs149271 (0.57), rs181207 (0.57), rs8060365 (0.57), rs12445744 (0.56), rs7198606 (0.56), rs116840534 (0.56), rs563080102 (0.56), rs62037363 (0.55), rs2411453 (0.55), rs181209 (0.55), rs12444171 (0.54), rs2925630 (0.54), rs56091264 (0.54), rs62031562 (0.53), rs62034321 (0.53), rs62037367 (0.53), rs4787457 (0.53), rs75539558 (0.52), rs7498603 (0.52), rs62034358 (0.52), rs12919058 (0.51), rs4787458 (0.51), rs3020805 (0.51), rs240705 (0.5), rs62034350 (0.5), rs62034322 (0.5), rs240704 (0.5), rs7191548 (0.49), rs11859512 (0.49), rs40831 (0.49), rs750155 (0.48), rs411518 (0.48), rs62034319 (0.48), rs2925629 (0.47), rs378421 (0.47), rs1968751 (0.47), rs189278832 (0.47), rs2925628 (0.46), rs56169261 (0.46), rs6498089 (0.46), rs1042157 (0.45), rs12445823 (0.45), rs74459546 (0.45), rs27741 (0.44), rs112191041 (0.44), rs147684209 (0.44), rs1074631

		(0.44), rs62036618 (0.43), rs2411440 (0.43), rs62034318 (0.43), rs7187776 (0.42), rs7186573 (0.42), rs56354901 (0.42), rs201918842 (0.41), rs56358680 (0.41), rs4788076 (0.41), rs240707 (0.4), rs35280834 (0.4), rs4787455 (0.4), rs12448429 (0.4), rs7187604 (0.39), rs11150609 (0.39), rs4149393 (0.39), rs111693583 (0.38), rs4149389 (0.38), rs8045689 (0.38), rs3020806 (0.37), rs114558014 (0.37), rs56305122 (0.37), rs151182 (0.37), rs113029997 (0.36), rs112366001 (0.36), rs28772958 (0.36), rs79533802 (0.35), rs55830740 (0.35), rs62034359 (0.35), rs10499 (0.34), rs4788085 (0.34), rs394502 (0.34), rs4149394 (0.34), rs9926245 (0.33), rs12446550 (0.33), rs743590 (0.33), rs545645107 (0.32), rs75301646 (0.32), rs12325113 (0.32), rs199943670 (0.31), rs4788069 (0.31), rs4072402 (0.31), rs2925624 (0.31), rs56226746 (0.3), rs181195 (0.3), rs153106 (0.3), rs42861 (0.29), rs11860513 (0.29), rs7190771 (0.29), rs11074911 (0.28), rs2650496 (0.28), rs710410 (0.28), rs115201991 (0.27), rs17707300 (0.27), rs3888190 (0.27), rs2904880 (0.27), rs62034326 (0.26), rs34839 (0.26), rs56163509 (0.26), rs7498665 (0.25), rs1126447 (0.25), rs545943172 (0.25), rs12325014 (0.24), rs41278158 (0.24), rs79039694 (0.24), rs4788095 (0.24), rs4149388 (0.23), rs11861174 (0.23), rs35184893 (0.23), rs62036613 (0.22), rs62036657 (0.22), rs4788081 (0.22), rs1968752 (0.21), rs151178 (0.21), rs112831778 (0.21), rs72793815 (0.21), rs72793818 (0.2), rs28480369 (0.2), rs28403629 (0.2), rs762633 (0.19), rs113079736 (0.19), rs8062405 (0.19), rs72793812 (0.18), rs28449958 (0.18), rs28729187 (0.18), rs62037371 (0.18), rs13331170 (0.17), rs34954534 (0.17), rs2925623 (0.17), rs8050972 (0.16), rs376631209 (0.16), rs11863370 (0.16), rs7189927 (0.15), rs3088215 (0.15), rs8061590 (0.15), rs202014106 (0.15), rs62037364 (0.14), rs9282862 (0.14), rs111292564 (0.14), rs61737565 (0.13), rs34838 (0.13), rs62036616 (0.13), rs4788079 (0.12), rs3859172 (0.12), rs7205323 (0.12), rs12325278 (0.11), rs9972768 (0.11), rs111890869 (0.11), rs6498091 (0.11), rs40836 (0.1), rs187668816 (0.1), rs62036617 (0.1), rs12928404 (0.09), rs72793809 (0.09), rs3020804 (0.09), rs4788077 (0.08), rs7187333 (0.08), rs8049439 (0.08), rs56404918 (0.08), rs113208333 (0.07), rs149301 (0.07), rs79046494 (0.06), rs62036623 (0.06), rs240708 (0.06), rs4149398 (0.05), rs8055138 (0.05), rs4788068 (0.05), rs56211668 (0.05), rs8046271 (0.04), rs1136703 (0.04), rs151226 (0.04), rs437564 (0.03), rs1987471 (0.03), rs34836 (0.03), rs11643297 (0.02), rs28698667 (0.02), rs17640009 (0.02), rs40834 (0.01), rs11645143 (0.01), rs151180 (0.01), rs527435679 (0)
rs62396278	65	rs34972021 (1), rs114712755 (0.98), rs2894211 (0.97), rs1265096 (0.95), rs4997235 (0.94), rs12196740 (0.92), rs35990442 (0.91), rs10947210 (0.89), rs115959678 (0.88), rs62396277 (0.86), rs41541519 (0.85), rs374842482 (0.83), rs7767033 (0.82), rs62395422 (0.8), rs62398265 (0.79), rs4997234 (0.77), rs62396280 (0.76), rs12660314 (0.74), rs56189237 (0.73), rs4993823 (0.71), rs2394987 (0.7), rs62395278 (0.68), rs35192021 (0.67), rs28428768 (0.65), rs182914108 (0.64), rs62398260 (0.62), rs116010992 (0.61), rs7767328 (0.59), rs62395441 (0.58), rs6905742 (0.56), rs41551018 (0.55), rs62395421 (0.53), rs62396276

		(0.52), rs6918524 (0.5), rs62395440 (0.48), rs116812356 (0.47), rs11962586 (0.45), rs6901719 (0.44), rs114443705 (0.42), rs1265075 (0.41), rs34507986 (0.39), rs41558815 (0.38), rs35840219 (0.36), rs12665803 (0.35), rs62395274 (0.33), rs116157258 (0.32), rs114777460 (0.3), rs62395458 (0.29), rs9266301 (0.27), rs62395307 (0.26), rs34277309 (0.24), rs7767632 (0.23), rs9266834 (0.21), rs140949683 (0.2), rs62395275 (0.18), rs12660817 (0.17), rs7767365 (0.15), rs116382538 (0.14), rs62395459 (0.12), rs4327724 (0.11), rs4122185 (0.09), rs7751194 (0.08), rs373551315 (0.06), rs41563615 (0.05), rs35193884 (0.03)
rs6420185	30	rs35664331 (1), rs11136272 (0.97), rs10087900 (0.94), rs62523440 (0.9), rs67149914 (0.87), rs35297056 (0.84), rs7825338 (0.81), rs10099293 (0.77), rs10216471 (0.74), rs62523434 (0.71), rs12676568 (0.68), rs61059595 (0.65), rs9650459 (0.61), rs9650460 (0.58), rs34039115 (0.55), rs66854753 (0.52), rs4074902 (0.48), rs34407803 (0.45), rs13261822 (0.42), rs4076111 (0.39), rs11538388 (0.35), rs11136271 (0.32), rs4075205 (0.29), rs56960668 (0.26), rs7388248 (0.23), rs11136269 (0.19), rs7460860 (0.13), rs72614045 (0.1), rs7822123 (0.06), rs6989167 (0.03)
rs650194	53	rs9424956 (1), rs687257 (0.98), rs500647 (0.96), rs2300161 (0.94), rs11587601 (0.93), rs486625 (0.89), rs625617 (0.87), rs6670616 (0.85), rs11209722 (0.83), rs12067177 (0.81), rs657262 (0.8), rs583691 (0.78), rs17090710 (0.76), rs12097924 (0.74), rs2300164 (0.72), rs12067214 (0.7), rs9970739 (0.69), rs2421735 (0.67), rs1071020 (0.65), rs36075725 (0.63), rs11209730 (0.61), rs12060001 (0.59), rs515036 (0.57), rs499641 (0.56), rs657169 (0.54), rs926457 (0.52), rs509789 (0.5), rs17090700 (0.48), rs2072947 (0.46), rs9424958 (0.44), rs2268057 (0.43), rs3819783 (0.41), rs12066813 (0.39), rs34285961 (0.37), rs661000 (0.35), rs594095 (0.33), rs3819790 (0.31), rs598566 (0.3), rs12407552 (0.28), rs7541717 (0.26), rs624709 (0.24), rs9424957 (0.22), rs5697 (0.2), rs2072946 (0.19), rs7516479 (0.17), rs553067 (0.15), rs9425049 (0.13), rs13374231 (0.11), rs11576593 (0.09), rs594454 (0.07), rs681196 (0.06), rs480867 (0.04), rs521870 (0.02)
rs66906321	208	rs6755502 (1), rs7558910 (1), rs5017301 (0.99), rs7585056 (0.99), rs12714414 (0.98), rs4854343 (0.98), rs2867108 (0.97), rs4854340 (0.97), rs12997450 (0.96), rs10865550 (0.96), rs4854342 (0.95), rs2867105 (0.95), rs5017303 (0.94), rs13397165 (0.94), rs5017304 (0.93), rs1320336 (0.93), rs6732341 (0.92), rs13401686 (0.92), rs2947415 (0.91), rs13396119 (0.91), rs13035713 (0.91), rs7570993 (0.9), rs35802221 (0.9), rs7570198 (0.89), rs6548240 (0.89), rs10193244 (0.88), rs1320330 (0.88), rs7604609 (0.87), rs13008590 (0.87), rs6731225 (0.86), rs13028310 (0.86), rs939582 (0.85), rs6731348 (0.85), rs4854348 (0.84), rs13007086 (0.84), rs11127491 (0.83), rs13396935 (0.83), rs13412197 (0.82), rs6749087 (0.82), rs13388043 (0.82), rs62105304 (0.81), rs12993295 (0.81), rs11687052 (0.8), rs2867111 (0.8), rs2867109 (0.79), rs7574359 (0.79), rs7567570 (0.78), rs7576624 (0.78), rs6734363 (0.77), rs34119520 (0.77), rs7571957 (0.76), rs62105307 (0.76), rs2867110 (0.75), rs13007080 (0.75),

		rs7563362 (0.74), rs6735024 (0.74), rs4429506 (0.73), rs4854345 (0.73), rs4613321 (0.73), rs115910474 (0.72), rs13021737 (0.72), rs5017302 (0.71), rs6719883 (0.71), rs13028737 (0.7), rs4854349 (0.7), rs7564711 (0.69), rs2867124 (0.69), rs7606023 (0.68), rs7567710 (0.68), rs6748821 (0.67), rs6732471 (0.67), rs11127488 (0.66), rs62650890 (0.66), rs6752470 (0.65), rs12992154 (0.65), rs6751993 (0.64), rs6717005 (0.64), rs10166979 (0.64), rs2903492 (0.63), rs6746275 (0.63), rs12995480 (0.62), rs35913461 (0.62), rs13387090 (0.61), rs13387091 (0.61), rs10865551 (0.6), rs6731688 (0.6), rs2867131 (0.59), rs6548237 (0.59), rs62105303 (0.58), rs7594220 (0.58), rs10189761 (0.57), rs13386517 (0.57), rs7604752 (0.56), rs6711254 (0.56), rs10169736 (0.55), rs6749375 (0.55), rs2867123 (0.55), rs11127485 (0.54), rs6756518 (0.54), rs11127486 (0.53), rs13412194 (0.53), rs62105327 (0.52), rs7576635 (0.52), rs7560127 (0.51), rs6725549 (0.51), rs11127483 (0.5), rs35628463 (0.5), rs1320338 (0.49), rs6752706 (0.49), rs2867114 (0.48), rs6548239 (0.48), rs11127484 (0.47), rs6719980 (0.47), rs7601028 (0.46), rs7561556 (0.46), rs35796073 (0.45), rs6711012 (0.45), rs6744646 (0.45), rs13393304 (0.44), rs7569210 (0.44), rs13386964 (0.43), rs6751994 (0.43), rs6725686 (0.42), rs4854339 (0.42), rs6743060 (0.41), rs4854347 (0.41), rs13012571 (0.4), rs13027828 (0.4), rs13013021 (0.39), rs2867125 (0.39), rs7608050 (0.38), rs11127487 (0.38), rs5017300 (0.37), rs6731872 (0.37), rs374892365 (0.36), rs10172769 (0.36), rs12995666 (0.36), rs35142762 (0.35), rs10865548 (0.35), rs5017305 (0.34), rs7570232 (0.34), rs4854346 (0.33), rs2867113 (0.33), rs2860323 (0.32), rs13391851 (0.32), rs13028252 (0.31), rs7569102 (0.31), rs939583 (0.3), rs62105306 (0.3), rs1320332 (0.29), rs7564708 (0.29), rs13387939 (0.28), rs7567368 (0.28), rs7559547 (0.27), rs2903489 (0.27), rs10865549 (0.27), rs13022164 (0.26), rs74676797 (0.26), rs7560246 (0.25), rs4854344 (0.25), rs13386627 (0.24), rs2867112 (0.24), rs11127489 (0.23), rs11127490 (0.23), rs6739303 (0.22), rs13025697 (0.21), rs6548238 (0.21), rs2867115 (0.2), rs7588007 (0.2), rs73139123 (0.19), rs7571970 (0.19), rs7571967 (0.18), rs4452188 (0.18), rs12476198 (0.18), rs1320331 (0.17), rs10192350 (0.17), rs13415094 (0.16), rs7559018 (0.16), rs2903490 (0.15), rs12463617 (0.15), rs4423631 (0.14), rs6760543 (0.13), rs13405307 (0.13), rs10173167 (0.12), rs7579427 (0.12), rs7591067 (0.11), rs13022337 (0.1), rs6735049 (0.1), rs1320337 (0.09), rs6711039 (0.09), rs4407278 (0.09), rs6744653 (0.08), rs7561317 (0.08), rs939581 (0.07), rs6728726 (0.07), rs6760274 (0.06), rs7579971 (0.06), rs2867122 (0.05), rs939584 (0.05), rs10190052 (0.04), rs12714415 (0.04), rs12714413 (0.03), rs12623218 (0.03), rs7559447 (0.02), rs2947411 (0.02), rs10190313 (0.01), rs6719518 (0.01), rs12992672 (0)
rs6724214	123	rs73937421 (1), rs13417051 (0.99), rs57552961 (0.98), rs13395751 (0.98), rs6715161 (0.97), rs9678301 (0.96), rs75123794 (0.95), rs13387291 (0.94), rs7588134 (0.94), rs78472090 (0.93), rs60776154 (0.92), rs72906786 (0.91), rs6759437 (0.9), rs6745729 (0.9), rs6545372 (0.89), rs11889527 (0.88), rs13432387 (0.87), rs75237140 (0.86), rs7583508 (0.86), rs76073574

		(0.85), rs72906704 (0.84), rs6545382 (0.83), rs78542544 (0.82), rs1049894 (0.82), rs11884857 (0.81), rs6545368 (0.8), rs7556936 (0.79), rs7599335 (0.78), rs76039968 (0.78), rs11890472 (0.77), rs28699646 (0.76), rs6731911 (0.75), rs111363964 (0.74), rs114242964 (0.74), rs6748686 (0.73), rs28571698 (0.72), rs10188834 (0.71), rs78887562 (0.7), rs13401719 (0.7), rs2112120 (0.69), rs10181332 (0.68), rs7559651 (0.67), rs10199995 (0.66), rs2177791 (0.66), rs143867525 (0.65), rs7560575 (0.64), rs112085448 (0.63), rs12613353 (0.62), rs7594327 (0.62), rs10208649 (0.6), rs724387 (0.59), rs112162975 (0.58), rs6712561 (0.58), rs58920915 (0.57), rs11886024 (0.56), rs77308271 (0.55), rs75213010 (0.54), rs116089439 (0.54), rs6545367 (0.53), rs2287348 (0.52), rs13389673 (0.51), rs72904915 (0.5), rs11125517 (0.5), rs11891000 (0.49), rs560447846 (0.48), rs75447473 (0.47), rs114837028 (0.46), rs6756424 (0.46), rs6739630 (0.45), rs41536750 (0.44), rs74627832 (0.43), rs542616714 (0.42), rs529220431 (0.42), rs6760038 (0.41), rs13408425 (0.4), rs11887593 (0.39), rs139089553 (0.38), rs6731217 (0.38), rs6730494 (0.37), rs72906778 (0.36), rs76948694 (0.35), rs77805752 (0.34), rs2177792 (0.34), rs10180492 (0.33), rs115322634 (0.32), rs7560832 (0.31), rs10209918 (0.3), rs6761648 (0.3), rs11894028 (0.29), rs13387890 (0.28), rs72902931 (0.27), rs11904365 (0.26), rs2161083 (0.26), rs6719990 (0.25), rs78244745 (0.24), rs6751340 (0.23), rs10205469 (0.22), rs6723722 (0.22), rs138495949 (0.21), rs60769690 (0.2), rs13402778 (0.19), rs17045242 (0.18), rs7588319 (0.18), rs11886366 (0.17), rs6545369 (0.16), rs10190353 (0.15), rs7608085 (0.14), rs7608989 (0.14), rs10193846 (0.13), rs10199444 (0.12), rs6740063 (0.11), rs6545371 (0.1), rs3930909 (0.1), rs7565152 (0.09), rs2287347 (0.08), rs78688150 (0.07), rs74930210 (0.06), rs9309251 (0.06), rs13401491 (0.05), rs72906711 (0.04), rs59172147 (0.03), rs13382257 (0.02), rs72906714 (0.01)
rs686030	24	rs570524 (1), rs535772 (0.96), rs3933785 (0.92), rs675849 (0.88), rs540885 (0.84), rs1215112 (0.8), rs2156110 (0.76), rs3933784 (0.72), rs2616751 (0.68), rs2156109 (0.64), rs471364 (0.6), rs581080 (0.56), rs677622 (0.52), rs677578 (0.48), rs2185938 (0.44), rs1475229 (0.4), rs661048 (0.36), rs585002 (0.28), rs3905247 (0.24), rs578194 (0.2), rs4237138 (0.16), rs660686 (0.12), rs643531 (0.08), rs486349 (0.04)
rs7098444	184	rs2664282 (1), rs13013 (0.99), rs12783161 (0.99), rs2675680 (0.98), rs3957126 (0.97), rs2127365 (0.97), rs2633316 (0.96), rs150352103 (0.96), rs2227551 (0.95), rs2688610 (0.95), rs11000760 (0.94), rs2227560 (0.94), rs2688622 (0.93), rs111786550 (0.92), rs2227552 (0.92), rs2306328 (0.91), rs10762559 (0.91), rs2242256 (0.9), rs10762560 (0.9), rs2675677 (0.89), rs11000758 (0.89), rs12262650 (0.88), rs9971040 (0.88), rs55756042 (0.87), rs2242257 (0.87), rs2688611 (0.86), rs2688621 (0.85), rs2633299 (0.85), rs2242258 (0.84), rs2688607 (0.84), rs3861036 (0.83), rs2688623 (0.83), rs2633303 (0.82), rs2675670 (0.82), rs12253429 (0.81), rs28718766 (0.81), rs2633308 (0.8), rs11814282 (0.8), rs3812637 (0.79), rs7080579 (0.78), rs10733894 (0.78), rs4746143

		(0.77), rs7909544 (0.77), rs61866762 (0.76), rs2243610 (0.76), rs3849969 (0.75), rs1134777 (0.75), rs2633321 (0.74), rs2633322 (0.74), rs2688626 (0.73), rs2664284 (0.73), rs2633323 (0.72), rs2227571 (0.72), rs2227578 (0.71), rs2675668 (0.7), rs10824042 (0.7), rs2633309 (0.69), rs2894040 (0.69), rs72814394 (0.68), rs3870402 (0.68), rs11000745 (0.67), rs7898436 (0.67), rs35430208 (0.66), rs7908825 (0.66), rs2633313 (0.65), rs7923045 (0.65), rs2688620 (0.64), rs28553894 (0.63), rs10128302 (0.63), rs10824043 (0.62), rs2227576 (0.62), rs2633310 (0.61), rs3849967 (0.61), rs2675662 (0.6), rs11000804 (0.6), rs3870406 (0.59), rs11595817 (0.59), rs7903666 (0.58), rs9664186 (0.58), rs2675671 (0.57), rs11000744 (0.56), rs9664184 (0.56), rs2459449 (0.55), rs10824032 (0.55), rs10824041 (0.54), rs2459446 (0.54), rs2250140 (0.53), rs2688613 (0.52), rs2271271 (0.52), rs28736827 (0.51), rs2461864 (0.51), rs11000755 (0.5), rs12264025 (0.49), rs9971038 (0.49), rs2633304 (0.48), rs2461867 (0.48), rs3849968 (0.47), rs10128354 (0.47), rs7076525 (0.46), rs2675667 (0.46), rs4065 (0.45), rs2675663 (0.45), rs2675676 (0.44), rs2675679 (0.44), rs2227566 (0.43), rs3911887 (0.42), rs6480709 (0.42), rs11000746 (0.41), rs2633307 (0.41), rs2633300 (0.4), rs2675666 (0.4), rs2675669 (0.39), rs2688609 (0.39), rs11000743 (0.38), rs1815076 (0.38), rs2633319 (0.37), rs2227558 (0.37), rs11000767 (0.36), rs11591708 (0.35), rs7080350 (0.35), rs10128205 (0.34), rs10824031 (0.34), rs11000787 (0.33), rs7072988 (0.33), rs2688614 (0.32), rs2948689 (0.32), rs2633311 (0.31), rs10824040 (0.31), rs2688615 (0.3), rs2633314 (0.3), rs1916341 (0.29), rs9733806 (0.28), rs2688625 (0.28), rs2688617 (0.27), rs2633302 (0.27), rs2664280 (0.26), rs2633312 (0.26), rs12253408 (0.25), rs4746148 (0.25), rs7098573 (0.24), rs3933085 (0.24), rs10740417 (0.23), rs11000765 (0.23), rs7081276 (0.22), rs10128262 (0.22), rs10824036 (0.21), rs1135745 (0.2), rs71507081 (0.2), rs11000759 (0.19), rs10824028 (0.19), rs2688608 (0.18), rs7908630 (0.18), rs12775558 (0.17), rs3870401 (0.17), rs28417886 (0.16), rs4691 (0.16), rs11000785 (0.15), rs2633320 (0.15), rs10740418 (0.14), rs2675674 (0.13), rs10824044 (0.13), rs7909802 (0.12), rs2688616 (0.12), rs10762555 (0.11), rs2461863 (0.11), rs2675675 (0.1), rs2688612 (0.1), rs12247028 (0.09), rs28447450 (0.09), rs12413039 (0.08), rs2633317 (0.08), rs12766737 (0.07), rs12262649 (0.06), rs2633305 (0.06), rs10762557 (0.05), rs2688619 (0.05), rs10762563 (0.04), rs12253482 (0.04), rs7076585 (0.03), rs10762556 (0.03), rs2227575 (0.02), rs11000757 (0.02), rs2633318 (0.01), rs12256103 (0.01)
rs7213935	46	rs4793017 (1), rs7220711 (0.98), rs7209826 (0.96), rs4793022 (0.94), rs9902329 (0.91), rs6503470 (0.89), rs1983490 (0.87), rs2342315 (0.85), rs12600549 (0.83), rs4793018 (0.81), rs1877632 (0.79), rs8072333 (0.77), rs12602349 (0.74), rs12602890 (0.72), rs8072602 (0.7), rs2342312 (0.68), rs8079336 (0.66), rs1107747 (0.64), rs9908642 (0.62), rs8068071 (0.6), rs55921171 (0.57), rs2342314 (0.55), rs4793021 (0.53), rs4793016 (0.51), rs4793020 (0.49), rs9896582 (0.45), rs2342313 (0.43), rs1107748 (0.4), rs1828720 (0.38), rs8079873 (0.36), rs6503468 (0.34), rs4792907 (0.32), rs4792909 (0.3), rs4793023 (0.28), rs4792908

		(0.26), rs9911277 (0.23), rs9909172 (0.21), rs4793019 (0.19), rs9910625 (0.17), rs9897313 (0.15), rs4513138 (0.13), rs7217502 (0.11), rs59303349 (0.09), rs12603100 (0.06), rs1877633 (0.04), rs1881107 (0.02)
rs7254731	16	rs7253809 (1), rs77520801 (0.94), rs78443328 (0.82), rs7255723 (0.76), rs9749589 (0.71), rs60649443 (0.65), rs78918007 (0.59), rs34857663 (0.53), rs4353573 (0.47), rs28626338 (0.41), rs62128055 (0.35), rs8111953 (0.29), rs9749590 (0.24), rs9749585 (0.18), rs4806928 (0.12), rs192265830 (0.06)
rs72665235	10	rs201407410 (1), rs200247105 (0.91), rs142483715 (0.82), rs61779763 (0.73), rs372151476 (0.64), rs142358687 (0.55), rs115874855 (0.45), rs477913 (0.36), rs145830471 (0.27), rs72665233 (0.18)
rs7268701	307	rs11700221 (1), rs2144717 (1), rs4911155 (0.99), rs6087606 (0.99), rs6141465 (0.99), rs6059893 (0.98), rs6059845 (0.98), rs1884669 (0.98), rs9789792 (0.97), rs6088559 (0.97), rs6059956 (0.97), rs1018503 (0.96), rs4911158 (0.96), rs1205345 (0.96), rs4911151 (0.95), rs6059872 (0.95), rs11167245 (0.95), rs6059938 (0.95), rs57654090 (0.94), rs6088527 (0.94), rs11904852 (0.94), rs6087580 (0.93), rs6120620 (0.93), rs1853057 (0.93), rs6059743 (0.92), rs6087592 (0.92), rs6142205 (0.92), rs6060015 (0.91), rs1205338 (0.91), rs6059987 (0.91), rs6087618 (0.9), rs6058070 (0.9), rs6088475 (0.9), rs6141464 (0.89), rs4258875 (0.89), rs7271970 (0.89), rs6088524 (0.88), rs4431018 (0.88), rs6119497 (0.88), rs6059868 (0.87), rs6060013 (0.87), rs6058067 (0.87), rs6120668 (0.86), rs2378203 (0.86), rs6060057 (0.86), rs764597 (0.86), rs4911417 (0.85), rs6141493 (0.85), rs6142159 (0.85), rs6087605 (0.84), rs6059858 (0.84), rs6058040 (0.84), rs6579161 (0.83), rs6059934 (0.83), rs6087574 (0.83), rs6060012 (0.82), rs6058050 (0.82), rs2889850 (0.82), rs2378135 (0.81), rs6059867 (0.81), rs6088489 (0.81), rs6059932 (0.8), rs6087612 (0.8), rs8114616 (0.8), rs6088484 (0.79), rs1205342 (0.79), rs4911152 (0.79), rs6088532 (0.78), rs4911434 (0.78), rs6088536 (0.78), rs4911419 (0.77), rs910869 (0.77), rs910870 (0.77), rs6059863 (0.77), rs6142213 (0.76), rs6088523 (0.76), rs6058052 (0.76), rs6088488 (0.75), rs819140 (0.75), rs6088461 (0.75), rs8183502 (0.74), rs6088577 (0.74), rs2225837 (0.74), rs67439923 (0.73), rs6058073 (0.73), rs4911422 (0.73), rs6087600 (0.72), rs6087587 (0.72), rs13044413 (0.72), rs56049597 (0.71), rs1853056 (0.71), rs6059905 (0.71), rs1205335 (0.7), rs6059877 (0.7), rs2378251 (0.7), rs6088502 (0.69), rs4911421 (0.69), rs945674 (0.69), rs6087577 (0.68), rs6088479 (0.68), rs6142209 (0.68), rs12480410 (0.67), rs6059878 (0.67), rs2378259 (0.67), rs6142210 (0.66), rs2378133 (0.66), rs6142219 (0.66), rs13043505 (0.65), rs8125522 (0.65), rs6088478 (0.65), rs6059834 (0.64), rs6059839 (0.64), rs11167241 (0.64), rs4911441 (0.63), rs6059779 (0.63), rs6059844 (0.63), rs6058106 (0.62), rs17092066 (0.62), rs8121066 (0.62), rs6120644 (0.61), rs13042351 (0.61), rs12624640 (0.61), rs6087598 (0.6), rs6088466 (0.6), rs4911433 (0.6), rs2425000 (0.59), rs6141471 (0.59), rs6088525 (0.59), rs533199207 (0.59), rs6058049 (0.58), rs6058059 (0.58), rs6088542 (0.58), rs6120645 (0.57),

		rs6142208 (0.57), rs7274854 (0.57), rs6142154 (0.56), rs6142206 (0.56), rs6119510 (0.56), rs1205346 (0.55), rs6120650 (0.55), rs2424993 (0.55), rs4142007 (0.54), rs1205336 (0.54), rs6088569 (0.53), rs2064705 (0.53), rs2378254 (0.53), rs6088512 (0.52), rs6059859 (0.52), rs1015363 (0.52), rs6088498 (0.51), rs7271144 (0.51), rs2378134 (0.51), rs819139 (0.5), rs6059896 (0.5), rs6058065 (0.5), rs6058029 (0.5), rs6088567 (0.49), rs2378205 (0.49), rs6087615 (0.49), rs6088554 (0.48), rs6059797 (0.48), rs1986110 (0.48), rs6088508 (0.47), rs6142152 (0.47), rs6088528 (0.47), rs7269596 (0.46), rs6088548 (0.46), rs4911423 (0.46), rs6060052 (0.45), rs6059851 (0.45), rs6060039 (0.45), rs6142137 (0.44), rs6088595 (0.44), rs6087579 (0.44), rs1040747 (0.43), rs6087619 (0.43), rs6058108 (0.42), rs6059835 (0.42), rs73610830 (0.42), rs1205341 (0.41), rs6059882 (0.41), rs6059774 (0.41), rs6088568 (0.41), rs62213590 (0.4), rs1890000 (0.4), rs819135 (0.4), rs6141488 (0.39), rs6120642 (0.39), rs1205337 (0.39), rs6058041 (0.38), rs6059855 (0.38), rs72497006 (0.38), rs6088529 (0.37), rs6142212 (0.37), rs3761147 (0.37), rs6058084 (0.36), rs4911429 (0.36), rs4911437 (0.36), rs6120663 (0.35), rs6088553 (0.35), rs6087588 (0.35), rs4911418 (0.34), rs1205340 (0.34), rs7354735 (0.34), rs4911420 (0.33), rs2105109 (0.33), rs6058057 (0.33), rs6088560 (0.32), rs62212172 (0.32), rs6088547 (0.32), rs6059829 (0.32), rs6141514 (0.31), rs6087616 (0.31), rs6142164 (0.31), rs6120675 (0.3), rs4911445 (0.3), rs6058064 (0.3), rs2378256 (0.29), rs2184836 (0.29), rs6059918 (0.29), rs1205347 (0.28), rs8122820 (0.28), rs6141486 (0.28), rs55793516 (0.27), rs6060038 (0.27), rs6120669 (0.27), rs6059824 (0.26), rs11167234 (0.26), rs6088485 (0.25), rs6059887 (0.25), rs6088543 (0.25), rs6087609 (0.24), rs2424997 (0.24), rs6088552 (0.24), rs6142153 (0.23), rs6059850 (0.23), rs6059856 (0.23), rs6120666 (0.23), rs3736762 (0.22), rs6087624 (0.22), rs562493105 (0.22), rs2378204 (0.21), rs6141509 (0.21), rs3746455 (0.21), rs6059843 (0.2), rs2180485 (0.2), rs6142222 (0.2), rs1321306 (0.19), rs2424992 (0.19), rs1890002 (0.19), rs6141482 (0.18), rs6059880 (0.18), rs6142201 (0.18), rs6142202 (0.17), rs6060048 (0.17), rs6141504 (0.17), rs3891369 (0.16), rs6141503 (0.16), rs6088561 (0.16), rs6088565 (0.15), rs6142216 (0.15), rs6059926 (0.15), rs6088570 (0.14), rs6088564 (0.14), rs6087567 (0.14), rs6142147 (0.14), rs146006879 (0.13), rs2378250 (0.13), rs7264012 (0.13), rs6059890 (0.12), rs6579165 (0.12), rs6059875 (0.12), rs3928101 (0.11), rs4911444 (0.11), rs6088471 (0.11), rs62213657 (0.1), rs1001425 (0.1), rs6059860 (0.1), rs4277599 (0.09), rs6088578 (0.09), rs6142157 (0.09), rs6059861 (0.08), rs6059866 (0.08), rs13043504 (0.08), rs4911153 (0.07), rs1205344 (0.07), rs6088537 (0.07), rs6087614 (0.06), rs6059840 (0.06), rs6059892 (0.06), rs6141469 (0.05), rs6058051 (0.05), rs6059897 (0.05), rs1205343 (0.05), rs6059827 (0.04), rs1205334 (0.04), rs6579178 (0.04), rs721613 (0.03), rs6088483 (0.03), rs945673 (0.03), rs13041646 (0.02), rs2889855 (0.02), rs3787219 (0.02), rs6142129 (0.01), rs6088521 (0.01), rs2295444 (0.01), rs6088575 (0)
rs72771478	1	rs72771479 (0.5)

rs73045226	17	rs73043273 (1), rs56254331 (0.94), rs73045223 (0.89), rs55994820 (0.83), rs8109440 (0.78), rs12461203 (0.72), rs139833223 (0.67), rs73045231 (0.61), rs15052 (0.56), rs73045269 (0.44), rs17251589 (0.39), rs4803447 (0.33), rs12459929 (0.28), rs137991784 (0.22), rs12459996 (0.17), rs144025261 (0.11), rs55841050 (0.06)
rs7412	6	rs445925 (1), rs1065853 (0.71), rs190712692 (0.57), rs390082 (0.43), rs141622900 (0.29), rs72654473 (0.14)
rs74557927	8	rs16885622 (1), rs72754154 (0.78), rs72754161 (0.67), rs139669636 (0.56), rs6896054 (0.44), rs59991448 (0.33), rs58896078 (0.22), rs72754158 (0.11)
rs7595075	70	rs549751842 (1), rs2126129 (0.99), rs56321614 (0.97), rs7601944 (0.96), rs7561648 (0.94), rs6755432 (0.93), rs17714252 (0.92), rs62114497 (0.9), rs60149603 (0.89), rs17713879 (0.87), rs2290911 (0.86), rs79716074 (0.85), rs55753056 (0.83), rs79154857 (0.82), rs7588043 (0.8), rs3791220 (0.79), rs55932737 (0.77), rs2306060 (0.76), rs3791221 (0.75), rs7608141 (0.73), rs62114548 (0.72), rs4497901 (0.7), rs11674325 (0.69), rs62114494 (0.68), rs57542652 (0.66), rs7605824 (0.65), rs62114501 (0.63), rs17713568 (0.62), rs17041320 (0.61), rs60484953 (0.59), rs62114505 (0.58), rs17713729 (0.56), rs17713396 (0.55), rs75141812 (0.54), rs6710091 (0.52), rs60843830 (0.51), rs4430979 (0.49), rs11695591 (0.46), rs58461606 (0.45), rs300750 (0.44), rs3828165 (0.42), rs58416198 (0.41), rs1078763 (0.39), rs11553746 (0.38), rs62114544 (0.37), rs55987018 (0.35), rs36216559 (0.34), rs7584915 (0.32), rs62116681 (0.31), rs55936726 (0.3), rs9213 (0.28), rs11674477 (0.27), rs7566279 (0.25), rs7608143 (0.24), rs3791224 (0.23), rs62116682 (0.21), rs56167434 (0.2), rs56285383 (0.18), rs6548219 (0.17), rs6721594 (0.15), rs62114506 (0.14), rs55742348 (0.13), rs59937473 (0.11), rs3791223 (0.1), rs114976176 (0.08), rs394791 (0.07), rs55946380 (0.06), rs56174623 (0.04), rs300751 (0.03), rs62114538 (0.01)
rs7684939	95	rs6815910 (1), rs6820303 (0.98), rs17827974 (0.97), rs3819391 (0.96), rs7665611 (0.95), rs2537861 (0.94), rs2537863 (0.93), rs7675203 (0.92), rs6554196 (0.91), rs2237036 (0.9), rs6554200 (0.89), rs2159935 (0.88), rs2237028 (0.86), rs12498193 (0.85), rs11722325 (0.84), rs6825293 (0.83), rs80336406 (0.82), rs2192158 (0.81), rs7655709 (0.8), rs17827760 (0.79), rs11940731 (0.78), rs2703456 (0.77), rs13121174 (0.76), rs723585 (0.75), rs7656845 (0.74), rs12504135 (0.73), rs2237029 (0.72), rs13140079 (0.71), rs2537860 (0.7), rs13106834 (0.69), rs981959 (0.68), rs7655556 (0.67), rs2282780 (0.66), rs12499367 (0.65), rs999020 (0.64), rs6825657 (0.63), rs6554194 (0.61), rs7694444 (0.6), rs7684000 (0.59), rs4864913 (0.58), rs7679314 (0.57), rs3819387 (0.56), rs34772064 (0.55), rs2237037 (0.54), rs6815651 (0.53), rs2282779 (0.52), rs7663318 (0.51), rs6554193 (0.5), rs3756197 (0.49), rs2855778 (0.48), rs7655588 (0.47), rs11730497 (0.46), rs34284864 (0.45), rs6831020 (0.44), rs3819389 (0.43), rs6554197 (0.42), rs7656088 (0.41), rs3819392 (0.4), rs11133338 (0.39), rs11733357 (0.38), rs759082 (0.36), rs12512899 (0.35), rs7695300

		(0.34), rs11727273 (0.33), rs6554198 (0.32), rs7656858 (0.31), rs2237030 (0.3), rs2237035 (0.29), rs13128858 (0.28), rs2237026 (0.27), rs2017472 (0.26), rs2215726 (0.25), rs2192159 (0.24), rs11736016 (0.23), rs12503461 (0.22), rs3819393 (0.21), rs759083 (0.2), rs2282778 (0.19), rs7694422 (0.18), rs35441406 (0.17), rs6851498 (0.16), rs6554199 (0.15), rs6554195 (0.14), rs999021 (0.13), rs71599647 (0.11), rs4864915 (0.1), rs3819386 (0.09), rs11736073 (0.08), rs12504244 (0.07), rs2237038 (0.06), rs2237031 (0.05), rs2282781 (0.04), rs13138563 (0.03), rs10488849 (0.02), rs1005438 (0.01)
rs7726	101	rs4659374 (1), rs12726439 (0.99), rs3127010 (0.98), rs2494398 (0.97), rs282177 (0.96), rs282176 (0.95), rs2290588 (0.94), rs72651526 (0.93), rs4970514 (0.92), rs9328728 (0.91), rs364977 (0.9), rs444482 (0.89), rs11806427 (0.88), rs9438547 (0.87), rs3790645 (0.86), rs11806829 (0.85), rs657227 (0.84), rs6685806 (0.83), rs61789104 (0.83), rs10458599 (0.82), rs9971001 (0.81), rs6673072 (0.8), rs2045345 (0.79), rs4402166 (0.78), rs11802779 (0.77), rs11247961 (0.76), rs1466286 (0.75), rs383913 (0.74), rs4659375 (0.73), rs61789103 (0.72), rs7522551 (0.7), rs12027388 (0.68), rs12024530 (0.67), rs12046614 (0.66), rs282175 (0.65), rs188158 (0.64), rs392814 (0.63), rs61789119 (0.62), rs2494397 (0.61), rs396997 (0.6), rs1975798 (0.59), rs10902748 (0.58), rs35245195 (0.57), rs12093376 (0.56), rs7539320 (0.55), rs414546 (0.54), rs12403263 (0.53), rs3122646 (0.52), rs1002488 (0.51), rs7543005 (0.5), rs9438626 (0.5), rs12094989 (0.49), rs3127013 (0.48), rs2290589 (0.47), rs9438508 (0.46), rs56241541 (0.45), rs2278979 (0.44), rs4659443 (0.43), rs11802963 (0.42), rs12046497 (0.41), rs7530096 (0.4), rs6693662 (0.39), rs897641 (0.38), rs4970485 (0.37), rs1064196 (0.36), rs2445635 (0.35), rs200937062 (0.34), rs6675469 (0.33), rs55840663 (0.32), rs7524245 (0.31), rs61692526 (0.3), rs447743 (0.29), rs12045800 (0.28), rs4970466 (0.27), rs3816539 (0.26), rs6669111 (0.25), rs7524421 (0.24), rs374007 (0.23), rs3790644 (0.22), rs2316002 (0.21), rs472271 (0.2), rs4970483 (0.19), rs6690458 (0.18), rs11247958 (0.17), rs3811461 (0.17), rs12723936 (0.16), rs34062688 (0.15), rs2445637 (0.14), rs4659444 (0.13), rs6694351 (0.12), rs873151 (0.11), rs17162190 (0.1), rs4659442 (0.09), rs7521032 (0.08), rs369385 (0.07), rs6656196 (0.06), rs12086684 (0.05), rs9438551 (0.04), rs2045344 (0.03), rs2494395 (0.02), rs4970498 (0.01)
rs7736891	68	rs2637030 (1), rs256085 (0.99), rs31302 (0.97), rs1833888 (0.96), rs7707608 (0.94), rs6859800 (0.93), rs256097 (0.91), rs7712618 (0.9), rs693995 (0.88), rs7706455 (0.87), rs10077935 (0.86), rs10042485 (0.84), rs786333 (0.83), rs1593051 (0.81), rs2113046 (0.8), rs7725696 (0.78), rs452871 (0.77), rs6450148 (0.75), rs7737214 (0.74), rs6886576 (0.72), rs530216 (0.71), rs423872 (0.7), rs2607509 (0.68), rs7726200 (0.67), rs10050522 (0.65), rs7723495 (0.64), rs1833885 (0.62), rs10057745 (0.61), rs1423562 (0.59), rs271214 (0.58), rs1345811 (0.57), rs10471875 (0.55), rs6882567 (0.54), rs79099249 (0.52), rs6450149 (0.51), rs1593055 (0.49), rs10050519 (0.48), rs6450145 (0.46), rs10063311 (0.45), rs13158446 (0.43), rs2216718 (0.42), rs6450147

		(0.41), rs4865546 (0.39), rs11951062 (0.38), rs1363965 (0.36), rs7707821 (0.35), rs7709405 (0.33), rs6450146 (0.32), rs11739076 (0.3), rs62370545 (0.29), rs7719960 (0.28), rs4865545 (0.25), rs6450141 (0.23), rs1833887 (0.22), rs6872463 (0.2), rs75686760 (0.19), rs6450144 (0.17), rs813301 (0.16), rs7703235 (0.14), rs12656684 (0.13), rs6450150 (0.12), rs1363973 (0.1), rs6880712 (0.09), rs2637004 (0.07), rs3909459 (0.06), rs200111371 (0.04), rs10041126 (0.03), rs7731038 (0.01)
rs77705851	3	rs78913998 (1), rs148348342 (0.75), rs117756740 (0.25)
rs77913157	0	
rs8078686	291	rs9912592 (1), rs2317826 (1), rs34537623 (0.99), rs9915222 (0.99), rs10853090 (0.99), rs4092460 (0.98), rs11868838 (0.98), rs8075411 (0.98), rs12051716 (0.97), rs8078309 (0.97), rs9895776 (0.97), rs11868058 (0.96), rs11651643 (0.96), rs9899859 (0.96), rs4968259 (0.95), rs1912484 (0.95), rs2610367 (0.95), rs9914653 (0.94), rs4793836 (0.94), rs1912482 (0.94), rs9913014 (0.93), rs6504963 (0.93), rs62073965 (0.93), rs10432035 (0.92), rs8082284 (0.92), rs4968318 (0.91), rs9892686 (0.91), rs9911944 (0.91), rs11079783 (0.9), rs12601144 (0.9), rs12452796 (0.9), rs8076778 (0.89), rs9913206 (0.89), rs7220935 (0.89), rs11650372 (0.88), rs7222851 (0.88), rs77142415 (0.88), rs201513188 (0.87), rs62073970 (0.87), rs12937560 (0.87), rs3865314 (0.86), rs4239162 (0.86), rs6504974 (0.86), rs9897063 (0.85), rs55687600 (0.85), rs10445374 (0.85), rs62076505 (0.84), rs4794048 (0.84), rs140734195 (0.84), rs9900366 (0.83), rs7223029 (0.83), rs28507314 (0.83), rs9635762 (0.82), rs9904967 (0.82), rs73315878 (0.82), rs4968228 (0.81), rs7221224 (0.81), rs11870935 (0.81), rs7209422 (0.8), rs35919601 (0.8), rs36043200 (0.8), rs4375701 (0.79), rs148992138 (0.79), rs11079779 (0.79), rs7222225 (0.78), rs12150231 (0.78), rs8081717 (0.77), rs62076540 (0.77), rs11079775 (0.77), rs72829689 (0.76), rs7214410 (0.76), rs9674670 (0.76), rs2611998 (0.75), rs4793842 (0.75), rs3736438 (0.74), rs9905581 (0.74), rs9905583 (0.74), rs9905922 (0.73), rs9989466 (0.73), rs9901869 (0.73), rs4561509 (0.72), rs8067286 (0.72), rs201496912 (0.72), rs8070759 (0.71), rs8075843 (0.71), rs7221345 (0.71), rs62076549 (0.7), rs2644349 (0.7), rs8079821 (0.7), rs11869730 (0.69), rs12103481 (0.69), rs2258333 (0.69), rs10163469 (0.68), rs34784720 (0.68), rs77454872 (0.68), rs62074014 (0.67), rs8077106 (0.67), rs369648654 (0.67), rs9907159 (0.66), rs62076461 (0.66), rs12602134 (0.66), rs12943464 (0.65), rs2136749 (0.65), rs9895274 (0.65), rs56131210 (0.64), rs9908016 (0.64), rs35806769 (0.64), rs2136750 (0.63), rs3871595 (0.63), rs9912311 (0.63), rs62073966 (0.62), rs200130731 (0.62), rs56325564 (0.62), rs2644364 (0.61), rs59231671 (0.61), rs35016056 (0.61), rs9905308 (0.6), rs9893901 (0.6), rs112812555 (0.6), rs9903898 (0.59), rs9900360 (0.59), rs62074055 (0.59), rs12103836 (0.58), rs6503804 (0.58), rs1912487 (0.57), rs8072644 (0.57), rs4968320 (0.57), rs9894905 (0.56), rs4968317 (0.56), rs7214993 (0.56), rs12600603 (0.55), rs8065990 (0.55), rs12451409 (0.55), rs11870057 (0.54), rs199538746 (0.54), rs8077485

		(0.54), rs7208530 (0.53), rs4267364 (0.53), rs9303533 (0.53), rs8070463 (0.52), rs12936468 (0.52), rs4439799 (0.52), rs6505049 (0.51), rs56017620 (0.51), rs11650210 (0.51), rs11079776 (0.5), rs55633793 (0.5), rs4793978 (0.5), rs4114873 (0.49), rs112438412 (0.49), rs9908393 (0.48), rs4794372 (0.48), rs4794371 (0.48), rs1138193 (0.47), rs34200664 (0.47), rs2935183 (0.47), rs1912485 (0.46), rs78551919 (0.46), rs4794007 (0.46), rs9915919 (0.45), rs11656855 (0.45), rs6505048 (0.45), rs11079784 (0.44), rs12451766 (0.44), rs11656056 (0.44), rs3809863 (0.43), rs4060889 (0.43), rs11656856 (0.43), rs8074149 (0.42), rs1221385 (0.42), rs8075566 (0.41), rs12452315 (0.41), rs141516856 (0.41), rs8074451 (0.4), rs7350906 (0.4), rs4794053 (0.4), rs79443387 (0.39), rs74951133 (0.39), rs11870585 (0.39), rs4239163 (0.38), rs1704808 (0.38), rs3851808 (0.38), rs9904664 (0.37), rs3760370 (0.37), rs57427906 (0.37), rs62076541 (0.36), rs35794672 (0.36), rs12449647 (0.36), rs12944103 (0.35), rs8081556 (0.35), rs4497739 (0.35), rs62074015 (0.34), rs34609599 (0.34), rs9894179 (0.34), rs9916007 (0.33), rs3826536 (0.33), rs9889709 (0.33), rs9807106 (0.32), rs62076542 (0.32), rs8065669 (0.32), rs11869940 (0.31), rs11079774 (0.31), rs2125873 (0.31), rs4794052 (0.3), rs4338849 (0.3), rs4794047 (0.3), rs11651766 (0.29), rs8065099 (0.29), rs28458768 (0.29), rs35159316 (0.28), rs145346039 (0.28), rs9897492 (0.28), rs11079782 (0.27), rs4289035 (0.27), rs56356751 (0.27), rs4399567 (0.26), rs7207542 (0.26), rs6505062 (0.26), rs56952963 (0.25), rs3809868 (0.25), rs9900123 (0.24), rs9892497 (0.24), rs6503796 (0.24), rs6504872 (0.23), rs2644383 (0.23), rs567322758 (0.23), rs8079744 (0.22), rs2175290 (0.22), rs4968319 (0.22), rs7405889 (0.21), rs9891111 (0.21), rs11871606 (0.21), rs9897957 (0.2), rs4968257 (0.2), rs2136751 (0.2), rs2317631 (0.19), rs7219173 (0.19), rs55864353 (0.19), rs7210801 (0.18), rs7214799 (0.18), rs7210472 (0.18), rs1912486 (0.17), rs9902574 (0.17), rs9903100 (0.17), rs58512566 (0.16), rs8065437 (0.16), rs9894594 (0.16), rs4794058 (0.15), rs6503807 (0.15), rs3883319 (0.14), rs8072100 (0.14), rs1962307 (0.14), rs9912101 (0.13), rs9890782 (0.13), rs7211910 (0.13), rs8075058 (0.12), rs4794051 (0.12), rs4793908 (0.12), rs9913503 (0.11), rs9895746 (0.11), rs200908868 (0.11), rs7226079 (0.1), rs67919208 (0.1), rs12603290 (0.1), rs9908049 (0.09), rs1912483 (0.09), rs4580230 (0.09), rs3874910 (0.08), rs7213086 (0.08), rs7219303 (0.07), rs11655943 (0.07), rs9892234 (0.07), rs4794001 (0.06), rs12453233 (0.06), rs7210738 (0.06), rs62074054 (0.05), rs2292346 (0.05), rs4794016 (0.05), rs8079142 (0.04), rs67688344 (0.04), rs4793913 (0.04), rs2085755 (0.03), rs9905073 (0.03), rs8078880 (0.03), rs3760371 (0.02), rs7221548 (0.02), rs7206971 (0.02), rs10048173 (0.01), rs9635763 (0.01), rs4264433 (0.01), rs9895509 (0)
rs9268839	631	rs34835435 (1), rs9394101 (1), rs535667342 (1), rs9270559 (1), rs141506183 (0.99), rs9268846 (0.99), rs28393315 (0.99), rs9270355 (0.99), rs28895267 (0.99), rs78121285 (0.99), rs6938337 (0.98), rs9275567 (0.98), rs9275526 (0.98), rs28366317 (0.98), rs2395192 (0.98), rs12191360 (0.98), rs9275574 (0.97), rs73728002 (0.97), rs72849268 (0.97), rs1130161 (0.97),

rs6906730 (0.97), rs7769693 (0.97), rs28366328 (0.97), rs28895225 (0.96), rs151027857 (0.96), rs28752527 (0.96), rs5007263 (0.96), rs9269135 (0.96), rs2076533 (0.96), rs9271568 (0.95), rs9271430 (0.95), rs9270589 (0.95), rs9270080 (0.95), rs72851018 (0.95), rs28732284 (0.95), rs9271628 (0.95), rs9348886 (0.94), rs9275533 (0.94), rs9378212 (0.94), rs9268853 (0.94), rs4516986 (0.94), rs6932810 (0.94), rs17203570 (0.93), rs9271592 (0.93), rs28599891 (0.93), rs184050448 (0.93), rs9269144 (0.93), rs9275522 (0.93), rs17209907 (0.92), rs9268969 (0.92), rs9271565 (0.92), rs9271560 (0.92), rs569809786 (0.92), rs28366314 (0.92), rs111875855 (0.92), rs28383314 (0.91), rs9275539 (0.91), rs9275547 (0.91), rs28383313 (0.91), rs3817973 (0.91), rs9275575 (0.91), rs9268860 (0.9), rs1048173 (0.9), rs548599006 (0.9), rs2647018 (0.9), rs28732268 (0.9), rs17203542 (0.9), rs9275565 (0.89), rs2856668 (0.89), rs9275556 (0.89), rs71549254 (0.89), rs9268506 (0.89), rs552565118 (0.89), rs17203549 (0.89), rs71565333 (0.88), rs28383457 (0.88), rs9271587 (0.88), rs567082101 (0.88), rs4947345 (0.88), rs540527828 (0.88), rs9270558 (0.87), rs9271544 (0.87), rs180852758 (0.87), rs9270593 (0.87), rs77604461 (0.87), rs9271624 (0.87), rs9268923 (0.87), rs113938016 (0.86), rs17209950 (0.86), rs9391877 (0.86), rs2856699 (0.86), rs71543454 (0.86), rs62405565 (0.86), rs9268838 (0.85), rs111899294 (0.85), rs561145488 (0.85), rs72853949 (0.85), rs5007260 (0.85), rs9268849 (0.85), rs28383230 (0.84), rs139201366 (0.84), rs71565331 (0.84), rs12194148 (0.84), rs540590778 (0.84), rs28895235 (0.84), rs9275517 (0.84), rs28383223 (0.83), rs28383236 (0.83), rs28895265 (0.83), rs35675330 (0.83), rs28724008 (0.83), rs9271589 (0.83), rs72845704 (0.82), rs9275568 (0.82), rs112232311 (0.82), rs9405108 (0.82), rs72492322 (0.82), rs111842802 (0.82), rs71565335 (0.81), rs9268520 (0.81), rs28383242 (0.81), rs9271596 (0.81), rs7775108 (0.81), rs2647020 (0.81), rs35773267 (0.8), rs9271571 (0.8), rs9271607 (0.8), rs2647013 (0.8), rs2647016 (0.8), rs75076975 (0.8), rs9270550 (0.79), rs17203514 (0.79), rs552798572 (0.79), rs9271584 (0.79), rs968155 (0.79), rs9275527 (0.79), rs9271585 (0.79), rs4457186 (0.78), rs9271621 (0.78), rs477515 (0.78), rs9269137 (0.78), rs71565340 (0.78), rs6932542 (0.78), rs28383181 (0.77), rs74958579 (0.77), rs6457594 (0.77), rs112280675 (0.77), rs9268507 (0.77), rs536427156 (0.77), rs1964995 (0.76), rs9275524 (0.76), rs9268965 (0.76), rs9269110 (0.76), rs9378266 (0.76), rs9275544 (0.76), rs7747529 (0.76), rs5020946 (0.75), rs2856727 (0.75), rs9380309 (0.75), rs9275580 (0.75), rs9275525 (0.75), rs35399335 (0.75), rs113520250 (0.74), rs9275561 (0.74), rs9270806 (0.74), rs9275540 (0.74), rs9275546 (0.74), rs34452456 (0.74), rs9271563 (0.74), rs9268889 (0.73), rs9269157 (0.73), rs9268834 (0.73), rs7452937 (0.73), rs9268835 (0.73), rs9275581 (0.73), rs9272317 (0.72), rs9269226 (0.72), rs28752516 (0.72), rs2646998 (0.72), rs476602 (0.72), rs9268513 (0.72), rs28895234 (0.71), rs2858320 (0.71), rs2516051 (0.71), rs9270598 (0.71), rs5007262 (0.71), rs553260480 (0.71), rs9275521 (0.71), rs9270126 (0.7), rs531127489 (0.7), rs114776163 (0.7), rs9268858 (0.7), rs29001620 (0.7), rs5020947 (0.7), rs9268519

(0.69), rs28366316 (0.69), rs9275515 (0.69), rs9271480 (0.69), rs9269237 (0.69), rs114932108 (0.69), rs9268906 (0.68), rs4994855 (0.68), rs9271574 (0.68), rs111409670 (0.68), rs2516050 (0.68), rs71565334 (0.68), rs9271561 (0.68), rs9268970 (0.67), rs3828832 (0.67), rs79251508 (0.67), rs561689382 (0.67), rs9271578 (0.67), rs116254278 (0.67), rs9270562 (0.66), rs62405562 (0.66), rs9368725 (0.66), rs9271416 (0.66), rs9270597 (0.66), rs9269873 (0.66), rs2647046 (0.66), rs9268864 (0.65), rs28559730 (0.65), rs5007261 (0.65), rs570194309 (0.65), rs535476317 (0.65), rs9271769 (0.65), rs6911383 (0.64), rs35539407 (0.64), rs9271485 (0.64), rs9275558 (0.64), rs2395186 (0.64), rs9275579 (0.64), rs28732247 (0.63), rs9268504 (0.63), rs9405110 (0.63), rs73729111 (0.63), rs112158360 (0.63), rs9270549 (0.63), rs9271438 (0.63), rs4410767 (0.62), rs548717541 (0.62), rs9271599 (0.62), rs9378213 (0.62), rs2213586 (0.62), rs522308 (0.62), rs9268670 (0.61), rs2858324 (0.61), rs9271586 (0.61), rs28895247 (0.61), rs2858319 (0.61), rs2395153 (0.61), rs9271516 (0.6), rs9270115 (0.6), rs113459411 (0.6), rs71549283 (0.6), rs9268435 (0.6), rs77594561 (0.6), rs12660447 (0.59), rs28383439 (0.59), rs9271408 (0.59), rs9275339 (0.59), rs9275534 (0.59), rs76870762 (0.59), rs9269178 (0.58), rs9405109 (0.58), rs28366308 (0.58), rs5020950 (0.58), rs7759742 (0.58), rs28366319 (0.58), rs79346779 (0.58), rs28895249 (0.57), rs5007265 (0.57), rs9268791 (0.57), rs35372060 (0.57), rs2894261 (0.57), rs9271637 (0.57), rs28383233 (0.56), rs9275518 (0.56), rs9268905 (0.56), rs28732246 (0.56), rs28895253 (0.56), rs571314744 (0.56), rs2858318 (0.55), rs71565336 (0.55), rs35957455 (0.55), rs9275505 (0.55), rs28662053 (0.55), rs9271406 (0.55), rs9271474 (0.54), rs9275570 (0.54), rs9271409 (0.54), rs2647048 (0.54), rs201574337 (0.54), rs9270556 (0.54), rs28752515 (0.53), rs1129735 (0.53), rs9275571 (0.53), rs4935354 (0.53), rs9271595 (0.53), rs9272435 (0.53), rs17209887 (0.53), rs35081742 (0.52), rs9271511 (0.52), rs6932517 (0.52), rs72844152 (0.52), rs28366309 (0.52), rs9270521 (0.52), rs9391879 (0.51), rs9275582 (0.51), rs2516052 (0.51), rs9275573 (0.51), rs4373382 (0.51), rs71543455 (0.51), rs9268765 (0.5), rs9270576 (0.5), rs35095850 (0.5), rs2647040 (0.5), rs9275577 (0.5), rs7192 (0.5), rs9271761 (0.5), rs9271540 (0.49), rs28366325 (0.49), rs528864352 (0.49), rs536151678 (0.49), rs562057037 (0.49), rs28383241 (0.49), rs28895257 (0.48), rs28732243 (0.48), rs9271576 (0.48), rs72844171 (0.48), rs35875942 (0.48), rs28383231 (0.48), rs77789453 (0.47), rs536493456 (0.47), rs4994859 (0.47), rs9270583 (0.47), rs9270538 (0.47), rs9269114 (0.47), rs2647001 (0.47), rs3188543 (0.46), rs149088066 (0.46), rs9391881 (0.46), rs9286790 (0.46), rs9275528 (0.46), rs12215313 (0.46), rs28732244 (0.45), rs9268790 (0.45), rs552189104 (0.45), rs9269138 (0.45), rs9271472 (0.45), rs9271411 (0.45), rs2187822 (0.45), rs35239661 (0.44), rs968154 (0.44), rs9378268 (0.44), rs3998157 (0.44), rs17203846 (0.44), rs2395185 (0.44), rs9268434 (0.43), rs9271469 (0.43), rs9271605 (0.43), rs13194665 (0.43), rs9275542 (0.43), rs9270363 (0.43), rs9268815 (0.42), rs9271488 (0.42), rs5020944 (0.42), rs112112734 (0.42), rs563837736 (0.42), rs9272775 (0.42),

rs9378264 (0.42), rs9380306 (0.41), rs9270574 (0.41), rs570919834 (0.41), rs4273729 (0.41), rs28895223 (0.41), rs29001568 (0.41), rs113395139 (0.4), rs28895241 (0.4), rs9269211 (0.4), rs9270580 (0.4), rs9269142 (0.4), rs12215261 (0.39), rs9268476 (0.39), rs7749242 (0.39), rs2858305 (0.39), rs7743415 (0.39), rs7754768 (0.39), rs9270121 (0.39), rs3763327 (0.38), rs9271549 (0.38), rs9271643 (0.38), rs114031016 (0.38), rs112587701 (0.38), rs9271532 (0.38), rs75518178 (0.37), rs9275513 (0.37), rs9268951 (0.37), rs12207473 (0.37), rs9268501 (0.37), rs9271582 (0.37), rs9275578 (0.37), rs28653112 (0.36), rs2856670 (0.36), rs71549252 (0.36), rs536833125 (0.36), rs2076529 (0.36), rs17203612 (0.36), rs9269749 (0.35), rs62405567 (0.35), rs6924676 (0.35), rs9271620 (0.35), rs9268686 (0.35), rs9275523 (0.35), rs2894262 (0.34), rs9271588 (0.34), rs6923504 (0.34), rs9275555 (0.34), rs111343881 (0.34), rs5007264 (0.34), rs9271470 (0.34), rs9272420 (0.33), rs9275506 (0.33), rs28895259 (0.33), rs9270590 (0.33), rs9275536 (0.33), rs75259234 (0.33), rs113026095 (0.32), rs29001478 (0.32), rs9405040 (0.32), rs9275531 (0.32), rs17210202 (0.32), rs9275545 (0.32), rs2647012 (0.32), rs28383224 (0.31), rs113787446 (0.31), rs2647021 (0.31), rs9368726 (0.31), rs554832027 (0.31), rs6926737 (0.31), rs28732242 (0.3), rs28437337 (0.3), rs9271546 (0.3), rs9271426 (0.3), rs5007258 (0.3), rs2647043 (0.3), rs9271547 (0.29), rs113534515 (0.29), rs28383460 (0.29), rs28383310 (0.29), rs112073772 (0.29), rs9275557 (0.29), rs2281276 (0.29), rs2227139 (0.28), rs67081540 (0.28), rs4587163 (0.28), rs9271642 (0.28), rs28619202 (0.28), rs28454881 (0.28), rs9268402 (0.27), rs2856666 (0.27), rs2213584 (0.27), rs2395191 (0.27), rs28895258 (0.27), rs9271583 (0.27), rs77777792 (0.26), rs9275512 (0.26), rs9270570 (0.26), rs2647071 (0.26), rs9269109 (0.26), rs28366315 (0.26), rs6903608 (0.26), rs9269176 (0.25), rs35007143 (0.25), rs17203619 (0.25), rs28752514 (0.25), rs9366801 (0.25), rs9269139 (0.25), rs28383221 (0.24), rs17209964 (0.24), rs9391781 (0.24), rs9275569 (0.24), rs7194 (0.24), rs34403271 (0.24), rs2856687 (0.24), rs9271593 (0.23), rs17203636 (0.23), rs77469652 (0.23), rs71543453 (0.23), rs12195582 (0.23), rs9275529 (0.23), rs2647051 (0.22), rs71549253 (0.22), rs534819727 (0.22), rs9268668 (0.22), rs9275516 (0.22), rs9275212 (0.22), rs561623787 (0.21), rs62405566 (0.21), rs9268502 (0.21), rs9270567 (0.21), rs9405112 (0.21), rs35957722 (0.21), rs9271609 (0.21), rs17203563 (0.2), rs3763314 (0.2), rs2647022 (0.2), rs79452641 (0.2), rs116432881 (0.2), rs28732291 (0.2), rs28723989 (0.19), rs9269175 (0.19), rs9271497 (0.19), rs2213585 (0.19), rs28654725 (0.19), rs113399573 (0.19), rs476775 (0.18), rs9271564 (0.18), rs28895236 (0.18), rs28895266 (0.18), rs12195589 (0.18), rs9270045 (0.18), rs34207893 (0.18), rs2395206 (0.17), rs111517012 (0.17), rs28895264 (0.17), rs9275389 (0.17), rs2858310 (0.17), rs2856722 (0.17), rs2856725 (0.16), rs9268783 (0.16), rs9268884 (0.16), rs3129755 (0.16), rs28952268 (0.16), rs9268832 (0.16), rs376327303 (0.16), rs556025 (0.15), rs9405038 (0.15), rs115272081 (0.15), rs28895261 (0.15), rs9268472 (0.15), rs9275572 (0.15), rs190685297 (0.14), rs28383406 (0.14), rs111343297 (0.14),

		rs17209866 (0.14), rs6918317 (0.14), rs113134152 (0.14), rs5018660 (0.13), rs685244 (0.13), rs6919855 (0.13), rs28383311 (0.13), rs397843773 (0.13), rs2076530 (0.13), rs9271763 (0.13), rs28895255 (0.12), rs563197321 (0.12), rs527369852 (0.12), rs622137 (0.12), rs9271604 (0.12), rs35984981 (0.12), rs28366327 (0.11), rs29001652 (0.11), rs9271542 (0.11), rs9275564 (0.11), rs9269254 (0.11), rs112530924 (0.11), rs13194770 (0.11), rs74927567 (0.1), rs9271591 (0.1), rs9268880 (0.1), rs28732302 (0.1), rs34036879 (0.1), rs28895254 (0.09), rs9269172 (0.09), rs2856723 (0.09), rs28366307 (0.09), rs115752743 (0.09), rs17882603 (0.09), rs2858309 (0.08), rs112296425 (0.08), rs9269174 (0.08), rs17203626 (0.08), rs28895250 (0.08), rs35139284 (0.08), rs7746327 (0.07), rs4424066 (0.07), rs73729140 (0.07), rs9275345 (0.07), rs5007266 (0.07), rs9271394 (0.07), rs9394099 (0.06), rs34614226 (0.06), rs9275535 (0.06), rs12197312 (0.06), rs17209873 (0.06), rs28366298 (0.06), rs9270034 (0.05), rs9268503 (0.05), rs9275548 (0.05), rs13211921 (0.05), rs78279385 (0.05), rs7195 (0.05), rs9271573 (0.05), rs9268473 (0.04), rs9270578 (0.04), rs2133035 (0.04), rs2856717 (0.04), rs28895242 (0.04), rs9272774 (0.04), rs9268508 (0.03), rs4502931 (0.03), rs9275538 (0.03), rs9268518 (0.03), rs4994854 (0.03), rs2647003 (0.03), rs78510027 (0.03), rs9270592 (0.02), rs9270807 (0.02), rs7748270 (0.02), rs9268460 (0.02), rs5007259 (0.02), rs35971290 (0.01), rs28895224 (0.01), rs9271504 (0.01), rs9269141 (0.01), rs9270572 (0.01), rs111757290 (0.01), rs9275585 (0), rs9394098 (0), rs9268833 (0)
rs9609261	8	rs4820044 (1), rs2040532 (0.89), rs80224973 (0.78), rs138286 (0.67), rs5753432 (0.56), rs5753760 (0.44), rs112058175 (0.22), rs80180094 (0.11)

ALS: Amyotrophic lateral sclerosis; HDL-C: high-density lipoprotein cholesterol; SNP: single nucleotide polymorphisms.
