# Supplementary material for: Shared genetic link and causal inference between blood lipids, lipid-lowering drugs and amyotrophic lateral sclerosis
Source: Neural Regen Res. 2025 Aug 13;21(8):3824–30. doi: 10.4103/NRR.NRR-D-25-00266 (PMC13452702; doi:10.4103/NRR.NRR-D-25-00266)
Supplement: Supplementary file 2 [file NRR-21-3824_Suppl2.pdf]

Additional Table 5 List of credible set SNPs in each shared locus between ALS and ApoA1 from fine mapping

SNP	No. credible set SNPs	List of credible set SNPs (posterior probability in brackets)
rs1022186	24	rs73243890 (1), rs6448429 (0.96), rs9884830 (0.92), rs17630194 (0.88), rs6846421 (0.8), rs6819735 (0.76), rs2137236 (0.72), rs10019888 (0.68), rs56122753 (0.64), rs56147366 (0.6), rs28673139 (0.56), rs2137234 (0.52), rs2048507 (0.48), rs2048508 (0.44), rs12502605 (0.4), rs7698037 (0.36), rs73243877 (0.32), rs1567651 (0.28), rs12513287 (0.24), rs1912897 (0.2), rs2137235 (0.16), rs12498488 (0.12), rs4558863 (0.08), rs73243884 (0.04)
rs10505258	177	rs2178948 (0.99), rs2142331 (0.99), rs2737216 (0.98), rs2737219 (0.98), rs10808475 (0.97), rs1483582 (0.97), rs1057517 (0.96), rs1905376 (0.96), rs10093834 (0.95), rs2625673 (0.94), rs2293889 (0.94), rs800880 (0.93), rs6469605 (0.93), rs6990008 (0.92), rs3779878 (0.92), rs10955751 (0.91), rs6469597 (0.91), rs1180624 (0.9), rs6998144 (0.89), rs3808428 (0.89), rs3808430 (0.88), rs2625674 (0.88), rs2049865 (0.87), rs2009382 (0.87), rs2737215 (0.86), rs10093833 (0.85), rs1963677 (0.85), rs3808439 (0.84), rs2721954 (0.84), rs2049867 (0.83), rs3808460 (0.83), rs3802219 (0.82), rs2625672 (0.82), rs3808454 (0.81), rs1384812 (0.8), rs3808457 (0.8), rs11989423 (0.79), rs3779881 (0.79), rs2721933 (0.78), rs6986967 (0.78), rs3808456 (0.77), rs12543799 (0.77), rs3808448 (0.76), rs1351744 (0.75), rs6993253 (0.75), rs3808461 (0.74), rs2737214 (0.74), rs3808434 (0.73), rs800881 (0.73), rs997806 (0.72), rs2223103 (0.72), rs12114740 (0.71), rs3802220 (0.7), rs2737217 (0.7), rs3808415 (0.69), rs12056626 (0.69), rs2245221 (0.68), rs9643105 (0.68), rs2721936 (0.67), rs800870 (0.66), rs2625675 (0.66), rs10097784 (0.65), rs2721940 (0.65), rs2721926 (0.64), rs3808416 (0.64), rs10955753 (0.63), rs13275142 (0.63), rs2358019 (0.62), rs2737205 (0.61), rs10955752 (0.61), rs6469600 (0.6), rs2721938 (0.6), rs13269499 (0.59), rs2721942 (0.58), rs2721929 (0.58), rs2170741 (0.57), rs800908 (0.56), rs3808438 (0.56), rs2737227 (0.55), rs1180647 (0.55), rs1384810 (0.54), rs800882 (0.54), rs10505256 (0.53), rs7834300 (0.53), rs1483583 (0.52), rs1483585 (0.51), rs28447075 (0.51), rs2737225 (0.5), rs2049864 (0.5), rs800909 (0.49), rs6469603 (0.49), rs3779879 (0.48), rs800887 (0.47), rs7832568 (0.47), rs2200218 (0.46), rs719422 (0.46), rs3808444 (0.45), rs3808418 (0.45), rs2721932 (0.44), rs3808425 (0.44), rs3808435 (0.43), rs3808459 (0.42), rs3808451 (0.42), rs1963678 (0.41), rs12549788 (0.41), rs2721934 (0.4), rs11778934 (0.4), rs2170740 (0.39), rs2245019 (0.39), rs2272619 (0.38), rs800878 (0.37), rs9297541 (0.37), rs3808426 (0.36), rs7823575 (0.36), rs2178949 (0.35), rs2737220 (0.35), rs1079822 (0.34), rs809623 (0.34), rs2178947 (0.33), rs1483589 (0.32), rs800884 (0.32), rs12679749 (0.31), rs3849850 (0.31), rs1160333 (0.3), rs800868 (0.3), rs13267535 (0.29), rs7813434 (0.28), rs1569364 (0.28), rs6469601 (0.27), rs800888 (0.27), rs4599845 (0.26), rs3802222 (0.26), rs1028274 (0.25), rs7357472 (0.25), rs800910 (0.24), rs3808447 (0.23), rs3808446 (0.23), rs2003643 (0.22), rs2721946 (0.22), rs1180648 (0.21), rs11785504 (0.21), rs2737226 (0.2), rs3808437 (0.2), rs13271228 (0.19), rs2884366 (0.18), rs2737207 (0.18), rs4326410 (0.17), rs3808450 (0.17), rs2103481 (0.16), rs3808440 (0.16), rs3808449 (0.15), rs6998448 (0.15), rs2737206 (0.14), rs3802221 (0.13), rs2178946 (0.13), rs800879 (0.12), rs13266941 (0.12), rs2721943 (0.11), rs7011494 (0.11), rs2721939 (0.1),

		rs7012216 (0.09), rs3808422 (0.09), rs800883 (0.08), rs3808436 (0.08), rs2721948 (0.07), rs12548221 (0.07), rs2358021 (0.06), rs800869 (0.06), rs2737203 (0.05), rs2737212 (0.04), rs11786044 (0.04), rs7825679 (0.03), rs800886 (0.03), rs800871 (0.02), rs12677858 (0.02), rs6469606 (0.01), rs3824200 (0.01)
rs10774625	16	rs3184504 (1), rs10774624 (0.94), rs847888 (0.88), rs4766897 (0.76), rs2013002 (0.71), rs597808 (0.65), rs653178 (0.59), rs11513729 (0.53), rs11065987 (0.47), rs1265565 (0.41), rs7137828 (0.35), rs4766578 (0.29), rs11065979 (0.24), rs1265564 (0.18), rs11065991 (0.12), rs7310615 (0.06)
rs11021232	3	rs7933222 (1), rs4409785 (0.5), rs12575636 (0.25)
rs11039324	329	rs7110300 (1), rs4752857 (1), rs4752832 (0.99), rs10838775 (0.99), rs6485778 (0.99), rs1057233 (0.98), rs2293577 (0.98), rs11039416 (0.98), rs12361256 (0.98), rs4752801 (0.97), rs1471712 (0.97), rs11039402 (0.97), rs11607981 (0.96), rs28617307 (0.96), rs6485786 (0.96), rs2904127 (0.95), rs60833259 (0.95), rs1483988 (0.95), rs11039391 (0.95), rs11039219 (0.94), rs11602339 (0.94), rs58965622 (0.94), rs7111509 (0.93), rs12803525 (0.93), rs755553 (0.93), rs755554 (0.92), rs7123952 (0.92), rs7943464 (0.92), rs7120333 (0.92), rs11039307 (0.91), rs11604680 (0.91), rs10769300 (0.91), rs7929725 (0.9), rs10838771 (0.9), rs7106956 (0.9), rs7939420 (0.89), rs7105851 (0.89), rs112060627 (0.89), rs11039355 (0.89), rs7105791 (0.88), rs11600581 (0.88), rs12802273 (0.88), rs7107535 (0.87), rs11605672 (0.87), rs67472071 (0.87), rs3781627 (0.86), rs34467936 (0.86), rs2290850 (0.86), rs7105282 (0.86), rs12419507 (0.85), rs10838757 (0.85), rs12418852 (0.85), rs10838786 (0.84), rs1317164 (0.84), rs7124681 (0.84), rs754434 (0.83), rs2084946 (0.83), rs11039308 (0.83), rs11601173 (0.83), rs2869024 (0.82), rs2125838 (0.82), rs2170815 (0.82), rs10769285 (0.81), rs4752858 (0.81), rs10742820 (0.81), rs71475989 (0.8), rs6485773 (0.8), rs1064608 (0.8), rs6485794 (0.8), rs4752873 (0.79), rs7101597 (0.79), rs12421210 (0.79), rs7928419 (0.78), rs4752987 (0.78), rs4752797 (0.78), rs2053979 (0.77), rs7925299 (0.77), rs11039238 (0.77), rs12286721 (0.77), rs3781625 (0.76), rs12785833 (0.76), rs3740699 (0.76), rs11039280 (0.75), rs56030824 (0.75), rs12362318 (0.75), rs7937331 (0.74), rs7130758 (0.74), rs10838738 (0.74), rs1056387 (0.74), rs7927771 (0.73), rs11601603 (0.73), rs12785949 (0.73), rs7107792 (0.72), rs7924485 (0.72), rs10838759 (0.72), rs11039377 (0.71), rs12808892 (0.71), rs4752796 (0.71), rs7129797 (0.71), rs10838769 (0.7), rs10838745 (0.7), rs4752860 (0.7), rs7943213 (0.69), rs35184771 (0.69), rs12798109 (0.69), rs10838748 (0.68), rs755555 (0.68), rs11039412 (0.68), rs7108479 (0.67), rs12146565 (0.67), rs11039199 (0.67), rs12787112 (0.66), rs4752838 (0.66), rs74608150 (0.66), rs11039212 (0.65), rs7119517 (0.65), rs7950696 (0.64), rs3781624 (0.64), rs11039195 (0.64), rs113945456 (0.63), rs10838699 (0.63), rs12576933 (0.63), rs11039203 (0.62), rs2030166 (0.62), rs10742814 (0.62), rs10838766 (0.62), rs2293580 (0.61), rs7925389 (0.61), rs10734557 (0.61), rs11039348 (0.6), rs146014399 (0.6), rs11039202 (0.6), rs78187521 (0.59), rs2869541 (0.59), rs10838732 (0.59), rs35624992 (0.59), rs11039265 (0.58), rs3758655 (0.58), rs11605774 (0.58), rs10838747 (0.57), rs12803191 (0.57), rs6485756 (0.57), rs7935344 (0.56), rs1563820 (0.56), rs7950674 (0.56), rs1542321 (0.56), rs35996350 (0.55), rs7932703 (0.55), rs935914 (0.55), rs11039447 (0.54), rs12360643 (0.54), rs10128705 (0.54), rs4543938 (0.53), rs34910028 (0.53), rs2869534 (0.53),

		rs12366244 (0.53), rs10838774 (0.52), rs7933184 (0.52), rs2046377 (0.52), rs10838784 (0.51), rs7116346 (0.51), rs7945473 (0.51), rs7941399 (0.5), rs35805829 (0.5), rs10838698 (0.5), rs7126378 (0.5), rs10838751 (0.49), rs11039379 (0.49), rs11602395 (0.49), rs11039329 (0.48), rs6485753 (0.48), rs9633830 (0.48), rs10838768 (0.47), rs7947730 (0.47), rs896815 (0.47), rs4752881 (0.46), rs10838740 (0.46), rs10742824 (0.46), rs7936948 (0.45), rs7945312 (0.45), rs10838763 (0.45), rs113211479 (0.44), rs55876153 (0.44), rs12794570 (0.44), rs11039405 (0.44), rs7121038 (0.43), rs12798346 (0.43), rs11039389 (0.43), rs10838724 (0.42), rs3817334 (0.42), rs4752865 (0.42), rs12225854 (0.41), rs10838753 (0.41), rs11039426 (0.41), rs35496532 (0.41), rs11604825 (0.4), rs4752845 (0.4), rs10838776 (0.4), rs10838767 (0.39), rs7117115 (0.39), rs35902101 (0.39), rs11039414 (0.38), rs35985502 (0.38), rs34128973 (0.38), rs7116970 (0.38), rs12361031 (0.37), rs3740686 (0.37), rs11605348 (0.37), rs11039255 (0.36), rs10742817 (0.36), rs10838782 (0.36), rs7124396 (0.35), rs12226590 (0.35), rs3781626 (0.35), rs2242081 (0.35), rs11039373 (0.34), rs9909 (0.34), rs12419692 (0.34), rs12787330 (0.33), rs35705029 (0.33), rs10838736 (0.33), rs60075622 (0.32), rs7102205 (0.32), rs66749409 (0.32), rs10742803 (0.32), rs67116452 (0.31), rs11039354 (0.31), rs10769299 (0.31), rs11039401 (0.3), rs6485763 (0.3), rs7115228 (0.3), rs1563821 (0.29), rs7107922 (0.29), rs12226431 (0.29), rs11039432 (0.29), rs11039419 (0.28), rs11039415 (0.28), rs7933896 (0.28), rs11039390 (0.27), rs7929014 (0.27), rs10838739 (0.27), rs4752994 (0.26), rs10769258 (0.26), rs2856661 (0.26), rs7114813 (0.26), rs7944084 (0.25), rs4752993 (0.25), rs11039433 (0.25), rs2053980 (0.24), rs12787646 (0.24), rs12799623 (0.24), rs10769262 (0.23), rs10838773 (0.23), rs28498823 (0.23), rs2868459 (0.23), rs11039342 (0.22), rs11039345 (0.22), rs7112248 (0.22), rs12363232 (0.21), rs10838741 (0.21), rs2290851 (0.21), rs55927797 (0.2), rs77595962 (0.2), rs11039200 (0.2), rs6485775 (0.2), rs7116320 (0.19), rs11039434 (0.19), rs4752990 (0.19), rs11039392 (0.18), rs2293578 (0.18), rs7928842 (0.18), rs7935521 (0.17), rs3817335 (0.17), rs10838760 (0.17), rs10838754 (0.17), rs2071304 (0.16), rs7122217 (0.16), rs11039327 (0.16), rs34923397 (0.15), rs4539273 (0.15), rs11039337 (0.15), rs7928163 (0.14), rs10458915 (0.14), rs11039336 (0.14), rs10838777 (0.14), rs7107726 (0.13), rs12421563 (0.13), rs4992357 (0.13), rs34958982 (0.12), rs7111606 (0.12), rs7940536 (0.12), rs7942074 (0.11), rs7120413 (0.11), rs10838697 (0.11), rs11039376 (0.11), rs7122182 (0.1), rs56400411 (0.1), rs11039431 (0.1), rs4752792 (0.09), rs181677220 (0.09), rs3886262 (0.09), rs7925211 (0.08), rs7935528 (0.08), rs11823949 (0.08), rs12364432 (0.08), rs7129237 (0.07), rs7103013 (0.07), rs4752798 (0.07), rs375314312 (0.06), rs11039429 (0.06), rs1317149 (0.06), rs10838758 (0.05), rs7111957 (0.05), rs2071305 (0.05), rs7118747 (0.05), rs59360790 (0.04), rs61895760 (0.04), rs4752999 (0.04), rs34614231 (0.03), rs10838704 (0.03), rs4752827 (0.03), rs7948468 (0.02), rs10838746 (0.02), rs896816 (0.02), rs7111576 (0.02), rs55677087 (0.01), rs4752856 (0.01), rs12798028 (0.01), rs17788930 (0)
rs1107850	40	rs2090035 (1), rs870527 (0.98), rs11693335 (0.95), rs10495703 (0.93), rs11687133 (0.9), rs2090032 (0.88), rs2090034 (0.85), rs10495702 (0.83), rs870526 (0.8), rs907867 (0.78), rs7584270 (0.76), rs6531216 (0.73), rs13009332 (0.71), rs1473886 (0.68), rs6728072 (0.66), rs907869 (0.63), rs977130 (0.61), rs35633876 (0.59), rs79279183 (0.56), rs7569900 (0.54), rs6531214 (0.51), rs11096640 (0.49), rs11899121 (0.46), rs1107851 (0.44),

		rs35135293 (0.41), rs2881925 (0.39), rs11895352 (0.37), rs13010903 (0.34), rs907866 (0.32), rs7577335 (0.29), rs907872 (0.27), rs7600854 (0.22), rs11096641 (0.2), rs2137718 (0.17), rs12478327 (0.15), rs57096893 (0.12), rs907868 (0.1), rs11096646 (0.07), rs11687037 (0.05), rs11894335 (0.02)
rs1109166	338	rs255049 (1), rs12444968 (1), rs112894876 (0.99), rs73597580 (0.99), rs117751972 (0.99), rs1074143 (0.99), rs75909028 (0.98), rs113704872 (0.98), rs73597581 (0.98), rs76595854 (0.97), rs77884900 (0.97), rs28428518 (0.97), rs7206671 (0.96), rs73594554 (0.96), rs12448227 (0.96), rs12600062 (0.96), rs7194694 (0.95), rs6499143 (0.95), rs1971546 (0.95), rs7203742 (0.94), rs56047901 (0.94), rs78537727 (0.94), rs113258243 (0.94), rs73594556 (0.93), rs255048 (0.93), rs7199443 (0.93), rs1975802 (0.92), rs113493894 (0.92), rs16957696 (0.92), rs2271294 (0.92), rs12599178 (0.91), rs118035472 (0.91), rs113162813 (0.91), rs2271293 (0.9), rs8047978 (0.9), rs7193537 (0.9), rs56219053 (0.89), rs13816 (0.89), rs12445533 (0.89), rs7192187 (0.89), rs8056649 (0.88), rs141991634 (0.88), rs12599238 (0.88), rs7199699 (0.87), rs8054091 (0.87), rs150850722 (0.87), rs12600144 (0.87), rs73612690 (0.86), rs13336189 (0.86), rs4474673 (0.86), rs11043 (0.85), rs9932251 (0.85), rs55661503 (0.85), rs2290700 (0.85), rs8046355 (0.84), rs8064114 (0.84), rs140979755 (0.84), rs12447931 (0.83), rs2003099 (0.83), rs9934328 (0.83), rs111430913 (0.82), rs11864819 (0.82), rs73599521 (0.82), rs113896227 (0.82), rs111721260 (0.81), rs111632263 (0.81), rs1476306 (0.81), rs56377113 (0.8), rs255052 (0.8), rs76495028 (0.8), rs12600199 (0.8), rs111465361 (0.79), rs28508024 (0.79), rs7196789 (0.79), rs76419201 (0.78), rs9932414 (0.78), rs113790673 (0.78), rs10500543 (0.77), rs75450962 (0.77), rs2292318 (0.77), rs2418739 (0.77), rs112556034 (0.76), rs8061500 (0.76), rs200054031 (0.76), rs56303487 (0.75), rs28587148 (0.75), rs56230350 (0.75), rs28548264 (0.75), rs12448677 (0.74), rs3809630 (0.74), rs9931987 (0.74), rs75735284 (0.73), rs8048365 (0.73), rs73591961 (0.73), rs7198357 (0.73), rs7191129 (0.72), rs73597578 (0.72), rs75229939 (0.72), rs8047119 (0.71), rs56097211 (0.71), rs55897859 (0.71), rs12598274 (0.7), rs186642615 (0.7), rs73612208 (0.7), rs6499163 (0.7), rs12446007 (0.69), rs73591976 (0.69), rs80346622 (0.69), rs9889207 (0.68), rs56076526 (0.68), rs112529313 (0.68), rs7189794 (0.68), rs8046975 (0.67), rs76841123 (0.67), rs57408712 (0.67), rs12447640 (0.66), rs112192397 (0.66), rs7189155 (0.66), rs8058690 (0.65), rs1124993 (0.65), rs4567701 (0.65), rs12446667 (0.65), rs9922934 (0.64), rs9746872 (0.64), rs1125332 (0.64), rs8044328 (0.63), rs11075669 (0.63), rs3826164 (0.63), rs8047343 (0.63), rs111227007 (0.62), rs111315946 (0.62), rs12445396 (0.62), rs7187202 (0.61), rs8051587 (0.61), rs6499157 (0.61), rs4640173 (0.61), rs56071820 (0.6), rs55781197 (0.6), rs11075665 (0.6), rs139784051 (0.59), rs7190079 (0.59), rs6499161 (0.59), rs9928783 (0.58), rs3785109 (0.58), rs113563886 (0.58), rs8057986 (0.58), rs147667023 (0.57), rs9934405 (0.57), rs12598630 (0.57), rs10852439 (0.56), rs112900803 (0.56), rs10468274 (0.56), rs9923710 (0.56), rs73591955 (0.55), rs12599880 (0.55), rs59600980 (0.55), rs7187289 (0.54), rs1124324 (0.54), rs67114979 (0.54), rs146250480 (0.54), rs2285910 (0.53), rs8048034 (0.53), rs2008173 (0.53), rs2418736 (0.52), rs9939821 (0.52), rs36086450 (0.52), rs76836953 (0.51), rs112099181 (0.51), rs2418738 (0.51), rs7193701 (0.51), rs56138523 (0.5), rs111934528 (0.5), rs13334205 (0.5), rs8046907 (0.49), rs6499162 (0.49), rs58588228 (0.49), rs113502835 (0.48), rs8044995 (0.48), rs76984161 (0.48), rs79323429 (0.47), rs113505570 (0.47), rs8060967 (0.47), rs3785100

		(0.46), rs4359427 (0.46), rs6499134 (0.46), rs7199487 (0.46), rs13334918 (0.45), rs191996617 (0.45), rs113579235 (0.45), rs7201591 (0.44), rs112086775 (0.44), rs1134760 (0.44), rs6499145 (0.44), rs73612222 (0.43), rs76501820 (0.43), rs12448991 (0.43), rs35432211 (0.42), rs3785108 (0.42), rs7204208 (0.42), rs117761434 (0.42), rs255054 (0.41), rs20549 (0.41), rs55757502 (0.41), rs7193935 (0.4), rs7195605 (0.4), rs79577483 (0.4), rs35723182 (0.39), rs11075670 (0.39), rs3815173 (0.39), rs76053508 (0.38), rs113201209 (0.38), rs73599506 (0.38), rs73593810 (0.37), rs74629553 (0.37), rs7205935 (0.37), rs7188085 (0.37), rs12448923 (0.36), rs8060686 (0.36), rs3743739 (0.35), rs36046686 (0.35), rs9922594 (0.35), rs8057119 (0.35), rs255053 (0.34), rs2292316 (0.34), rs12446198 (0.34), rs56070533 (0.33), rs16942887 (0.33), rs16957489 (0.33), rs2418735 (0.32), rs7188900 (0.32), rs9928653 (0.32), rs12445876 (0.32), rs7194894 (0.31), rs112434994 (0.31), rs6499160 (0.31), rs9888986 (0.3), rs79375669 (0.3), rs72556537 (0.3), rs58388686 (0.3), rs73612297 (0.29), rs113731015 (0.29), rs111820418 (0.29), rs12444313 (0.28), rs7184821 (0.28), rs1125333 (0.28), rs113066419 (0.27), rs78229587 (0.27), rs9938020 (0.27), rs12598256 (0.27), rs8057577 (0.26), rs73597582 (0.26), rs111967459 (0.26), rs7185308 (0.25), rs9928531 (0.25), rs75297764 (0.25), rs59708959 (0.25), rs7195172 (0.24), rs28563117 (0.24), rs9938553 (0.24), rs8048364 (0.23), rs74024145 (0.23), rs7189381 (0.23), rs8045898 (0.23), rs8044558 (0.22), rs56374641 (0.22), rs60990105 (0.22), rs12596883 (0.21), rs73591968 (0.21), rs8044014 (0.21), rs1985437 (0.2), rs12444188 (0.2), rs8182201 (0.2), rs111402974 (0.2), rs56845922 (0.19), rs7197726 (0.19), rs112310696 (0.19), rs55682260 (0.18), rs75796305 (0.18), rs11553287 (0.18), rs113804408 (0.18), rs193192671 (0.17), rs12597573 (0.17), rs118005841 (0.17), rs111948678 (0.16), rs12447119 (0.16), rs111701446 (0.16), rs7204192 (0.15), rs116821144 (0.15), rs73612697 (0.15), rs79719939 (0.15), rs55790290 (0.14), rs7187476 (0.14), rs7199878 (0.14), rs7206792 (0.13), rs7204974 (0.13), rs77996485 (0.13), rs145158766 (0.13), rs8059305 (0.12), rs1125331 (0.12), rs60626639 (0.12), rs12447080 (0.11), rs111606836 (0.11), rs112358753 (0.11), rs76646352 (0.11), rs13339471 (0.1), rs9923003 (0.1), rs17688076 (0.1), rs9931366 (0.09), rs7199588 (0.09), rs3743735 (0.09), rs9922165 (0.08), rs9674089 (0.08), rs112002856 (0.08), rs28700885 (0.08), rs60638792 (0.07), rs7201742 (0.07), rs61421564 (0.07), rs9674047 (0.06), rs148537303 (0.06), rs7197756 (0.06), rs28465467 (0.06), rs60769312 (0.05), rs55699031 (0.05), rs7196076 (0.05), rs2418737 (0.04), rs55725810 (0.04), rs12449157 (0.04), rs75286242 (0.04), rs8060893 (0.03), rs2107369 (0.03), rs73594581 (0.03), rs2290699 (0.02), rs73598006 (0.02), rs4517790 (0.02), rs8059575 (0.01), rs255056 (0.01), rs11555011 (0.01), rs3785098 (0)
rs11133338	58	rs3819387 (1), rs7694422 (0.98), rs12509022 (0.97), rs35441406 (0.95), rs34284864 (0.93), rs1005438 (0.92), rs7684939 (0.9), rs2017472 (0.88), rs4241987 (0.86), rs2282781 (0.85), rs2237035 (0.83), rs11730497 (0.81), rs7675203 (0.8), rs13119906 (0.78), rs2282779 (0.76), rs2159935 (0.75), rs11736016 (0.73), rs759082 (0.71), rs2703456 (0.69), rs17827760 (0.68), rs6554196 (0.66), rs759083 (0.64), rs4864915 (0.63), rs3756197 (0.61), rs12512899 (0.59), rs7665611 (0.58), rs3819389 (0.56), rs7656858 (0.54), rs7663318 (0.53), rs13147869 (0.51), rs71599647 (0.49), rs7655588 (0.47), rs7684000 (0.46), rs57280936 (0.44), rs2215726 (0.42), rs7655556 (0.41), rs13128858 (0.39), rs7674998 (0.37), rs12498193 (0.36), rs11733357

		(0.34), rs2237031 (0.32), rs34269143 (0.31), rs7656845 (0.29), rs17827974 (0.27), rs6825657 (0.25), rs13135792 (0.24), rs12503461 (0.22), rs2282780 (0.2), rs2282778 (0.19), rs13121174 (0.17), rs6851498 (0.14), rs2237026 (0.12), rs2237028 (0.1), rs113647785 (0.08), rs12504244 (0.07), rs13138563 (0.05), rs6831020 (0.03), rs3819392 (0.02)
rs11248061	47	rs62294486 (1), rs3796622 (0.98), rs3755963 (0.96), rs4690219 (0.94), rs4690220 (0.92), rs6814642 (0.9), rs3775120 (0.88), rs4690326 (0.85), rs73209867 (0.81), rs4690221 (0.79), rs6842493 (0.77), rs7697111 (0.75), rs2290403 (0.73), rs10016294 (0.71), rs3775113 (0.69), rs1051613 (0.67), rs13137144 (0.65), rs11734449 (0.63), rs1377587 (0.6), rs3733346 (0.58), rs13101828 (0.56), rs3775114 (0.54), rs10902762 (0.52), rs11729289 (0.5), rs1377586 (0.48), rs4690229 (0.46), rs935977 (0.44), rs12510382 (0.42), rs10008187 (0.4), rs11724804 (0.38), rs6851795 (0.35), rs3733349 (0.33), rs3796621 (0.31), rs113075206 (0.29), rs112333492 (0.27), rs4690196 (0.25), rs11724898 (0.23), rs873785 (0.21), rs111344337 (0.19), rs3775121 (0.17), rs28504033 (0.15), rs62297086 (0.13), rs9799792 (0.1), rs73209865 (0.08), rs3733345 (0.06), rs6848199 (0.04), rs6813110 (0.02)
rs113629348	1	rs143102005 (0.5)
rs117254618	4	rs117522749 (0.8), rs36049418 (0.6), rs78897229 (0.4), rs74845765 (0.2)
rs117323391	8	rs75689126 (1), rs118166373 (0.78), rs80281960 (0.67), rs141057320 (0.56), rs117700123 (0.44), rs143858063 (0.33), rs75242419 (0.22), rs117953956 (0.11)
rs11755266	8	rs34523546 (1), rs112563428 (0.89), rs3822921 (0.78), rs75398113 (0.67), rs36018387 (0.56), rs13205738 (0.44), rs3800406 (0.22), rs114863007 (0.11)
rs144025261	17	rs15052 (1), rs73043273 (0.94), rs73045223 (0.89), rs56254331 (0.83), rs139833223 (0.78), rs73045231 (0.72), rs12461203 (0.67), rs4803447 (0.61), rs55841050 (0.56), rs73045226 (0.5), rs73045269 (0.44), rs55994820 (0.39), rs8109440 (0.33), rs17251589 (0.28), rs12459929 (0.22), rs12459996 (0.17), rs137991784 (0.11)
rs1515114	116	rs2943632 (1), rs2713542 (0.99), rs1515110 (0.98), rs2943660 (0.97), rs2222018 (0.97), rs2713544 (0.96), rs1399623 (0.95), rs6706153 (0.94), rs2176042 (0.93), rs2673142 (0.92), rs1399626 (0.92), rs2972144 (0.91), rs2138157 (0.9), rs2673137 (0.89), rs2713551 (0.88), rs2673141 (0.87), rs952227 (0.86), rs2943644 (0.86), rs2713560 (0.85), rs2894585 (0.84), rs2943646 (0.83), rs1515100 (0.82), rs2713549 (0.81), rs908252 (0.81), rs2673131 (0.8), rs2972156 (0.79), rs2943650 (0.77), rs4645008 (0.76), rs1515108 (0.75), rs2943653 (0.75), rs2943636 (0.74), rs2138161 (0.73), rs6757213 (0.72), rs201810030 (0.71), rs2713558 (0.7), rs2972146 (0.69), rs13423088 (0.69), rs2673134 (0.68), rs1399625 (0.67), rs114562459 (0.66), rs114616721 (0.65), rs2673128 (0.64), rs1515104 (0.64), rs2673140 (0.63), rs1515099 (0.62), rs1515116 (0.61), rs2673136 (0.6), rs2943647 (0.59), rs2972157 (0.58), rs2972158 (0.58), rs2396315 (0.57), rs1399624 (0.56), rs2943637 (0.55), rs2943645 (0.54), rs1515098 (0.53), rs2972154 (0.53), rs2458993 (0.52), rs1024137 (0.51), rs2713541 (0.5), rs2943641 (0.49), rs2972137 (0.48), rs2972143 (0.47), rs2943654 (0.47), rs2943651

		(0.46), rs2176040 (0.45), rs2396314 (0.44), rs2713539 (0.43), rs6436620 (0.42), rs10439362 (0.42), rs35787079 (0.41), rs2943658 (0.4), rs2943652 (0.39), rs2943649 (0.38), rs2972135 (0.37), rs1567973 (0.36), rs4072096 (0.36), rs2894586 (0.35), rs2943659 (0.34), rs2972150 (0.33), rs2203452 (0.32), rs2713536 (0.31), rs2396316 (0.31), rs1606791 (0.3), rs2943635 (0.29), rs62188784 (0.28), rs2713556 (0.26), rs2943640 (0.25), rs925735 (0.25), rs2713553 (0.24), rs2713540 (0.23), rs2972152 (0.22), rs2943633 (0.21), rs1913657 (0.2), rs2713548 (0.19), rs6713510 (0.19), rs1515102 (0.18), rs2943656 (0.17), rs2972140 (0.16), rs2713559 (0.15), rs2972136 (0.14), rs2972145 (0.14), rs537433434 (0.13), rs6717669 (0.12), rs2972155 (0.11), rs2673144 (0.1), rs1813831 (0.09), rs2943648 (0.08), rs2972138 (0.08), rs2396312 (0.07), rs2713557 (0.06), rs2972153 (0.05), rs2972149 (0.04), rs1399627 (0.03), rs2972147 (0.03), rs2673135 (0.02), rs2943657 (0.01)
rs17153747	57	rs117614588 (1), rs8191605 (0.98), rs4840585 (0.97), rs3757949 (0.95), rs55848834 (0.93), rs4124646 (0.91), rs8191606 (0.9), rs17810696 (0.88), rs73207245 (0.86), rs2645398 (0.84), rs8191625 (0.83), rs10503425 (0.81), rs4841593 (0.79), rs8191560 (0.78), rs3729856 (0.76), rs56187811 (0.74), rs56213203 (0.72), rs8191676 (0.71), rs4841589 (0.69), rs8191608 (0.67), rs73207237 (0.66), rs8191607 (0.64), rs73207239 (0.62), rs55757177 (0.6), rs56303605 (0.59), rs8191558 (0.57), rs73207216 (0.55), rs904006 (0.53), rs8191617 (0.52), rs2740431 (0.5), rs8191538 (0.48), rs73205195 (0.47), rs2686188 (0.45), rs55658394 (0.43), rs804284 (0.41), rs4841586 (0.4), rs55877487 (0.38), rs73207235 (0.36), rs4841584 (0.34), rs17153752 (0.33), rs28628715 (0.31), rs56043655 (0.29), rs17153785 (0.28), rs4841585 (0.26), rs116513362 (0.24), rs59431377 (0.21), rs4840584 (0.19), rs8191556 (0.17), rs4840582 (0.16), rs66535756 (0.14), rs28709984 (0.12), rs55650299 (0.1), rs73207248 (0.09), rs28626371 (0.07), rs17810775 (0.05), rs73207246 (0.03), rs376938321 (0.02)
rs17561950	72	rs8075131 (1), rs62079153 (0.99), rs9900564 (0.97), rs8071889 (0.96), rs4969141 (0.95), rs4969143 (0.93), rs11077362 (0.92), rs1976704 (0.9), rs11653383 (0.89), rs3744217 (0.88), rs12944051 (0.86), rs3817294 (0.85), rs11657987 (0.84), rs2292642 (0.82), rs16971107 (0.81), rs11653305 (0.79), rs3817295 (0.78), rs12453598 (0.77), rs8072632 (0.75), rs4082919 (0.74), rs4969186 (0.73), rs11077364 (0.71), rs4969179 (0.7), rs12452576 (0.68), rs2376583 (0.67), rs12451109 (0.66), rs3826323 (0.64), rs12601079 (0.63), rs55938653 (0.62), rs12450528 (0.6), rs7225449 (0.59), rs4969183 (0.58), rs12450500 (0.56), rs938350 (0.55), rs938351 (0.53), rs2292640 (0.52), rs7208596 (0.51), rs16971105 (0.49), rs11656568 (0.48), rs7212201 (0.47), rs11657865 (0.45), rs8077859 (0.44), rs6501208 (0.42), rs7219625 (0.41), rs12451791 (0.4), rs2292643 (0.38), rs12602206 (0.37), rs4969184 (0.36), rs4129767 (0.34), rs938348 (0.33), rs2292644 (0.32), rs4580257 (0.3), rs4490057 (0.29), rs12951460 (0.27), rs11657860 (0.26), rs4969187 (0.25), rs11077363 (0.23), rs12948394 (0.22), rs4969182 (0.19), rs1976703 (0.18), rs4969178 (0.16), rs4969145 (0.15), rs55633207 (0.14), rs4366775 (0.12), rs7207782 (0.11), rs12451715 (0.1), rs11077361 (0.08), rs2376584 (0.07), rs3744219 (0.05), rs7215542 (0.04), rs56658017 (0.03), rs4969142 (0.01)
rs17717355	7	rs75670664 (1), rs74710540 (0.88), rs79067723 (0.75), rs188584068 (0.5), rs75120785 (0.38), rs76222503 (0.25), rs76024223 (0.13)

rs2058914	279	rs12900944 (1), rs62020789 (1), rs2899695 (0.99), rs544334138 (0.99), rs11634705 (0.99), rs6494407 (0.98), rs62020761 (0.98), rs62014166 (0.98), rs11639343 (0.97), rs12102213 (0.97), rs12324588 (0.96), rs144877098 (0.96), rs62011298 (0.96), rs62012820 (0.95), rs78029984 (0.95), rs9972484 (0.95), rs62012775 (0.94), rs62012770 (0.94), rs67566652 (0.94), rs11071764 (0.93), rs11632919 (0.93), rs62012773 (0.93), rs62014170 (0.92), rs7171858 (0.92), rs62012824 (0.91), rs7169217 (0.91), rs56163012 (0.91), rs2099920 (0.9), rs4344682 (0.9), rs12148623 (0.9), rs11635129 (0.89), rs978363 (0.89), rs10851732 (0.89), rs12324645 (0.88), rs1011258 (0.88), rs17185673 (0.88), rs13379670 (0.87), rs62014216 (0.87), rs10163125 (0.87), rs17773700 (0.86), rs7178762 (0.86), rs62014183 (0.85), rs7172896 (0.85), rs4412925 (0.85), rs28438975 (0.84), rs3210904 (0.84), rs62012827 (0.84), rs16947268 (0.83), rs12324009 (0.83), rs62011343 (0.83), rs2271241 (0.82), rs2089619 (0.82), rs11635117 (0.82), rs11071759 (0.81), rs8037694 (0.81), rs978362 (0.8), rs62011335 (0.8), rs9972479 (0.8), rs62012836 (0.79), rs11630289 (0.79), rs16947343 (0.79), rs11635472 (0.78), rs74020820 (0.78), rs79566801 (0.78), rs62014182 (0.77), rs2202867 (0.77), rs35360755 (0.77), rs11639412 (0.76), rs62014174 (0.76), rs17765642 (0.76), rs12899671 (0.75), rs35627589 (0.75), rs62011342 (0.74), rs62020781 (0.74), rs4074448 (0.74), rs4536410 (0.73), rs11633830 (0.73), rs17184782 (0.73), rs11632718 (0.72), rs62012772 (0.72), rs2228510 (0.72), rs8034675 (0.71), rs62014214 (0.71), rs62022639 (0.71), rs62011301 (0.7), rs2228517 (0.7), rs80275182 (0.7), rs2120252 (0.69), rs146159842 (0.69), rs11629641 (0.68), rs12324720 (0.68), rs11854824 (0.68), rs4984251 (0.67), rs11071758 (0.67), rs62011330 (0.67), rs17184592 (0.66), rs11857705 (0.66), rs62014165 (0.66), rs11071770 (0.65), rs62011325 (0.65), rs62012818 (0.65), rs8025240 (0.64), rs62011333 (0.64), rs28496317 (0.63), rs4984249 (0.63), rs11636604 (0.63), rs2063281 (0.62), rs7176089 (0.62), rs62012830 (0.62), rs1131647 (0.61), rs982077 (0.61), rs62012833 (0.61), rs62011346 (0.6), rs4577025 (0.6), rs77912104 (0.6), rs12914183 (0.59), rs28517814 (0.59), rs62014173 (0.59), rs7175717 (0.58), rs10152714 (0.58), rs2175088 (0.57), rs112593021 (0.57), rs76453046 (0.57), rs62014171 (0.56), rs2099922 (0.56), rs12913540 (0.55), rs17184753 (0.55), rs11636758 (0.55), rs62012834 (0.54), rs62014169 (0.54), rs8040757 (0.54), rs62011326 (0.53), rs79730824 (0.53), rs1396713 (0.52), rs34726252 (0.52), rs17186681 (0.52), rs12911939 (0.51), rs62014163 (0.51), rs62012838 (0.51), rs12708478 (0.5), rs9972569 (0.5), rs79714693 (0.5), rs8036500 (0.49), rs62012766 (0.49), rs62012837 (0.49), rs10519213 (0.48), rs62011302 (0.48), rs11631837 (0.48), rs9972554 (0.47), rs17186513 (0.47), rs12148660 (0.46), rs1134684 (0.46), rs62012774 (0.46), rs60618359 (0.45), rs80047978 (0.45), rs77715851 (0.45), rs12912296 (0.44), rs77064122 (0.44), rs11857787 (0.44), rs12903326 (0.43), rs7164926 (0.43), rs78857043 (0.43), rs7180823 (0.42), rs62014167 (0.42), rs143267946 (0.41), rs11457 (0.41), rs35216087 (0.41), rs7180729 (0.4), rs11631176 (0.4), rs7177766 (0.4), rs4984296 (0.39), rs34675318 (0.39), rs4073096 (0.39), rs8034342 (0.38), rs62012822 (0.38), rs62012831 (0.38), rs62012786 (0.37), rs62014172 (0.37), rs76836538 (0.37), rs28661116 (0.36), rs117737827 (0.36), rs62012825 (0.35), rs11630720 (0.35), rs62012365 (0.35), rs55941923 (0.34), rs17185287 (0.34), rs35943131 (0.34), rs57932859 (0.33), rs16947468 (0.33), rs79076440 (0.33), rs62011331 (0.32), rs79976004 (0.32), rs62012829 (0.32), rs11071771 (0.31), rs3934552 (0.31), rs2058913 (0.3), rs10152669 (0.3), rs55640532 (0.3), rs576907047

		(0.29), rs2053594 (0.29), rs62014213 (0.29), rs10519223 (0.28), rs62011297 (0.28), rs62014215 (0.28), rs12372939 (0.27), rs9972527 (0.27), rs60779579 (0.27), rs17185876 (0.26), rs62023078 (0.26), rs62014217 (0.26), rs8037046 (0.25), rs4984307 (0.25), rs62012769 (0.24), rs11855988 (0.24), rs112305294 (0.24), rs17772465 (0.23), rs3764186 (0.23), rs62014185 (0.23), rs62011344 (0.22), rs12592616 (0.22), rs74018158 (0.22), rs17186555 (0.21), rs4984313 (0.21), rs62012364 (0.2), rs1470314 (0.2), rs62012782 (0.2), rs11631436 (0.19), rs76115381 (0.19), rs28627799 (0.18), rs62023114 (0.18), rs62014164 (0.18), rs8035561 (0.17), rs17184999 (0.17), rs34562625 (0.17), rs10152687 (0.16), rs62014176 (0.16), rs10152174 (0.16), rs17773244 (0.15), rs34143602 (0.15), rs10438347 (0.14), rs10438289 (0.14), rs12907238 (0.13), rs11635351 (0.13), rs12102137 (0.13), rs62011345 (0.12), rs13379688 (0.12), rs62012787 (0.12), rs10162809 (0.11), rs60954093 (0.11), rs62012768 (0.11), rs146311723 (0.1), rs17766223 (0.1), rs4984253 (0.1), rs62020829 (0.09), rs533785201 (0.09), rs4984303 (0.09), rs8040381 (0.08), rs11633180 (0.08), rs34479262 (0.07), rs4984305 (0.07), rs62012839 (0.07), rs12324419 (0.06), rs57723091 (0.06), rs11636351 (0.06), rs10152416 (0.05), rs77107306 (0.05), rs56175545 (0.05), rs17766325 (0.04), rs17766277 (0.04), rs4984297 (0.04), rs12900621 (0.03), rs62012826 (0.03), rs62014184 (0.02), rs61059171 (0.02), rs7173984 (0.02), rs7180358 (0.01), rs62012765 (0.01), rs62012823 (0.01), rs75663391 (0)
rs2232170	172	rs2413035 (1), rs2283879 (0.99), rs5749270 (0.99), rs5753521 (0.98), rs3208937 (0.98), rs5997902 (0.97), rs4820948 (0.97), rs5997949 (0.96), rs1858821 (0.95), rs5994394 (0.95), rs5753631 (0.94), rs5753486 (0.94), rs9609268 (0.93), rs9609267 (0.93), rs8135344 (0.92), rs2899164 (0.91), rs4820970 (0.91), rs2413034 (0.9), rs5749222 (0.9), rs5749227 (0.89), rs7285619 (0.89), rs8135347 (0.88), rs9606846 (0.87), rs9609266 (0.87), rs6518737 (0.86), rs9606833 (0.86), rs5749291 (0.85), rs13054220 (0.84), rs7292168 (0.84), rs9606839 (0.83), rs5753507 (0.83), rs12152166 (0.82), rs9606848 (0.82), rs2413031 (0.81), rs4820953 (0.8), rs71324184 (0.8), rs5753601 (0.79), rs5997897 (0.79), rs5753669 (0.78), rs7287267 (0.78), rs4820946 (0.77), rs5753666 (0.76), rs5753505 (0.76), rs4820963 (0.75), rs9609302 (0.75), rs8139160 (0.74), rs5749272 (0.74), rs8142121 (0.73), rs5749267 (0.72), rs5753623 (0.72), rs9609304 (0.71), rs5749279 (0.71), rs9606831 (0.7), rs5749243 (0.7), rs8138596 (0.69), rs2413052 (0.68), rs4603880 (0.68), rs9609329 (0.67), rs5749283 (0.67), rs5749268 (0.66), rs9606830 (0.66), rs1034588 (0.65), rs5753632 (0.64), rs5753597 (0.64), rs8139625 (0.63), rs715510 (0.63), rs5749266 (0.62), rs11913992 (0.61), rs9609303 (0.61), rs28873672 (0.6), rs5753610 (0.6), rs5997913 (0.59), rs5753665 (0.59), rs9609297 (0.58), rs2040533 (0.57), rs5753470 (0.57), rs5753483 (0.56), rs28838399 (0.56), rs5749248 (0.55), rs5753596 (0.55), rs5753639 (0.54), rs9609257 (0.53), rs2073857 (0.53), rs1985725 (0.52), rs5998058 (0.52), rs1468411 (0.51), rs9609277 (0.51), rs2283881 (0.5), rs2413049 (0.49), rs34538851 (0.48), rs5997899 (0.48), rs7293171 (0.47), rs9609254 (0.47), rs2413047 (0.46), rs2078803 (0.45), rs5753613 (0.45), rs4820951 (0.44), rs9609299 (0.44), rs9609265 (0.43), rs737887 (0.43), rs5997893 (0.42), rs5997969 (0.41), rs2273249 (0.41), rs5997896 (0.4), rs9606847 (0.4), rs2413033 (0.39), rs2079431 (0.39), rs5753664 (0.38), rs714909 (0.37), rs5753648 (0.37), rs1001599 (0.36), rs9609295 (0.36), rs4820043 (0.35), rs13158 (0.34), rs9609301 (0.34), rs5749229 (0.33), rs34545960 (0.33), rs4141404 (0.32), rs2413029 (0.32), rs2228619 (0.31),

		rs5753671 (0.3), rs9606843 (0.3), rs5753698 (0.29), rs5749286 (0.28), rs9621211 (0.28), rs695438 (0.27), rs5753659 (0.26), rs2232176 (0.26), rs13057504 (0.25), rs5753500 (0.25), rs5749265 (0.24), rs5997939 (0.24), rs5753609 (0.23), rs5753612 (0.22), rs9609276 (0.22), rs5753615 (0.21), rs2413032 (0.21), rs8141150 (0.2), rs5753543 (0.2), rs2413045 (0.19), rs9621210 (0.18), rs9606852 (0.18), rs2092829 (0.17), rs9609296 (0.17), rs5753628 (0.16), rs4299418 (0.16), rs737684 (0.15), rs7284622 (0.14), rs5753661 (0.14), rs59315359 (0.13), rs5997977 (0.13), rs5997933 (0.12), rs5749295 (0.11), rs5753621 (0.11), rs1034589 (0.1), rs2413046 (0.1), rs2106294 (0.09), rs9621235 (0.09), rs8135417 (0.08), rs131205 (0.07), rs737888 (0.07), rs738657 (0.06), rs5749298 (0.06), rs5749224 (0.05), rs9609264 (0.05), rs5753503 (0.04), rs1985531 (0.03), rs9609286 (0.03), rs2413043 (0.02), rs9609271 (0.02), rs4820042 (0.01), rs4820945 (0.01)
rs2302774	135	rs8077456 (1), rs3785549 (0.99), rs12940405 (0.99), rs66826786 (0.98), rs9916158 (0.97), rs1007654 (0.96), rs60134943 (0.96), rs2305481 (0.95), rs12450800 (0.94), rs12451897 (0.93), rs12602854 (0.93), rs4065321 (0.92), rs543563352 (0.91), rs2241245 (0.91), rs3744245 (0.9), rs36071767 (0.89), rs2302777 (0.88), rs11078931 (0.88), rs2227321 (0.87), rs34453912 (0.86), rs11655655 (0.85), rs4433838 (0.85), rs1563103 (0.84), rs7218321 (0.83), rs4065876 (0.82), rs3859191 (0.82), rs12453732 (0.81), rs56326707 (0.8), rs56030650 (0.8), rs9889711 (0.79), rs8073254 (0.78), rs4239225 (0.77), rs3859192 (0.77), rs7209742 (0.76), rs8070444 (0.75), rs12601333 (0.74), rs11078935 (0.74), rs11655264 (0.73), rs8072548 (0.72), rs11654208 (0.72), rs34670005 (0.71), rs3785550 (0.7), rs62068184 (0.69), rs3848395 (0.69), rs8076474 (0.68), rs11658328 (0.67), rs11869855 (0.66), rs6503527 (0.66), rs8078692 (0.65), rs2305482 (0.64), rs12601929 (0.64), rs7212944 (0.63), rs201474001 (0.62), rs11078936 (0.61), rs12453341 (0.6), rs3859189 (0.59), rs2313640 (0.58), rs62068183 (0.58), rs12453334 (0.57), rs34540281 (0.56), rs8068260 (0.55), rs34003767 (0.55), rs62068182 (0.54), rs2241244 (0.53), rs9915252 (0.53), rs3816472 (0.52), rs8081437 (0.51), rs55927420 (0.5), rs3902024 (0.5), rs62068175 (0.49), rs7223318 (0.48), rs12309 (0.47), rs12602513 (0.47), rs56396280 (0.46), rs4458030 (0.45), rs67480438 (0.45), rs8075668 (0.44), rs12450688 (0.43), rs9914973 (0.42), rs7502971 (0.42), rs9889716 (0.41), rs11867302 (0.4), rs10445310 (0.39), rs12453764 (0.39), rs8067817 (0.38), rs7219080 (0.37), rs60137005 (0.36), rs35982947 (0.36), rs62068179 (0.35), rs12602514 (0.34), rs12451547 (0.34), rs62068180 (0.33), rs2001476 (0.32), rs7502233 (0.31), rs4072639 (0.31), rs2227333 (0.3), rs2270401 (0.29), rs8065126 (0.28), rs7207600 (0.28), rs17609240 (0.27), rs9895948 (0.26), rs7218742 (0.26), rs11078930 (0.25), rs25645 (0.24), rs3935281 (0.23), rs9898547 (0.23), rs56410675 (0.22), rs11870683 (0.21), rs8068522 (0.2), rs7222039 (0.2), rs62068174 (0.19), rs35272691 (0.18), rs62068181 (0.17), rs34648856 (0.16), rs12453249 (0.15), rs11652139 (0.15), rs2302776 (0.14), rs12943476 (0.13), rs12453343 (0.12), rs34933056 (0.12), rs28618095 (0.11), rs3087852 (0.1), rs8066582 (0.09), rs62068177 (0.09), rs2227319 (0.08), rs62068178 (0.07), rs3907022 (0.07), rs12939682 (0.06), rs3859187 (0.05), rs60667221 (0.04), rs62068176 (0.04), rs8071037 (0.03), rs34342173 (0.02), rs576295138 (0.01), rs1007655 (0.01)
rs231517	95	rs231529 (1), rs11657681 (0.99), rs231526 (0.98), rs4793040 (0.97), rs231540 (0.96), rs140405783 (0.95), rs34239920 (0.94), rs76557360 (0.93),

		rs231514 (0.92), rs4792921 (0.91), rs231506 (0.9), rs75508812 (0.89), rs7212487 (0.88), rs231520 (0.86), rs231537 (0.85), rs11657450 (0.84), rs231493 (0.83), rs231476 (0.82), rs425135 (0.81), rs12453611 (0.8), rs231524 (0.79), rs186720 (0.78), rs1642611 (0.77), rs75939744 (0.76), rs231528 (0.75), rs4793047 (0.74), rs231522 (0.73), rs231498 (0.72), rs75550043 (0.71), rs231530 (0.7), rs231525 (0.69), rs4793048 (0.68), rs231532 (0.67), rs8068019 (0.66), rs8081396 (0.65), rs124720 (0.64), rs231497 (0.63), rs231508 (0.61), rs231494 (0.6), rs231446 (0.59), rs537165 (0.58), rs231513 (0.57), rs231515 (0.56), rs231505 (0.55), rs124722 (0.54), rs231500 (0.53), rs231511 (0.52), rs76204283 (0.51), rs231501 (0.5), rs231534 (0.49), rs231510 (0.48), rs231539 (0.47), rs75086002 (0.46), rs170766 (0.45), rs231535 (0.44), rs746742 (0.43), rs12453522 (0.42), rs231518 (0.41), rs80053172 (0.4), rs231456 (0.39), rs124719 (0.38), rs231507 (0.36), rs72836567 (0.35), rs8079623 (0.34), rs179586 (0.33), rs7221263 (0.31), rs231538 (0.3), rs231479 (0.29), rs124721 (0.28), rs231543 (0.27), rs11654245 (0.26), rs231512 (0.25), rs53907 (0.24), rs79139991 (0.23), rs231516 (0.22), rs231531 (0.21), rs231496 (0.2), rs139009564 (0.19), rs231495 (0.18), rs231533 (0.17), rs35362475 (0.16), rs231499 (0.15), rs72836564 (0.14), rs537169 (0.13), rs11653808 (0.11), rs231503 (0.1), rs231519 (0.09), rs7209751 (0.08), rs231504 (0.07), rs231527 (0.06), rs150055076 (0.05), rs607000 (0.04), rs6503487 (0.03), rs11654132 (0.02), rs231521 (0.01)
rs241776	45	rs4794828 (1), rs1128162 (0.98), rs7212349 (0.96), rs2027993 (0.93), rs4795433 (0.91), rs11869677 (0.89), rs2073867 (0.87), rs2071379 (0.85), rs171360 (0.83), rs241775 (0.8), rs241791 (0.76), rs10853128 (0.74), rs8081240 (0.72), rs241779 (0.7), rs13469 (0.67), rs7212510 (0.65), rs1128161 (0.63), rs1858837 (0.61), rs8079943 (0.59), rs733914 (0.57), rs9913833 (0.54), rs241780 (0.52), rs967645 (0.5), rs2227736 (0.48), rs241777 (0.46), rs11080054 (0.43), rs3093680 (0.41), rs1007398 (0.39), rs11080055 (0.37), rs2239910 (0.35), rs704 (0.33), rs2239907 (0.3), rs6505079 (0.28), rs4795434 (0.26), rs4795435 (0.24), rs2227735 (0.22), rs9910163 (0.2), rs614877 (0.17), rs12947270 (0.15), rs2239911 (0.13), rs3093692 (0.11), rs2239908 (0.09), rs6505077 (0.07), rs708100 (0.04), rs6505081 (0.02)
rs2434612	109	rs2964484 (1), rs1023666 (0.99), rs1878936 (0.98), rs2946179 (0.97), rs6881996 (0.96), rs1650503 (0.95), rs201769762 (0.95), rs2914219 (0.94), rs2963477 (0.93), rs1428442 (0.92), rs1428445 (0.91), rs2914228 (0.9), rs2964500 (0.89), rs2963478 (0.88), rs1030472 (0.87), rs2963453 (0.86), rs2964503 (0.86), rs2963476 (0.85), rs2419922 (0.84), rs1835014 (0.83), rs1017308 (0.82), rs2963434 (0.81), rs2964498 (0.8), rs1650504 (0.79), rs1651272 (0.78), rs2963474 (0.77), rs1650531 (0.77), rs2914226 (0.76), rs2963479 (0.75), rs1878935 (0.74), rs7729301 (0.73), rs1122249 (0.72), rs2963469 (0.71), rs970916 (0.7), rs2914229 (0.69), rs2901008 (0.68), rs3111141 (0.68), rs2946176 (0.67), rs57371317 (0.65), rs2964497 (0.64), rs1981973 (0.63), rs2963480 (0.62), rs2914225 (0.61), rs2914224 (0.6), rs2963449 (0.59), rs2914220 (0.59), rs2963481 (0.58), rs2964496 (0.57), rs2946162 (0.56), rs2963471 (0.55), rs1650527 (0.54), rs748510 (0.53), rs2963463 (0.52), rs1651274 (0.51), rs1650502 (0.5), rs1651267 (0.5), rs1650506 (0.49), rs890939 (0.48), rs2963433 (0.47), rs1428443 (0.46), rs2419909 (0.45), rs2946163 (0.44), rs6881817 (0.43), rs2946156 (0.42), rs890940 (0.41), rs2419923 (0.41), rs2963462 (0.39), rs1122080 (0.38), rs975630 (0.37), rs2914221 (0.36), rs2963468 (0.35), rs1465573 (0.34),

		rs890944 (0.33), rs890945 (0.32), rs890942 (0.32), rs2963426 (0.31), rs1428444 (0.3), rs2963470 (0.29), rs56153134 (0.28), rs7716662 (0.27), rs34023964 (0.26), rs2946164 (0.25), rs2964481 (0.24), rs2963445 (0.23), rs2162858 (0.23), rs2963475 (0.22), rs1650548 (0.21), rs12520982 (0.2), rs2964502 (0.19), rs2434264 (0.18), rs7735131 (0.17), rs2963451 (0.16), rs2914223 (0.15), rs3934712 (0.14), rs2914222 (0.14), rs890943 (0.13), rs2914233 (0.12), rs2914231 (0.11), rs1651268 (0.1), rs2419921 (0.09), rs2946160 (0.08), rs2964501 (0.07), rs2963472 (0.06), rs1650505 (0.05), rs1651270 (0.05), rs1615199 (0.04), rs7701375 (0.03), rs1651266 (0.02), rs1651269 (0.01)
rs2446192	32	rs485710 (1), rs548872 (0.97), rs2446191 (0.94), rs6889719 (0.91), rs13187960 (0.88), rs7735726 (0.85), rs11750403 (0.82), rs518971 (0.79), rs7721475 (0.76), rs520225 (0.73), rs10051239 (0.7), rs479636 (0.67), rs572041 (0.64), rs517339 (0.61), rs2431213 (0.58), rs1559063 (0.55), rs2161134 (0.52), rs2446190 (0.48), rs7717884 (0.45), rs12188901 (0.42), rs538622 (0.39), rs62387404 (0.36), rs6889973 (0.33), rs550294 (0.3), rs544421 (0.27), rs514627 (0.21), rs13159833 (0.18), rs2302959 (0.15), rs6864748 (0.12), rs508371 (0.09), rs10060067 (0.06), rs486113 (0.03)
rs2489623	91	rs2800710 (1), rs2154167 (0.99), rs2489628 (0.98), rs1936800 (0.97), rs9491698 (0.96), rs4566896 (0.95), rs2800709 (0.93), rs1892171 (0.92), rs4620145 (0.91), rs10457487 (0.9), rs2503322 (0.89), rs7764034 (0.88), rs9491699 (0.87), rs2489630 (0.86), rs2186037 (0.85), rs2489631 (0.84), rs9491701 (0.83), rs2503106 (0.82), rs62438503 (0.8), rs6916318 (0.79), rs2326566 (0.78), rs7745274 (0.77), rs2503326 (0.76), rs2503107 (0.75), rs7766106 (0.74), rs2489625 (0.73), rs13204656 (0.72), rs9491709 (0.71), rs2745352 (0.7), rs9482772 (0.68), rs1936807 (0.67), rs9491706 (0.66), rs9491700 (0.65), rs6914968 (0.64), rs2503109 (0.63), rs2800708 (0.62), rs1936806 (0.61), rs9491710 (0.6), rs4404786 (0.59), rs4318902 (0.58), rs2154168 (0.57), rs9482770 (0.55), rs6915525 (0.54), rs1936802 (0.53), rs9491705 (0.52), rs4382293 (0.51), rs2489626 (0.5), rs9491704 (0.49), rs6932207 (0.48), rs7775715 (0.47), rs9491703 (0.46), rs17054398 (0.43), rs1892172 (0.42), rs9482771 (0.41), rs4509142 (0.4), rs11154386 (0.39), rs2503323 (0.38), rs7744964 (0.37), rs1936801 (0.36), rs9285458 (0.35), rs7741021 (0.34), rs7771990 (0.33), rs3734626 (0.32), rs1936809 (0.3), rs9491696 (0.29), rs719726 (0.28), rs1474696 (0.27), rs2503111 (0.26), rs2745353 (0.25), rs1936805 (0.24), rs9491702 (0.23), rs9968918 (0.22), rs4559104 (0.21), rs6569474 (0.2), rs2503320 (0.18), rs2326565 (0.17), rs9968920 (0.16), rs9482773 (0.15), rs6938763 (0.14), rs4644087 (0.13), rs1967685 (0.12), rs7763784 (0.11), rs9491697 (0.1), rs9482775 (0.09), rs2489629 (0.08), rs2489627 (0.07), rs6919601 (0.05), rs7742668 (0.04), rs6569475 (0.03), rs4424101 (0.02), rs1936789 (0.01)
rs2518026	97	rs1861953 (1), rs74252612 (0.99), rs9891949 (0.98), rs113668968 (0.97), rs11078735 (0.96), rs7221412 (0.95), rs12944041 (0.94), rs11649810 (0.93), rs3027234 (0.92), rs959303 (0.91), rs12946862 (0.9), rs1150 (0.89), rs11651993 (0.88), rs8069739 (0.87), rs28447074 (0.86), rs2289590 (0.85), rs9912892 (0.84), rs11656950 (0.83), rs7220442 (0.82), rs9913189 (0.81), rs7219471 (0.8), rs8064449 (0.79), rs7223859 (0.78), rs4791663 (0.77), rs11655996 (0.76), rs6503091 (0.74), rs56256903 (0.73), rs79654937 (0.72), rs6503089 (0.71), rs4791618 (0.7), rs885747 (0.69), rs4792267 (0.68), rs8078338 (0.67), rs2278636 (0.66), rs117347460 (0.65), rs56346215 (0.64), rs72479723 (0.63), rs3027172 (0.62), rs56408410 (0.61),

		rs1132554 (0.6), rs12943463 (0.59), rs2313286 (0.58), rs2241909 (0.57), rs142230565 (0.56), rs9901632 (0.55), rs3027169 (0.54), rs8067606 (0.53), rs8066513 (0.52), rs113170630 (0.51), rs4792687 (0.5), rs8075427 (0.49), rs11650309 (0.48), rs9914077 (0.47), rs7503353 (0.46), rs2585408 (0.45), rs72835272 (0.44), rs8070063 (0.43), rs74506939 (0.42), rs2289591 (0.41), rs11656359 (0.4), rs3027160 (0.39), rs12938531 (0.38), rs2585399 (0.37), rs78286057 (0.36), rs140388689 (0.35), rs566999984 (0.34), rs9893434 (0.33), rs8066511 (0.32), rs9303226 (0.31), rs6416892 (0.3), rs9894148 (0.29), rs78751541 (0.28), rs142913941 (0.27), rs7224566 (0.24), rs9908995 (0.23), rs11650444 (0.22), rs8075680 (0.21), rs11651453 (0.2), rs9894790 (0.19), rs7502447 (0.18), rs2304909 (0.17), rs12451139 (0.16), rs9910821 (0.15), rs3760516 (0.14), rs2278637 (0.13), rs150491398 (0.12), rs2585398 (0.11), rs3027167 (0.1), rs9899533 (0.09), rs11650094 (0.08), rs6503086 (0.07), rs4791641 (0.06), rs6503085 (0.05), rs1476161 (0.04), rs3027246 (0.03), rs76539190 (0.02), rs67736587 (0.01)
rs267738	21	rs72992015 (1), rs56661631 (0.95), rs75056606 (0.91), rs35427932 (0.86), rs143976390 (0.82), rs78132593 (0.77), rs34593358 (0.73), rs2925739 (0.68), rs198325 (0.64), rs17599629 (0.59), rs607518 (0.55), rs2055975 (0.5), rs2925738 (0.45), rs35782301 (0.41), rs72702521 (0.36), rs267733 (0.32), rs61386199 (0.23), rs66958685 (0.18), rs41305070 (0.14), rs11204676 (0.09), rs267734 (0.05)
rs2792751	61	rs2255141 (1), rs7899096 (0.98), rs10749110 (0.97), rs2251579 (0.95), rs2297991 (0.94), rs10749108 (0.92), rs2803608 (0.9), rs2792744 (0.89), rs4917627 (0.87), rs34820178 (0.85), rs2803606 (0.84), rs10787428 (0.82), rs1807800 (0.81), rs2792743 (0.79), rs7096937 (0.77), rs2255400 (0.76), rs3824627 (0.74), rs2419604 (0.73), rs10736219 (0.71), rs2254537 (0.69), rs2803619 (0.68), rs4917628 (0.66), rs10749113 (0.65), rs7089231 (0.63), rs10787429 (0.61), rs10885314 (0.6), rs2803616 (0.58), rs10787427 (0.56), rs2277207 (0.55), rs7897449 (0.53), rs2254532 (0.52), rs2487294 (0.5), rs2297992 (0.48), rs2792703 (0.47), rs4918722 (0.45), rs7084039 (0.44), rs10749111 (0.42), rs2900942 (0.4), rs2251697 (0.39), rs1129555 (0.37), rs10749112 (0.35), rs58175211 (0.34), rs72836628 (0.32), rs4918720 (0.31), rs6585139 (0.29), rs4918719 (0.27), rs2792735 (0.26), rs6585140 (0.23), rs2803609 (0.21), rs10749109 (0.19), rs5024318 (0.18), rs2792736 (0.16), rs6585138 (0.15), rs3824626 (0.13), rs1986438 (0.11), rs7898897 (0.1), rs2792759 (0.08), rs2803611 (0.06), rs2250802 (0.05), rs2263608 (0.03), rs2803621 (0.02)
rs2877984	415	rs7473 (1), rs4651142 (1), rs2296294 (1), rs6672306 (0.99), rs3768615 (0.99), rs6424889 (0.99), rs2274984 (0.99), rs35782443 (0.98), rs10797804 (0.98), rs12563494 (0.98), rs12034436 (0.98), rs10797842 (0.97), rs10911205 (0.97), rs2245784 (0.97), rs11582514 (0.97), rs10752905 (0.96), rs10752888 (0.96), rs10911218 (0.96), rs4233193 (0.96), rs10911247 (0.95), rs12144261 (0.95), rs12143211 (0.95), rs10797848 (0.95), rs10797838 (0.95), rs3738829 (0.94), rs6668980 (0.94), rs4652760 (0.94), rs6673768 (0.94), rs10752891 (0.93), rs6662239 (0.93), rs10797821 (0.93), rs4652764 (0.93), rs10797847 (0.92), rs12061219 (0.92), rs7415796 (0.92), rs729819 (0.92), rs6665685 (0.91), rs10797844 (0.91), rs5022057 (0.91), rs6678517 (0.91), rs10911240 (0.9), rs12086466 (0.9), rs10911263 (0.9), rs12132262 (0.9), rs12753817 (0.9), rs2093982 (0.89), rs10797852 (0.89), rs2296296 (0.89), rs8179262 (0.89), rs6424890 (0.88), rs3768613 (0.88), rs6674729 (0.88), rs9787073 (0.88), rs9943067 (0.87), rs4454510 (0.87), rs6701400

(0.87), rs20557 (0.87), rs2027075 (0.86), rs10752895 (0.86), rs12126434 (0.86), rs3768618 (0.86), rs4652762 (0.85), rs4487994 (0.85), rs10911223 (0.85), rs4129858 (0.85), rs3768620 (0.85), rs12738377 (0.84), rs3935221 (0.84), rs4651136 (0.84), rs10797839 (0.84), rs4350171 (0.83), rs10737241 (0.83), rs4266836 (0.83), rs12091137 (0.83), rs6424881 (0.82), rs4651134 (0.82), rs2224933 (0.82), rs10797823 (0.82), rs6424888 (0.81), rs10797829 (0.81), rs6691755 (0.81), rs10797815 (0.81), rs10797810 (0.8), rs2182020 (0.8), rs10797813 (0.8), rs10737239 (0.8), rs78950541 (0.8), rs6669796 (0.79), rs3934579 (0.79), rs2296290 (0.79), rs10911267 (0.78), rs10797818 (0.78), rs944971 (0.78), rs6669199 (0.78), rs10911231 (0.77), rs10911190 (0.77), rs34721094 (0.77), rs10797820 (0.77), rs10911228 (0.76), rs11588675 (0.76), rs10911260 (0.76), rs35855069 (0.76), rs10752903 (0.75), rs10911242 (0.75), rs6658501 (0.75), rs12078729 (0.75), rs2147585 (0.75), rs10911255 (0.74), rs10797814 (0.74), rs10911213 (0.74), rs6424885 (0.74), rs1360704 (0.73), rs10911233 (0.73), rs10752887 (0.73), rs12071514 (0.73), rs12128323 (0.72), rs10911244 (0.72), rs2296288 (0.72), rs10911258 (0.72), rs4652777 (0.71), rs10911234 (0.71), rs10797806 (0.71), rs3934578 (0.71), rs7542640 (0.7), rs12066082 (0.7), rs10911249 (0.7), rs61081633 (0.7), rs8179282 (0.7), rs4403588 (0.69), rs12146099 (0.69), rs7515822 (0.69), rs10911201 (0.69), rs10797846 (0.68), rs1547715 (0.68), rs6424887 (0.68), rs2093983 (0.68), rs10911198 (0.67), rs3818417 (0.67), rs10797802 (0.67), rs10797800 (0.67), rs7525917 (0.66), rs10797816 (0.66), rs10911200 (0.66), rs1413388 (0.65), rs1413390 (0.65), rs4651135 (0.65), rs34105373 (0.65), rs10911246 (0.65), rs4422969 (0.64), rs10752900 (0.64), rs10797812 (0.64), rs2022392 (0.64), rs3768617 (0.63), rs2296292 (0.63), rs10797819 (0.63), rs2296293 (0.63), rs8179460 (0.62), rs10911232 (0.62), rs10797843 (0.62), rs10752902 (0.62), rs4651141 (0.61), rs12082051 (0.61), rs10737245 (0.61), rs6666259 (0.61), rs6424879 (0.6), rs10911217 (0.6), rs10797825 (0.6), rs1547712 (0.6), rs4642853 (0.6), rs10911250 (0.59), rs10911199 (0.59), rs10911222 (0.59), rs10797828 (0.59), rs12403189 (0.58), rs10752885 (0.58), rs10911262 (0.58), rs36043504 (0.58), rs4652775 (0.57), rs28477876 (0.57), rs10797803 (0.57), rs2333620 (0.57), rs9943229 (0.56), rs10911209 (0.56), rs28533496 (0.56), rs10797807 (0.56), rs6701679 (0.55), rs10911189 (0.55), rs1547714 (0.55), rs10797832 (0.55), rs4651143 (0.55), rs12095664 (0.54), rs4369195 (0.54), rs1413386 (0.54), rs4651145 (0.54), rs10797837 (0.53), rs6692097 (0.53), rs3765521 (0.53), rs9787083 (0.53), rs3768619 (0.52), rs10911206 (0.52), rs12077201 (0.52), rs4596846 (0.52), rs12022720 (0.51), rs10752899 (0.51), rs2333621 (0.51), rs4072709 (0.51), rs4652771 (0.5), rs6697739 (0.5), rs6695837 (0.5), rs10797831 (0.5), rs10911191 (0.5), rs3768629 (0.49), rs61804970 (0.49), rs6697320 (0.49), rs10797841 (0.49), rs12037585 (0.48), rs4233194 (0.48), rs8179283 (0.48), rs1886499 (0.48), rs7518957 (0.47), rs4233192 (0.47), rs10911195 (0.47), rs10752882 (0.47), rs35968946 (0.46), rs6686554 (0.46), rs4652778 (0.46), rs4593781 (0.46), rs10911221 (0.45), rs2093985 (0.45), rs944970 (0.45), rs10737243 (0.45), rs4491025 (0.45), rs2027078 (0.44), rs6664995 (0.44), rs2027086 (0.44), rs10752894 (0.43), rs3934697 (0.43), rs20561 (0.43), rs7547953 (0.43), rs3736888 (0.42), rs3768622 (0.42), rs113153546 (0.42), rs2151669 (0.42), rs10737242 (0.41), rs10737236 (0.41), rs2296291 (0.41), rs3768625 (0.41), rs2027080 (0.4), rs2027085 (0.4), rs2027077 (0.4), rs10797811 (0.4), rs9425569 (0.4), rs11590958 (0.39), rs10797830 (0.39), rs2027082 (0.39), rs10797849 (0.39), rs4652776 (0.38), rs4442336 (0.38), rs10797834 (0.38),

		rs12120747 (0.38), rs4652763 (0.37), rs10752883 (0.37), rs10797840 (0.37), rs10911253 (0.37), rs10737238 (0.36), rs6657533 (0.36), rs10797817 (0.36), rs2147584 (0.36), rs6424880 (0.35), rs1537520 (0.35), rs7414273 (0.35), rs2333622 (0.35), rs10797824 (0.35), rs10911264 (0.34), rs2027076 (0.34), rs10911185 (0.34), rs10797809 (0.34), rs12133714 (0.33), rs1886501 (0.33), rs10752889 (0.33), rs9787327 (0.33), rs10752898 (0.32), rs9787228 (0.32), rs10911197 (0.32), rs4366285 (0.32), rs2027084 (0.31), rs7542277 (0.31), rs2296299 (0.31), rs20558 (0.31), rs10911245 (0.3), rs6695746 (0.3), rs20560 (0.3), rs7529465 (0.3), rs34099011 (0.29), rs12404594 (0.29), rs10911186 (0.29), rs4652769 (0.29), rs729672 (0.28), rs4652779 (0.28), rs3768616 (0.28), rs4546885 (0.28), rs10911229 (0.27), rs10797801 (0.27), rs4651140 (0.27), rs10797854 (0.27), rs4483371 (0.26), rs8179284 (0.26), rs12028740 (0.26), rs12043805 (0.26), rs3359 (0.25), rs9699754 (0.25), rs10732271 (0.25), rs2093984 (0.25), rs2147578 (0.25), rs6686682 (0.24), rs1062044 (0.24), rs10911230 (0.24), rs2333619 (0.24), rs1886500 (0.23), rs10752892 (0.23), rs10797833 (0.23), rs10752884 (0.23), rs10797851 (0.22), rs10911254 (0.22), rs4652757 (0.22), rs8179361 (0.22), rs4652766 (0.21), rs10737237 (0.21), rs4575047 (0.21), rs4652765 (0.2), rs10797808 (0.2), rs4420053 (0.2), rs10797853 (0.2), rs12739316 (0.2), rs6424882 (0.19), rs4409613 (0.19), rs10911259 (0.19), rs10797836 (0.19), rs12138053 (0.18), rs1051473 (0.18), rs12065110 (0.18), rs10911266 (0.18), rs200840404 (0.17), rs61804969 (0.17), rs10797822 (0.17), rs6673559 (0.17), rs20563 (0.16), rs10752904 (0.16), rs6692207 (0.16), rs3768623 (0.16), rs2296300 (0.15), rs12066735 (0.15), rs4652772 (0.15), rs4651139 (0.15), rs10737235 (0.15), rs10911196 (0.14), rs4396114 (0.14), rs9943069 (0.14), rs10752890 (0.14), rs6424883 (0.13), rs6672093 (0.13), rs3935384 (0.13), rs10737244 (0.13), rs10752893 (0.12), rs4651138 (0.12), rs2296297 (0.12), rs10797805 (0.12), rs4652758 (0.11), rs1413387 (0.11), rs7549768 (0.11), rs3820697 (0.11), rs10911239 (0.1), rs4129857 (0.1), rs10911251 (0.1), rs12026314 (0.1), rs10911248 (0.1), rs1889307 (0.09), rs4397624 (0.09), rs61808068 (0.09), rs2027081 (0.09), rs6660111 (0.08), rs10911243 (0.08), rs10752881 (0.08), rs12035773 (0.08), rs4047797 (0.07), rs3768621 (0.07), rs3768624 (0.07), rs10797835 (0.07), rs6424884 (0.06), rs723014 (0.06), rs10752897 (0.06), rs6679642 (0.06), rs1413389 (0.05), rs4651144 (0.05), rs12028732 (0.05), rs3768626 (0.05), rs4651137 (0.05), rs2027083 (0.04), rs4285667 (0.04), rs10752886 (0.04), rs4652767 (0.04), rs10911235 (0.03), rs3765522 (0.03), rs4304541 (0.03), rs10458355 (0.03), rs10911265 (0.02), rs35624846 (0.02), rs4402094 (0.02), rs10911194 (0.02), rs12138049 (0.01), rs4652780 (0.01), rs12037623 (0.01), rs10911211 (0.01), rs10797850 (0), rs10911212 (0)
rs28850399	12	rs721891 (1), rs74256604 (0.92), rs17761883 (0.85), rs28773614 (0.77), rs74578418 (0.69), rs56361256 (0.62), rs2191668 (0.54), rs28853598 (0.46), rs74256605 (0.38), rs2109657 (0.31), rs76748600 (0.23), rs10421380 (0.08)
rs3130070	79	rs3132445 (1), rs3130618 (0.99), rs9267543 (0.98), rs9267578 (0.96), rs3115674 (0.95), rs553414 (0.94), rs9267544 (0.93), rs3117574 (0.91), rs2763980 (0.9), rs3115671 (0.89), rs3130476 (0.88), rs3131381 (0.86), rs3130622 (0.85), rs3130048 (0.84), rs3130628 (0.83), rs3130490 (0.81), rs3115672 (0.8), rs9267535 (0.79), rs67682613 (0.78), rs3115663 (0.76), rs3117582 (0.75), rs11229 (0.74), rs3117573 (0.73), rs9267533 (0.71), rs3130478 (0.7), rs3117577 (0.69), rs3130624 (0.68), rs9267531 (0.66), rs3117580 (0.65), rs2607014 (0.63), rs558702 (0.61), rs3131383 (0.6),

		rs3115664 (0.59), rs3115665 (0.58), rs9267545 (0.56), rs3117579 (0.55), rs3132450 (0.54), rs3132451 (0.53), rs3117581 (0.51), rs3130679 (0.5), rs3131380 (0.49), rs515688 (0.48), rs9267540 (0.46), rs9267541 (0.45), rs3130484 (0.44), rs9267529 (0.43), rs3130626 (0.41), rs2753961 (0.4), rs625421 (0.39), rs9267549 (0.38), rs2142234 (0.36), rs9267539 (0.35), rs2736425 (0.34), rs3131379 (0.33), rs3130621 (0.31), rs3130623 (0.3), rs3130046 (0.29), rs3132443 (0.28), rs9267574 (0.26), rs3117575 (0.25), rs10885 (0.24), rs3132449 (0.23), rs3117576 (0.21), rs681331 (0.2), rs1265947 (0.19), rs693906 (0.18), rs3117583 (0.16), rs599707 (0.15), rs13295 (0.14), rs915651 (0.13), rs3130047 (0.11), rs3130627 (0.1), rs3101017 (0.09), rs3101018 (0.08), rs3131378 (0.06), rs2736157 (0.05), rs915652 (0.04), rs9267534 (0.03), rs10573 (0.01)
rs34166099	186	rs35748951 (1), rs66638185 (0.99), rs1486572 (0.99), rs1386173 (0.98), rs10446682 (0.98), rs11097757 (0.97), rs4698978 (0.97), rs11944613 (0.96), rs71597109 (0.96), rs28396651 (0.95), rs17200824 (0.95), rs13103438 (0.94), rs3733197 (0.94), rs13146194 (0.93), rs9790475 (0.93), rs4610336 (0.92), rs4698975 (0.91), rs13135381 (0.91), rs10013187 (0.9), rs4554079 (0.9), rs4463068 (0.89), rs11736568 (0.89), rs34814827 (0.88), rs138641783 (0.88), rs4365725 (0.87), rs13129744 (0.87), rs34499378 (0.86), rs11931087 (0.86), rs13101528 (0.85), rs11931658 (0.85), rs17266552 (0.84), rs2851333 (0.84), rs7686702 (0.83), rs67800004 (0.82), rs10446708 (0.82), rs10016018 (0.81), rs4496585 (0.81), rs13141934 (0.8), rs4530633 (0.8), rs1125271 (0.79), rs7682346 (0.79), rs35201947 (0.78), rs2850387 (0.78), rs4296682 (0.77), rs60448423 (0.77), rs58654655 (0.76), rs13126550 (0.76), rs2002758 (0.75), rs13119516 (0.74), rs4698974 (0.73), rs11935577 (0.73), rs4295265 (0.72), rs11933157 (0.72), rs4345184 (0.71), rs11097760 (0.71), rs13108400 (0.7), rs4493533 (0.7), rs7679882 (0.69), rs35388091 (0.69), rs11932972 (0.68), rs11940934 (0.68), rs1363067 (0.67), rs35979946 (0.66), rs4698839 (0.66), rs4398527 (0.65), rs12331595 (0.65), rs2851334 (0.64), rs13136796 (0.64), rs4699259 (0.63), rs4637409 (0.63), rs13117264 (0.62), rs57799233 (0.62), rs12331849 (0.61), rs2851329 (0.61), rs1027013 (0.6), rs2052445 (0.6), rs13136118 (0.59), rs10516487 (0.59), rs28582881 (0.58), rs35838403 (0.57), rs4639076 (0.57), rs17200433 (0.56), rs189908943 (0.56), rs13152198 (0.55), rs11939568 (0.55), rs11938185 (0.54), rs10446706 (0.54), rs2851336 (0.53), rs1352174 (0.53), rs1843680 (0.52), rs1386176 (0.52), rs4698977 (0.51), rs13107612 (0.51), rs2850383 (0.5), rs11929782 (0.49), rs7440537 (0.49), rs36128945 (0.48), rs6830565 (0.48), rs2631246 (0.47), rs13106926 (0.47), rs4698972 (0.46), rs2850377 (0.46), rs10446707 (0.45), rs6850747 (0.45), rs3974649 (0.44), rs4698971 (0.44), rs2850375 (0.43), rs11934097 (0.43), rs17031641 (0.42), rs2080820 (0.41), rs4270589 (0.41), rs35194352 (0.4), rs111541987 (0.4), rs4698979 (0.39), rs12498921 (0.39), rs11942437 (0.38), rs2851328 (0.38), rs13116542 (0.37), rs11944577 (0.37), rs4699262 (0.36), rs7441177 (0.36), rs6857428 (0.35), rs976654 (0.35), rs2631247 (0.34), rs2631248 (0.34), rs2850379 (0.33), rs4607219 (0.32), rs4235402 (0.31), rs11940179 (0.31), rs2851320 (0.3), rs7662572 (0.3), rs13128022 (0.29), rs34029191 (0.29), rs10856963 (0.28), rs10516483 (0.28), rs3974650 (0.27), rs34761248 (0.27), rs35446399 (0.26), rs13142517 (0.26), rs4572884 (0.25), rs80136231 (0.24), rs901406 (0.24), rs2631249 (0.23), rs34208976 (0.23), rs13107572 (0.22), rs923206 (0.22), rs12506564 (0.21), rs4699258 (0.21), rs13434472 (0.2), rs35860418 (0.2), rs2216546 (0.19), rs6816787 (0.19), rs34261083 (0.18), rs11940244 (0.18), rs2631244 (0.17), rs11938245 (0.16),

		rs376206821 (0.16), rs13131198 (0.15), rs2850378 (0.15), rs4699261 (0.14), rs35476121 (0.14), rs17266594 (0.13), rs1421627 (0.13), rs4443287 (0.12), rs4270588 (0.12), rs34749007 (0.11), rs55768089 (0.11), rs17266357 (0.1), rs13148076 (0.1), rs67330256 (0.09), rs75088535 (0.09), rs11734871 (0.08), rs4698969 (0.07), rs4699260 (0.07), rs11097761 (0.06), rs9999440 (0.06), rs34321727 (0.05), rs35416717 (0.05), rs143152246 (0.04), rs2851319 (0.04), rs11930376 (0.03), rs4426778 (0.03), rs13124747 (0.02), rs7656720 (0.02), rs114614648 (0.01), rs34851381 (0.01)
rs3809114	2	rs3809113 (1), rs703845 (0.67)
rs4412192	92	rs7771202 (1), rs7450556 (0.99), rs9379843 (0.98), rs6940237 (0.97), rs725575 (0.96), rs9467703 (0.95), rs989134 (0.94), rs12660432 (0.92), rs4145878 (0.91), rs806789 (0.9), rs6935954 (0.89), rs9357004 (0.88), rs9461242 (0.87), rs4412193 (0.86), rs9393697 (0.85), rs9357005 (0.84), rs9358916 (0.83), rs6939589 (0.82), rs1987244 (0.81), rs766406 (0.8), rs2179152 (0.78), rs7746807 (0.77), rs6908328 (0.76), rs806798 (0.75), rs766407 (0.74), rs2393593 (0.73), rs9379838 (0.72), rs7761298 (0.7), rs9393694 (0.69), rs62394764 (0.68), rs9358918 (0.67), rs9393691 (0.66), rs6916289 (0.65), rs9366651 (0.63), rs2179517 (0.62), rs6939048 (0.61), rs2142703 (0.6), rs4145910 (0.59), rs6930616 (0.58), rs17608582 (0.57), rs3999544 (0.56), rs7769028 (0.55), rs9366650 (0.54), rs1543681 (0.53), rs9393701 (0.52), rs806977 (0.51), rs9393690 (0.49), rs7748935 (0.48), rs9366648 (0.47), rs9366649 (0.46), rs809871 (0.45), rs34916901 (0.44), rs1125000 (0.43), rs7747133 (0.42), rs9358919 (0.41), rs6456712 (0.4), rs9358917 (0.39), rs6931391 (0.38), rs12661552 (0.37), rs9379844 (0.35), rs806790 (0.34), rs9393692 (0.33), rs9393696 (0.32), rs2893820 (0.31), rs9358927 (0.3), rs9358925 (0.29), rs61324092 (0.28), rs1059486 (0.27), rs11756673 (0.26), rs6918325 (0.25), rs5012374 (0.24), rs9393693 (0.23), rs9379846 (0.22), rs2893843 (0.2), rs75136657 (0.19), rs6456711 (0.18), rs2393592 (0.17), rs9358915 (0.16), rs4413606 (0.15), rs806795 (0.14), rs12525187 (0.13), rs9358920 (0.12), rs6456714 (0.11), rs6908402 (0.1), rs806785 (0.09), rs62394768 (0.08), rs9379842 (0.06), rs1124999 (0.05), rs28446029 (0.04), rs12526215 (0.03), rs1883213 (0.02), rs6899452 (0.01)
rs4464148	15	rs12967477 (1), rs12953717 (0.94), rs4939829 (0.88), rs17186877 (0.81), rs35715456 (0.75), rs2337107 (0.69), rs12967711 (0.63), rs2337106 (0.56), rs8085824 (0.5), rs34007497 (0.44), rs6507877 (0.31), rs58920878 (0.25), rs6507878 (0.19), rs9946510 (0.13), rs2878889 (0.06)
rs4675812	10	rs11684339 (1), rs2055568 (0.82), rs9679726 (0.73), rs13033734 (0.64), rs2012385 (0.55), rs6437249 (0.45), rs905661 (0.36), rs9288726 (0.27), rs905660 (0.18), rs871375 (0.09)
rs4899539	110	rs6574198 (1), rs11159114 (0.99), rs878206 (0.98), rs174997 (0.97), rs4899541 (0.96), rs4903266 (0.96), rs887746 (0.95), rs2230237 (0.94), rs2070599 (0.93), rs1860935 (0.92), rs11845260 (0.91), rs2080087 (0.9), rs6574203 (0.89), rs12891131 (0.88), rs2111705 (0.88), rs2853779 (0.87), rs11625594 (0.86), rs8016389 (0.85), rs6574200 (0.84), rs174999 (0.83), rs175000 (0.82), rs6574201 (0.81), rs55714011 (0.8), rs7141835 (0.79), rs10144023 (0.79), rs2286912 (0.78), rs4903264 (0.77), rs8010840 (0.76), rs7140660 (0.75), rs1319171 (0.74), rs10148293 (0.73), rs2009003 (0.72), rs933460 (0.71), rs8014289 (0.71), rs175001 (0.7), rs4903267 (0.69), rs12895452 (0.68), rs11159109 (0.67), rs12879725 (0.66), rs174995 (0.65),

		rs174998 (0.64), rs12889665 (0.63), rs3759741 (0.63), rs12894709 (0.62), rs2159904 (0.61), rs12588874 (0.6), rs8005138 (0.59), rs2070598 (0.58), rs10873274 (0.57), rs8006147 (0.56), rs7158047 (0.55), rs12880739 (0.54), rs2286913 (0.54), rs7156590 (0.53), rs10138360 (0.52), rs2005864 (0.51), rs2884668 (0.5), rs11159106 (0.49), rs2359237 (0.48), rs759058 (0.47), rs11621866 (0.46), rs28426374 (0.46), rs973879 (0.45), rs6574194 (0.44), rs2003490 (0.43), rs12147862 (0.42), rs11845259 (0.41), rs175002 (0.4), rs10143492 (0.39), rs957345 (0.38), rs8014374 (0.38), rs10083386 (0.37), rs10139018 (0.36), rs7149838 (0.35), rs2268615 (0.34), rs880949 (0.33), rs2159906 (0.32), rs4903270 (0.31), rs880948 (0.3), rs11159107 (0.29), rs2286914 (0.29), rs722685 (0.28), rs10139347 (0.27), rs4553560 (0.26), rs174994 (0.25), rs10483863 (0.24), rs741095 (0.23), rs6574197 (0.22), rs6574199 (0.21), rs7153740 (0.21), rs8014204 (0.2), rs11850328 (0.18), rs6574202 (0.17), rs7144654 (0.16), rs2268614 (0.15), rs11845397 (0.14), rs12432026 (0.13), rs2005863 (0.13), rs1799900 (0.12), rs4903262 (0.11), rs11625758 (0.1), rs7146041 (0.09), rs3742786 (0.08), rs3213716 (0.06), rs10141438 (0.05), rs4899540 (0.04), rs10162358 (0.04), rs2359240 (0.03), rs2300596 (0.02), rs11629408 (0.01)
rs4938340	245	rs144015791 (1), rs983567 (1), rs10892054 (0.99), rs1473326 (0.99), rs7946869 (0.98), rs11216187 (0.98), rs76523703 (0.98), rs61903418 (0.97), rs4938341 (0.97), rs80259223 (0.96), rs77320801 (0.96), rs7128071 (0.96), rs74328581 (0.95), rs2080586 (0.95), rs7107702 (0.94), rs115787357 (0.94), rs7130531 (0.94), rs61907604 (0.93), rs61905677 (0.93), rs73008568 (0.92), rs11216153 (0.92), rs7119185 (0.92), rs2513094 (0.91), rs11820814 (0.91), rs7101763 (0.9), rs61907591 (0.9), rs61905706 (0.9), rs10892055 (0.89), rs4936359 (0.89), rs7935913 (0.88), rs6589583 (0.88), rs7941428 (0.88), rs11216255 (0.87), rs113979111 (0.87), rs10892071 (0.86), rs721783 (0.86), rs75858236 (0.85), rs17120237 (0.85), rs113030965 (0.85), rs12721028 (0.84), rs61903395 (0.84), rs61905717 (0.83), rs61905687 (0.83), rs11600552 (0.83), rs6589587 (0.82), rs12274388 (0.82), rs4938338 (0.81), rs61905691 (0.81), rs7112111 (0.81), rs79530380 (0.8), rs7950213 (0.8), rs7946729 (0.79), rs4620758 (0.79), rs1473327 (0.79), rs11216239 (0.78), rs4938326 (0.78), rs11216162 (0.77), rs61903399 (0.77), rs7930783 (0.77), rs61903430 (0.76), rs111901638 (0.76), rs7930264 (0.75), rs61903419 (0.75), rs61905679 (0.75), rs1940626 (0.74), rs4388921 (0.74), rs55703909 (0.73), rs11823013 (0.73), rs10892045 (0.73), rs11827828 (0.72), rs112704540 (0.72), rs17120244 (0.71), rs78442878 (0.71), rs7930409 (0.71), rs59266239 (0.7), rs888245 (0.7), rs11216266 (0.69), rs3181 (0.69), rs4938321 (0.69), rs11216180 (0.68), rs76136280 (0.68), rs3886960 (0.67), rs59097294 (0.67), rs76690043 (0.67), rs17120233 (0.66), rs12279066 (0.66), rs1815788 (0.65), rs2070666 (0.65), rs1815789 (0.65), rs7124872 (0.64), rs61903398 (0.64), rs7950381 (0.63), rs11826742 (0.63), rs61905689 (0.63), rs59511712 (0.62), rs7128772 (0.62), rs78857232 (0.61), rs74341458 (0.61), rs61905712 (0.6), rs7123336 (0.6), rs4936363 (0.6), rs1145206 (0.59), rs12274465 (0.59), rs17120347 (0.58), rs4938342 (0.58), rs61903426 (0.58), rs17120280 (0.57), rs11823918 (0.57), rs4938336 (0.56), rs10790166 (0.56), rs112111936 (0.56), rs10892044 (0.55), rs12225230 (0.55), rs7106662 (0.54), rs7950093 (0.54), rs682048 (0.53), rs59700451 (0.53), rs12721030 (0.52), rs117287138 (0.52), rs888246 (0.52), rs75904569 (0.51), rs4316540 (0.51), rs11823889 (0.5), rs1531706 (0.5), rs17120111 (0.5), rs11216275 (0.49), rs1531707 (0.48), rs61905713 (0.48), rs61903429 (0.48), rs61903394 (0.47), rs12099358

		(0.47), rs45625638 (0.46), rs11216178 (0.46), rs7950501 (0.46), rs4938333 (0.45), rs113784559 (0.45), rs111327557 (0.44), rs61905705 (0.44), rs61905678 (0.44), rs4936362 (0.43), rs7946399 (0.43), rs723953 (0.42), rs61907594 (0.42), rs1871756 (0.42), rs7924318 (0.41), rs61905155 (0.41), rs56314469 (0.4), rs688456 (0.4), rs7941927 (0.4), rs79974099 (0.39), rs11216244 (0.39), rs4938327 (0.38), rs56081989 (0.38), rs76868258 (0.38), rs56016514 (0.37), rs17120251 (0.37), rs68137714 (0.36), rs7109876 (0.35), rs11216240 (0.35), rs2075547 (0.35), rs10892066 (0.34), rs77953246 (0.34), rs4936360 (0.33), rs7125788 (0.33), rs61905681 (0.33), rs61907605 (0.32), rs7104023 (0.32), rs7934763 (0.31), rs56227539 (0.31), rs113560866 (0.31), rs7112191 (0.3), rs4936361 (0.3), rs4938330 (0.29), rs11216241 (0.29), rs7108912 (0.29), rs6589580 (0.28), rs7939934 (0.28), rs112120205 (0.27), rs57582454 (0.27), rs1241655 (0.27), rs61903416 (0.26), rs112895963 (0.26), rs4938355 (0.25), rs11216169 (0.25), rs1473325 (0.25), rs61905718 (0.24), rs4938325 (0.24), rs1473324 (0.23), rs61903421 (0.23), rs7109649 (0.23), rs7484045 (0.22), rs4938329 (0.22), rs7935834 (0.21), rs4938357 (0.21), rs61905716 (0.21), rs12271161 (0.2), rs111560312 (0.2), rs76904522 (0.19), rs11216245 (0.19), rs11216168 (0.19), rs56371319 (0.18), rs17120241 (0.18), rs7124996 (0.17), rs7112967 (0.17), rs56134734 (0.17), rs625145 (0.16), rs7118591 (0.16), rs7932655 (0.15), rs2513097 (0.15), rs4938339 (0.15), rs10892075 (0.14), rs74691084 (0.14), rs74428560 (0.13), rs112151056 (0.13), rs4938317 (0.13), rs10082609 (0.12), rs17120197 (0.12), rs61905145 (0.11), rs61903417 (0.11), rs61903423 (0.1), rs1473177 (0.1), rs75920871 (0.1), rs76972960 (0.09), rs113184611 (0.09), rs61903427 (0.08), rs61905709 (0.08), rs61624438 (0.08), rs1838757 (0.07), rs10892042 (0.07), rs77250716 (0.06), rs509171 (0.06), rs723955 (0.06), rs4548653 (0.05), rs7946219 (0.05), rs11826651 (0.04), rs184090774 (0.04), rs61905680 (0.04), rs7931398 (0.03), rs10466590 (0.03), rs61905719 (0.02), rs7130213 (0.02), rs6589595 (0.02), rs67201490 (0.01), rs59294170 (0.01), rs670 (0)
rs56105022	62	rs147479860 (1), rs143868723 (0.98), rs4463628 (0.97), rs12120308 (0.95), rs10916472 (0.94), rs72751628 (0.92), rs544428583 (0.9), rs72754000 (0.89), rs72754001 (0.87), rs12126765 (0.86), rs12140137 (0.84), rs10916481 (0.83), rs72753994 (0.81), rs12128376 (0.79), rs75036533 (0.78), rs76962725 (0.76), rs12184283 (0.75), rs60256604 (0.73), rs150817716 (0.71), rs116590674 (0.7), rs12125241 (0.68), rs10799491 (0.67), rs12082619 (0.65), rs76955951 (0.63), rs10916458 (0.62), rs72751651 (0.6), rs12133599 (0.59), rs7552239 (0.57), rs7514140 (0.56), rs72753969 (0.54), rs10916497 (0.52), rs12124547 (0.51), rs141470848 (0.49), rs45583539 (0.48), rs12083770 (0.46), rs112600662 (0.44), rs12138866 (0.43), rs115310585 (0.41), rs12117152 (0.4), rs10916414 (0.38), rs10799507 (0.37), rs10916498 (0.35), rs72753956 (0.32), rs6660194 (0.3), rs151143241 (0.29), rs6686449 (0.27), rs150967467 (0.25), rs139734197 (0.24), rs573877473 (0.22), rs10916462 (0.21), rs10799543 (0.19), rs12144137 (0.17), rs6694451 (0.16), rs116232045 (0.14), rs72753980 (0.13), rs12129540 (0.11), rs72753910 (0.1), rs74750827 (0.08), rs6701036 (0.06), rs72746911 (0.05), rs12126815 (0.03), rs12117480 (0.02)
rs56343119	27	rs17474001 (1), rs4962153 (0.96), rs28602591 (0.93), rs2519093 (0.89), rs3124764 (0.86), rs28446901 (0.82), rs507666 (0.79), rs41302667 (0.75), rs28645493 (0.71), rs3758348 (0.68), rs3094379 (0.64), rs11244061 (0.61), rs3124766 (0.57), rs76238963 (0.54), rs587600964 (0.5), rs9650778

		(0.46), rs3124761 (0.43), rs739468 (0.39), rs532436 (0.36), rs41296107 (0.29), rs635634 (0.25), rs115478735 (0.21), rs36222279 (0.18), rs28602660 (0.14), rs41302673 (0.11), rs28658542 (0.07), rs28538967 (0.04)
rs6059958	317	rs6060007 (1), rs6060101 (1), rs6120905 (0.99), rs6058189 (0.99), rs6058470 (0.99), rs8120526 (0.98), rs6088669 (0.98), rs74392247 (0.98), rs3203770 (0.98), rs6060195 (0.97), rs6060960 (0.97), rs6579238 (0.97), rs6060296 (0.96), rs6060252 (0.96), rs7271374 (0.96), rs6059983 (0.95), rs6119720 (0.95), rs8126422 (0.95), rs6120898 (0.94), rs6059984 (0.94), rs6087710 (0.94), rs6060142 (0.93), rs3181073 (0.93), rs6089094 (0.93), rs6060261 (0.93), rs6119449 (0.92), rs1994251 (0.92), rs7273756 (0.92), rs6058151 (0.91), rs6060411 (0.91), rs6060399 (0.91), rs58875461 (0.9), rs58662941 (0.9), rs8121699 (0.9), rs57574578 (0.89), rs6060359 (0.89), rs78881751 (0.89), rs6119539 (0.88), rs2376992 (0.88), rs6058033 (0.88), rs6121221 (0.88), rs6060086 (0.87), rs6057911 (0.87), rs6060633 (0.87), rs6120937 (0.86), rs6087778 (0.86), rs6059982 (0.86), rs2889528 (0.85), rs7271671 (0.85), rs6088446 (0.85), rs6119729 (0.84), rs8123569 (0.84), rs6087716 (0.84), rs6059713 (0.83), rs6120832 (0.83), rs6060198 (0.83), rs6088793 (0.83), rs139441694 (0.82), rs6058069 (0.82), rs6060174 (0.82), rs6060114 (0.81), rs6059929 (0.81), rs6120671 (0.8), rs6087701 (0.8), rs7269866 (0.8), rs2376987 (0.79), rs7265413 (0.79), rs2016742 (0.79), rs6060289 (0.79), rs7272062 (0.78), rs6058086 (0.78), rs6060291 (0.77), rs6058152 (0.77), rs79072922 (0.77), rs6060116 (0.76), rs6058088 (0.76), rs6088736 (0.76), rs6059899 (0.75), rs6059690 (0.75), rs6088858 (0.75), rs2889488 (0.74), rs1075698 (0.74), rs2376862 (0.74), rs7261354 (0.74), rs6088451 (0.73), rs6057912 (0.73), rs2233834 (0.73), rs6058203 (0.72), rs115585940 (0.72), rs6060929 (0.72), rs6120862 (0.71), rs6088592 (0.71), rs6058058 (0.71), rs750144 (0.7), rs6058103 (0.7), rs6120815 (0.7), rs6060978 (0.69), rs138322105 (0.69), rs6059948 (0.69), rs6058140 (0.69), rs6058448 (0.68), rs6060821 (0.68), rs2376864 (0.68), rs6060793 (0.67), rs6060979 (0.67), rs6060145 (0.67), rs6060870 (0.66), rs6060987 (0.66), rs6119725 (0.66), rs6058460 (0.65), rs6060249 (0.65), rs6060812 (0.65), rs6120704 (0.64), rs6059795 (0.64), rs6120618 (0.64), rs6060381 (0.64), rs6119610 (0.63), rs6120868 (0.63), rs6120609 (0.63), rs6088814 (0.62), rs113818546 (0.62), rs6058102 (0.62), rs6059806 (0.61), rs6058171 (0.61), rs6060262 (0.61), rs6060210 (0.6), rs6059906 (0.6), rs6060263 (0.6), rs6058087 (0.6), rs6058196 (0.59), rs6087687 (0.59), rs6088806 (0.59), rs6088438 (0.58), rs6121209 (0.58), rs6121016 (0.58), rs112367115 (0.57), rs6060087 (0.57), rs6057968 (0.57), rs6088790 (0.56), rs6060118 (0.56), rs6060403 (0.56), rs6059874 (0.55), rs1471782 (0.55), rs6059952 (0.55), rs7346070 (0.55), rs6058023 (0.54), rs7270584 (0.54), rs6088374 (0.54), rs6059980 (0.53), rs6060089 (0.53), rs6059981 (0.53), rs6058450 (0.52), rs6059930 (0.52), rs6088864 (0.52), rs6060446 (0.51), rs6060425 (0.51), rs6088434 (0.51), rs6089071 (0.5), rs112805309 (0.5), rs6088962 (0.5), rs6058197 (0.5), rs6059702 (0.49), rs113400846 (0.49), rs80080420 (0.49), rs17093657 (0.48), rs6058110 (0.48), rs2889487 (0.48), rs6060171 (0.47), rs73235789 (0.47), rs15817 (0.47), rs6088462 (0.46), rs6060763 (0.46), rs1115713 (0.46), rs61676755 (0.45), rs6058035 (0.45), rs6119574 (0.45), rs1994250 (0.45), rs6060937 (0.44), rs186816175 (0.44), rs6088854 (0.44), rs56153385 (0.43), rs6058004 (0.43), rs6120700 (0.43), rs8126355 (0.42), rs6120719 (0.42), rs7263175 (0.42), rs6058122 (0.41), rs112200607 (0.41), rs6089070 (0.41), rs6060679 (0.4), rs7354225 (0.4), rs1923026 (0.4),

		rs6060247 (0.4), rs6088855 (0.39), rs3932482 (0.39), rs6060144 (0.39), rs7361893 (0.38), rs6119651 (0.38), rs752841 (0.38), rs6060474 (0.37), rs58757492 (0.37), rs56090668 (0.37), rs45557936 (0.36), rs75950492 (0.36), rs6057998 (0.36), rs6088796 (0.36), rs113103684 (0.35), rs6119489 (0.35), rs6058116 (0.35), rs6058172 (0.34), rs6120835 (0.34), rs6058131 (0.34), rs6060134 (0.33), rs2376996 (0.33), rs6088798 (0.33), rs6120731 (0.32), rs6119718 (0.32), rs6089055 (0.32), rs6060323 (0.31), rs6060260 (0.31), rs113502509 (0.31), rs6120607 (0.31), rs6058463 (0.3), rs6059830 (0.3), rs6120619 (0.3), rs6089058 (0.29), rs2038397 (0.29), rs6060002 (0.29), rs6089050 (0.28), rs6120713 (0.28), rs2376990 (0.28), rs879597 (0.27), rs6060989 (0.27), rs6120879 (0.27), rs113933395 (0.26), rs6059727 (0.26), rs2064818 (0.26), rs368329232 (0.26), rs6059696 (0.25), rs73235714 (0.25), rs57726761 (0.25), rs6120451 (0.24), rs6089093 (0.24), rs142923745 (0.24), rs11905777 (0.23), rs7274269 (0.23), rs6088749 (0.23), rs6059979 (0.22), rs7362275 (0.22), rs6119457 (0.22), rs6060066 (0.21), rs6059869 (0.21), rs6060923 (0.21), rs6060409 (0.21), rs6579239 (0.2), rs6059634 (0.2), rs6060076 (0.2), rs6060203 (0.19), rs6059846 (0.19), rs6058187 (0.19), rs1994249 (0.18), rs6119472 (0.18), rs6088853 (0.18), rs6060297 (0.17), rs6058421 (0.17), rs6120858 (0.17), rs6119575 (0.17), rs6119442 (0.16), rs6120452 (0.16), rs6120970 (0.16), rs6121242 (0.15), rs6088857 (0.15), rs6060286 (0.15), rs6058022 (0.14), rs717064 (0.14), rs6087700 (0.14), rs6060644 (0.13), rs6060344 (0.13), rs6060832 (0.13), rs73241710 (0.12), rs2376863 (0.12), rs6088856 (0.12), rs6059796 (0.12), rs6059889 (0.11), rs6060992 (0.11), rs6059888 (0.11), rs2839862 (0.1), rs6120890 (0.09), rs6088998 (0.09), rs6058233 (0.09), rs6060075 (0.08), rs79392518 (0.08), rs6059870 (0.08), rs11906851 (0.07), rs6060395 (0.07), rs6059781 (0.07), rs6120950 (0.07), rs6060295 (0.06), rs1406920 (0.06), rs6058141 (0.06), rs4607 (0.05), rs6059761 (0.05), rs7266471 (0.05), rs6059704 (0.04), rs6058148 (0.04), rs6059894 (0.04), rs6059841 (0.03), rs113882388 (0.03), rs6087711 (0.03), rs6060211 (0.02), rs6058085 (0.02), rs6059873 (0.02), rs6060912 (0.02), rs6120670 (0.01), rs2811 (0.01), rs144791144 (0.01), rs4518038 (0)
rs6420185	30	rs56960668 (1), rs34039115 (0.97), rs11136271 (0.94), rs9650460 (0.9), rs7388248 (0.87), rs7822123 (0.84), rs35664331 (0.77), rs6989167 (0.74), rs10087900 (0.71), rs66854753 (0.68), rs10099293 (0.65), rs11538388 (0.61), rs11136269 (0.58), rs12676568 (0.55), rs4076111 (0.52), rs34407803 (0.48), rs7460860 (0.45), rs72614045 (0.42), rs11136272 (0.39), rs7825338 (0.35), rs67149914 (0.32), rs10216471 (0.29), rs61059595 (0.26), rs62523434 (0.23), rs35297056 (0.19), rs13261822 (0.16), rs4074902 (0.13), rs9650459 (0.1), rs62523440 (0.06), rs4075205 (0.03)
rs67385163	50	rs11064372 (1), rs3168600 (0.98), rs7487291 (0.96), rs3815408 (0.94), rs7137525 (0.92), rs10774444 (0.9), rs34470255 (0.88), rs2302368 (0.86), rs4764512 (0.84), rs12578573 (0.82), rs1882547 (0.8), rs67706382 (0.78), rs35247189 (0.76), rs11064332 (0.75), rs35039612 (0.73), rs11064331 (0.71), rs6489731 (0.69), rs6489732 (0.67), rs11064370 (0.65), rs2302370 (0.63), rs2365567 (0.61), rs1882549 (0.59), rs7134028 (0.57), rs7307912 (0.55), rs10849500 (0.53), rs2365089 (0.51), rs2365092 (0.49), rs4764513 (0.47), rs871365 (0.45), rs11064369 (0.43), rs2286731 (0.41), rs1997510 (0.39), rs7296292 (0.37), rs7299012 (0.35), rs4492872 (0.33), rs17887160 (0.31), rs2178692 (0.27), rs2302366 (0.25), rs11227 (0.24), rs2178691 (0.22), rs11064373 (0.2), rs59226301 (0.18), rs7315926 (0.16), rs2365100 (0.14), rs11064371 (0.12), rs1882551 (0.1), rs7954666 (0.08), rs6489734

		(0.06), rs2302367 (0.04), rs7302581 (0.02)
rs686030	24	rs677578 (1), rs675849 (0.96), rs1215112 (0.92), rs2185938 (0.88), rs578194 (0.84), rs570524 (0.76), rs660686 (0.72), rs2156110 (0.68), rs540885 (0.64), rs4237138 (0.6), rs3905247 (0.56), rs535772 (0.52), rs471364 (0.48), rs3933784 (0.44), rs677622 (0.4), rs1475229 (0.36), rs3933785 (0.32), rs585002 (0.28), rs486349 (0.24), rs2156109 (0.2), rs661048 (0.16), rs581080 (0.12), rs2616751 (0.08), rs643531 (0.04)
rs7237346	16	rs75833060 (0.94), rs3829632 (0.88), rs7230119 (0.82), rs4245232 (0.76), rs6507929 (0.71), rs4939875 (0.65), rs10083905 (0.59), rs1943678 (0.53), rs8099312 (0.47), rs4556888 (0.41), rs4939583 (0.35), rs56341437 (0.29), rs2000812 (0.24), rs3819166 (0.18), rs7235707 (0.12), rs9958947 (0.06)
rs72665235	10	rs72665233 (1), rs142358687 (0.91), rs142483715 (0.82), rs372151476 (0.73), rs145830471 (0.64), rs200247105 (0.55), rs477913 (0.36), rs61779763 (0.27), rs115874855 (0.18), rs201407410 (0.09)
rs7268701	307	rs17092066 (1), rs4911433 (1), rs6059878 (0.99), rs6088512 (0.99), rs6088543 (0.99), rs6142210 (0.98), rs62212172 (0.98), rs2378135 (0.98), rs6142209 (0.97), rs6059882 (0.97), rs6059824 (0.97), rs1205341 (0.96), rs6141465 (0.96), rs6060015 (0.96), rs6087606 (0.95), rs1205335 (0.95), rs6088595 (0.95), rs2889850 (0.95), rs4911418 (0.94), rs6088466 (0.94), rs6088542 (0.94), rs55793516 (0.93), rs6088485 (0.93), rs1205346 (0.93), rs6059850 (0.92), rs6142154 (0.92), rs6088568 (0.92), rs6087605 (0.91), rs6088528 (0.91), rs4911158 (0.91), rs6059743 (0.9), rs6120668 (0.9), rs2064705 (0.9), rs6058051 (0.89), rs1205340 (0.89), rs4911423 (0.89), rs73610830 (0.88), rs6087600 (0.88), rs1890000 (0.88), rs6141509 (0.87), rs6088536 (0.87), rs11167241 (0.87), rs6120675 (0.86), rs4911445 (0.86), rs910870 (0.86), rs6088524 (0.85), rs6088483 (0.85), rs4911420 (0.85), rs6059851 (0.84), rs6088575 (0.84), rs6059859 (0.84), rs4911437 (0.83), rs6141503 (0.83), rs6142216 (0.83), rs6059867 (0.82), rs6059839 (0.82), rs2424997 (0.82), rs6088577 (0.81), rs13043504 (0.81), rs6059987 (0.81), rs2184836 (0.8), rs6059934 (0.8), rs6059834 (0.8), rs6059893 (0.79), rs4277599 (0.79), rs6141464 (0.79), rs8125522 (0.78), rs6120669 (0.78), rs1853056 (0.78), rs945673 (0.77), rs6059892 (0.77), rs6059866 (0.77), rs1205342 (0.77), rs6120644 (0.76), rs6088478 (0.76), rs6059856 (0.76), rs6088554 (0.75), rs6059956 (0.75), rs1853057 (0.75), rs6142137 (0.74), rs1018503 (0.74), rs11904852 (0.74), rs1205343 (0.73), rs4911151 (0.73), rs13043505 (0.73), rs6060048 (0.72), rs819140 (0.72), rs910869 (0.72), rs6088553 (0.71), rs6142201 (0.71), rs6059918 (0.71), rs2378256 (0.7), rs6088537 (0.7), rs9789792 (0.7), rs6142222 (0.69), rs6087614 (0.69), rs6142159 (0.69), rs1205345 (0.68), rs62213657 (0.68), rs2225837 (0.68), rs4911444 (0.68), rs6142147 (0.67), rs6088521 (0.67), rs721613 (0.67), rs72497006 (0.66), rs6087579 (0.66), rs6141482 (0.65), rs6059774 (0.65), rs6088488 (0.65), rs4911422 (0.64), rs6058049 (0.64), rs6088548 (0.64), rs533199207 (0.63), rs1321306 (0.63), rs6088479 (0.63), rs4911434 (0.62), rs6059840 (0.62), rs764597 (0.62), rs6142213 (0.61), rs6058067 (0.61), rs4911152 (0.61), rs6059896 (0.6), rs2424992 (0.6), rs6141514 (0.6), rs6087587 (0.59), rs6088565 (0.59), rs6058064 (0.59), rs2378133 (0.59), rs6060039 (0.58), rs1205338 (0.58), rs6141471 (0.58), rs6087592 (0.57), rs6120650 (0.57), rs2378205 (0.57), rs4142007 (0.56), rs6087609 (0.56), rs6058050 (0.56), rs6142212 (0.55), rs6060057 (0.55), rs6059897 (0.55), rs6059855 (0.54), rs6088569 (0.54), rs6060052 (0.54), rs6088502 (0.53),

		rs6087612 (0.53), rs6088547 (0.53), rs6058057 (0.52), rs1205344 (0.52), rs6088529 (0.52), rs6058029 (0.51), rs7271144 (0.51), rs11167234 (0.51), rs4911155 (0.5), rs7264012 (0.5), rs6141486 (0.5), rs6141493 (0.5), rs2425000 (0.49), rs6059860 (0.49), rs3761147 (0.49), rs6088559 (0.48), rs6059877 (0.48), rs3746455 (0.48), rs6059880 (0.47), rs6142202 (0.47), rs2378250 (0.47), rs6087580 (0.46), rs146006879 (0.46), rs2889855 (0.46), rs6088578 (0.45), rs7354735 (0.45), rs6088489 (0.45), rs13041646 (0.44), rs56049597 (0.44), rs6120620 (0.44), rs6142205 (0.43), rs4911153 (0.43), rs819139 (0.43), rs6059863 (0.42), rs6120642 (0.42), rs6059779 (0.42), rs1040747 (0.41), rs8122820 (0.41), rs6088561 (0.41), rs6579178 (0.41), rs6088471 (0.4), rs6141469 (0.4), rs6059887 (0.4), rs6088498 (0.39), rs2378254 (0.39), rs1986110 (0.39), rs6059843 (0.38), rs8114616 (0.38), rs6059797 (0.38), rs6058059 (0.37), rs6142208 (0.37), rs6088552 (0.37), rs11167245 (0.36), rs4911421 (0.36), rs12624640 (0.36), rs6087598 (0.35), rs7271970 (0.35), rs11700221 (0.35), rs6058052 (0.34), rs6059845 (0.34), rs6058106 (0.34), rs4911441 (0.33), rs6058084 (0.33), rs6059932 (0.33), rs562493105 (0.32), rs6579165 (0.32), rs67439923 (0.32), rs6579161 (0.32), rs6142152 (0.31), rs6058065 (0.31), rs6088484 (0.31), rs62213590 (0.3), rs13042351 (0.3), rs8183502 (0.3), rs4911429 (0.29), rs3787219 (0.29), rs8121066 (0.29), rs6059835 (0.28), rs6142164 (0.28), rs6141488 (0.28), rs6119497 (0.27), rs6142129 (0.27), rs6058041 (0.27), rs6087624 (0.26), rs1884669 (0.26), rs6087574 (0.26), rs6059844 (0.25), rs7274854 (0.25), rs6060013 (0.25), rs2378259 (0.24), rs6120663 (0.24), rs6088570 (0.24), rs6058073 (0.23), rs4911419 (0.23), rs6058108 (0.23), rs12480410 (0.23), rs2295444 (0.22), rs2180485 (0.22), rs1015363 (0.22), rs6087619 (0.21), rs6088560 (0.21), rs6087567 (0.21), rs6120666 (0.2), rs1205347 (0.2), rs6088527 (0.2), rs2144717 (0.19), rs6059890 (0.19), rs6119510 (0.19), rs6142157 (0.18), rs6141504 (0.18), rs6058070 (0.18), rs6088461 (0.17), rs6088475 (0.17), rs6059875 (0.17), rs1001425 (0.16), rs2378203 (0.16), rs6059829 (0.15), rs7269596 (0.15), rs2378251 (0.15), rs6059861 (0.14), rs6088523 (0.14), rs6059926 (0.14), rs6058040 (0.14), rs2424993 (0.13), rs1890002 (0.13), rs6088525 (0.13), rs6142219 (0.12), rs13044413 (0.12), rs4431018 (0.12), rs945674 (0.11), rs819135 (0.11), rs1205334 (0.11), rs6087616 (0.1), rs6059872 (0.1), rs6087615 (0.09), rs6060012 (0.09), rs2378204 (0.09), rs6087618 (0.08), rs6120645 (0.08), rs6059868 (0.08), rs6142206 (0.07), rs6059938 (0.07), rs4258875 (0.07), rs6060038 (0.06), rs6087588 (0.06), rs3891369 (0.06), rs2378134 (0.05), rs6142153 (0.05), rs6059827 (0.05), rs3928101 (0.05), rs6059858 (0.04), rs6088567 (0.04), rs57654090 (0.04), rs1205337 (0.03), rs3736762 (0.03), rs2105109 (0.03), rs6087577 (0.02), rs6088508 (0.02), rs6088532 (0.02), rs6088564 (0.01), rs6059905 (0.01), rs4911417 (0.01), rs1205336 (0)
rs72771478	1	rs72771479 (1)
rs73090625	48	rs73090632 (1), rs2322141 (0.98), rs1447906 (0.96), rs35897230 (0.94), rs61507336 (0.92), rs73090637 (0.9), rs13083133 (0.88), rs73092536 (0.86), rs73088638 (0.84), rs68021782 (0.82), rs36090610 (0.8), rs34550804 (0.78), rs4298016 (0.76), rs4387935 (0.73), rs35269191 (0.71), rs13099444 (0.69), rs73088604 (0.67), rs13098885 (0.65), rs184416061 (0.63), rs112400052 (0.61), rs2029012 (0.59), rs116066038 (0.57), rs75972158 (0.55), rs13064137 (0.53), rs13077647 (0.51), rs71298399 (0.49), rs35899311 (0.47), rs60690351 (0.45), rs548598109 (0.43), rs73090620 (0.41),

		rs116310555 (0.39), rs12106767 (0.37), rs73090626 (0.35), rs17008972 (0.33), rs74446449 (0.31), rs12631094 (0.29), rs4364137 (0.27), rs12630449 (0.22), rs34295566 (0.2), rs112669807 (0.18), rs11128221 (0.16), rs6772104 (0.14), rs13070445 (0.12), rs17664495 (0.1), rs4995840 (0.08), rs73092540 (0.06), rs13071396 (0.04), rs73090622 (0.02)
rs7412	6	rs390082 (1), rs190712692 (0.86), rs445925 (0.71), rs141622900 (0.57), rs72654473 (0.43), rs1065853 (0.14)
rs7595075	70	rs36216559 (1), rs7566279 (0.99), rs60484953 (0.97), rs57542652 (0.96), rs55932737 (0.94), rs7561648 (0.93), rs56174623 (0.92), rs7608141 (0.9), rs4497901 (0.89), rs7588043 (0.87), rs17713568 (0.86), rs62114494 (0.85), rs4430979 (0.83), rs55946380 (0.82), rs17041320 (0.8), rs55936726 (0.79), rs62116681 (0.77), rs300750 (0.76), rs17713396 (0.75), rs1078763 (0.73), rs11674477 (0.72), rs7601944 (0.7), rs17713879 (0.69), rs394791 (0.68), rs62114544 (0.66), rs62114501 (0.65), rs3791220 (0.63), rs11553746 (0.62), rs6710091 (0.61), rs60149603 (0.59), rs58461606 (0.58), rs17714252 (0.56), rs11674325 (0.55), rs114976176 (0.54), rs62114506 (0.52), rs6755432 (0.51), rs7584915 (0.49), rs75141812 (0.48), rs55987018 (0.46), rs62114538 (0.45), rs55753056 (0.44), rs56167434 (0.42), rs59937473 (0.41), rs3791223 (0.39), rs3791224 (0.38), rs300751 (0.37), rs17713729 (0.35), rs60843830 (0.34), rs2306060 (0.32), rs58416198 (0.31), rs79716074 (0.3), rs62114497 (0.28), rs2126129 (0.27), rs9213 (0.25), rs7605824 (0.24), rs549751842 (0.23), rs7608143 (0.21), rs62114505 (0.2), rs2290911 (0.18), rs79154857 (0.17), rs62116682 (0.15), rs62114548 (0.13), rs3791221 (0.11), rs56285383 (0.1), rs6721594 (0.08), rs6548219 (0.07), rs11695591 (0.06), rs55742348 (0.04), rs56321614 (0.03), rs3828165 (0.01)
rs77913157	0	
rs78608249	25	rs11656013 (1), rs9900496 (0.96), rs12936934 (0.92), rs9912001 (0.88), rs12938899 (0.85), rs858519 (0.81), rs9892297 (0.77), rs7502669 (0.73), rs727428 (0.69), rs9892862 (0.65), rs8073177 (0.62), rs12944649 (0.54), rs77294902 (0.5), rs2955617 (0.46), rs11651783 (0.42), rs9900162 (0.38), rs8081548 (0.35), rs9898876 (0.31), rs11078694 (0.27), rs858516 (0.23), rs888110 (0.19), rs9902027 (0.15), rs12602989 (0.12), rs6608 (0.08), rs17643401 (0.04)
rs9837992	102	rs35397914 (1), rs35234680 (0.99), rs934588 (0.98), rs9847154 (0.97), rs34577575 (0.96), rs6776440 (0.95), rs2687201 (0.94), rs35109540 (0.93), rs6549363 (0.92), rs17745575 (0.91), rs2293171 (0.9), rs35378923 (0.89), rs34936052 (0.88), rs1018340 (0.88), rs7611254 (0.87), rs13084963 (0.86), rs17802889 (0.85), rs2196356 (0.84), rs12054322 (0.83), rs934589 (0.82), rs11917200 (0.81), rs62257211 (0.8), rs56254492 (0.79), rs146460118 (0.78), rs35012514 (0.77), rs34371285 (0.76), rs13092463 (0.75), rs2699491 (0.74), rs1053797 (0.73), rs13094887 (0.72), rs56176196 (0.71), rs35270590 (0.7), rs2597305 (0.68), rs13092598 (0.67), rs35040844 (0.66), rs7635815 (0.65), rs12630880 (0.64), rs1403202 (0.63), rs6776545 (0.63), rs17802967 (0.62), rs34942426 (0.61), rs9310201 (0.59), rs1522551 (0.58), rs34341381 (0.57), rs2687199 (0.56), rs13097265 (0.55), rs2597312 (0.54), rs1533834 (0.53), rs17802799 (0.52), rs5007038 (0.51), rs1852922 (0.5), rs2687197 (0.49), rs1851428 (0.48), rs1522552 (0.47), rs13060374

(0.46), rs3773497 (0.45), rs13068175 (0.44), rs2597301 (0.43), rs6549366 (0.42), rs13074819 (0.41), rs9874089 (0.4), rs6790743 (0.39), rs2077610 (0.38), rs62257217 (0.38), rs62255645 (0.37), rs62257174 (0.36), rs6805430 (0.35), rs13093886 (0.34), rs13065564 (0.33), rs7626449 (0.32), rs4676893 (0.31), rs34440469 (0.3), rs17007949 (0.29), rs34603408 (0.28), rs6549362 (0.27), rs11928769 (0.26), rs6802023 (0.25), rs2687203 (0.24), rs17007912 (0.23), rs6801977 (0.22), rs13089093 (0.21), rs2687195 (0.2), rs6809836 (0.19), rs4499560 (0.18), rs7638679 (0.17), rs11919835 (0.16), rs34518676 (0.15), rs3846031 (0.14), rs138929390 (0.13), rs2140132 (0.13), rs2597302 (0.12), rs1522554 (0.11), rs11128199 (0.1), rs2687202 (0.09), rs11927771 (0.08), rs9845197 (0.07), rs11926363 (0.06), rs34552401 (0.05), rs62257173 (0.04), rs11128198 (0.03), rs12636552 (0.02), rs35236436 (0.01)

ALS: Amyotrophic lateral sclerosis; ApoA1: apolipoprotein A1; SNP: single nucleotide polymorphisms.
