# Supplementary material for: Shared genetic link and causal inference between blood lipids, lipid-lowering drugs and amyotrophic lateral sclerosis
Source: Neural Regen Res. 2025 Aug 13;21(8):3824–30. doi: 10.4103/NRR.NRR-D-25-00266 (PMC13452702; doi:10.4103/NRR.NRR-D-25-00266)
Supplement: Supplementary file 3 [file NRR-21-3824_Suppl3.pdf]

Additional Table 6 Co-localization analysis for loci shared between ALS and HDL-C or ApoA1

Traits	SNP	CHR	BP	PP.H0.abf	PP.H1.abf	PP.H2.abf	PP.H3.abf	Coloc H4 Prob
HDL-C	rs7726	1	26901319	1.31E-76	7.82E-01	3.00E-77	1.79E-01	3.96E-02
	rs72665235	1	40157643	1.68E-130	8.25E-01	3.44E-131	1.69E-01	6.14E-03
	rs650194	1	71414863	2.30E-08	5.63E-01	6.62E-09	1.62E-01	2.75E-01
	rs267738	1	150940625	1.61E-33	3.23E-01	4.90E-34	9.76E-02	5.80E-01
	rs56105022	1	224544746	2.18E-08	1.06E-01	1.35E-07	6.56E-01	2.38E-01
	rs7595075	2	264019	1.03E-21	7.66E-01	1.78E-22	1.33E-01	1.01E-01
	rs66906321	2	630070	9.76E-22	7.29E-01	2.33E-22	1.74E-01	9.66E-02
	rs1107850	2	20371772	3.47E-15	7.25E-01	6.43E-16	1.34E-01	1.41E-01
	rs6724214	2	54034881	1.10E-07	7.68E-01	3.26E-08	2.26E-01	5.99E-03
	rs2540948	2	65284623	2.07E-24	7.20E-01	6.76E-25	2.35E-01	4.55E-02
	rs2303564	2	219529881	8.49E-08	1.11E-01	1.54E-07	2.01E-01	6.88E-01
	rs1515114	2	227098387	1.38E-142	8.42E-01	2.16E-143	1.32E-01	2.65E-02
	rs4675812	2	242395674	3.03E-14	6.98E-01	7.16E-15	1.65E-01	1.38E-01
	rs111873781	3	49930316	2.09E-48	8.58E-01	3.36E-49	1.38E-01	4.84E-03
	rs4855447	3	69803328	3.89E-05	5.85E-01	1.12E-05	1.68E-01	2.47E-01
	**rs34311866**	4	951947	6.99E-17	2.56E-03	1.23E-15	4.42E-02	9.53E-01
	rs56122753	4	26031073	9.20E-24	8.20E-01	1.02E-24	9.09E-02	8.90E-02
	rs7684939	4	55509189	2.63E-07	6.17E-01	1.00E-07	2.34E-01	1.49E-01
	rs114614648	4	102810526	3.16E-188	8.22E-01	6.31E-189	1.64E-01	1.44E-02
	rs7736891	5	52808873	1.25E-15	6.86E-01	5.64E-16	3.10E-01	4.27E-03
	rs74557927	5	55825745	2.47E-57	8.28E-01	4.94E-58	1.66E-01	6.02E-03
	rs2112347	5	75015242	6.07E-23	7.56E-01	1.67E-23	2.07E-01	3.62E-02
	rs2434612	5	158022041	1.13E-29	7.25E-01	3.68E-30	2.36E-01	3.91E-02

rs62396278	6	31329119	4.91E-34	1.12E-02	4.33E-32	9.89E-01	2.56E-04
rs9268839	6	32428772	1.78E-25	2.73E-06	6.52E-20	1.00E+00	6.84E-08
rs3800406	6	35133074	2.38E-81	6.08E-01	1.50E-81	3.82E-01	1.04E-02
rs3798241	6	116292883	4.59E-21	7.52E-01	1.43E-21	2.34E-01	1.38E-02
rs2186037	6	127455029	1.73E-42	9.03E-01	1.61E-43	8.40E-02	1.30E-02
rs3808184	7	117214125	8.13E-10	8.38E-01	1.50E-10	1.55E-01	6.40E-03
rs17717355	8	9198695	0	0.648	0	0.342	0.00947
rs77705851	8	9293292	0	0.651	0	0.34	0.0095
rs77913157	8	19949198	0	0.774	0	0.219	0.0075
rs3808461	8	116597716	1.06E-113	4.93E-01	2.45E-114	1.14E-01	3.93E-01
rs6420185	8	144303071	4.59E-48	8.46E-01	7.21E-49	1.33E-01	2.11E-02
rs686030	9	15304782	1.57E-132	6.34E-01	3.71E-133	1.50E-01	2.16E-01
rs2492816	9	27565105	8.30E-37	2.94E-38	9.65E-01	3.42E-02	8.09E-04
rs117323391	9	107511975	0	0.76	0	0.229	0.0109
rs56343119	9	136237672	1.04E-15	8.40E-01	1.69E-16	1.37E-01	2.31E-02
rs7098444	10	75580042	2.51E-05	4.74E-01	1.21E-05	2.28E-01	2.98E-01
rs2792751	10	113940329	1.83E-83	5.48E-01	1.12E-83	3.36E-01	1.16E-01
rs11039389	11	47796062	1.64E-158	8.18E-01	3.40E-159	1.70E-01	1.18E-02
rs117186302	11	61456420	2.14E-213	8.84E-01	2.62E-214	1.09E-01	7.01E-03
rs12575636	11	95311260	2.56E-05	6.55E-01	5.50E-06	1.40E-01	2.05E-01
rs4938340	11	116980622	0	0.8	0	0.185	0.0143
rs11064373	12	6871709	4.82E-16	3.42E-01	9.15E-16	6.50E-01	8.65E-03
rs3809114	12	57848639	1.17E-63	1.93E-05	6.07E-59	1.00E+00	2.49E-07
rs10774625	12	111910219	3.56E-49	8.74E-01	4.11E-50	1.01E-01	2.49E-02
rs17532413	13	41644956	1.35E-14	7.18E-01	1.92E-15	1.02E-01	1.80E-01

	rs113629348	15	58545668	0	0.799	0	0.18	0.0213
	rs2058914	15	63831984	3.48E-25	7.39E-01	1.20E-25	2.55E-01	6.04E-03
	rs62036658	16	28850371	6.53E-07	4.51E-01	2.42E-07	1.67E-01	3.82E-01
	rs117254618	16	56892818	0	0.76	0	0.222	0.0177
	rs72771478	16	57002118	0	0.766	0	0.216	0.0178
	rs1109166	16	67977382	5.25E-239	4.23E-02	1.19E-237	9.57E-01	3.53E-04
	rs16952930	17	401847	4.96E-11	8.77E-01	6.02E-12	1.06E-01	1.62E-02
	**rs3803800**	17	7462969	4.05E-24	2.74E-02	8.81E-24	5.88E-02	9.14E-01
	rs10853128	17	26721304	4.93E-07	5.36E-02	8.51E-06	9.25E-01	2.09E-02
	rs12453334	17	38153473	9.27E-87	8.59E-01	1.46E-87	1.35E-01	5.50E-03
	rs7213935	17	41801263	0	0.832	0	0.152	0.0155
	rs8078686	17	45735706	1.84E-18	8.00E-01	3.48E-19	1.51E-01	4.85E-02
	rs28412876	17	47454515	8.20E-09	7.95E-01	1.18E-09	1.15E-01	8.99E-02
	**rs4969182**	17	76393030	3.39E-96	7.97E-02	7.63E-96	1.79E-01	7.42E-01
	rs3819166	18	47102102	0	0.769	0	0.217	0.0146
	rs7254731	19	3405692	1.55E-16	8.91E-01	1.79E-17	1.03E-01	6.01E-03
	rs35390907	19	8393592	2.15E-113	9.05E-01	2.01E-114	8.46E-02	1.00E-02
	rs73045226	19	41760913	9.86E-11	8.63E-01	1.18E-11	1.03E-01	3.35E-02
	rs7412	19	45412079	0	0.883	0	0.0951	0.0221
	rs28850399	19	52314227	9.41E-33	8.40E-01	1.72E-33	1.53E-01	6.85E-03
	rs6058035	20	30120782	7.71E-11	7.98E-01	1.30E-11	1.34E-01	6.73E-02
	rs7268701	20	32901960	1.38E-29	8.62E-01	1.96E-30	1.23E-01	1.52E-02
	rs9609261	22	31743032	2.48E-05	2.09E-01	8.75E-05	7.40E-01	5.07E-02
ApoA1	rs72665235	1	40157643	6.59E-59	8.80E-01	8.49E-60	1.13E-01	6.56E-03
	rs267738	1	150940625	2.50E-41	3.30E-01	3.93E-42	5.12E-02	6.19E-01

rs2877984	1	183090497	1.49E-05	6.48E-01	3.32E-06	1.44E-01	2.07E-01
rs56105022	1	224544746	4.87E-06	9.99E-02	2.96E-05	6.07E-01	2.93E-01
rs7595075	2	264019	1.20E-04	7.93E-01	1.61E-05	1.06E-01	1.01E-01
rs1107850	2	20371772	7.21E-26	7.62E-01	9.53E-27	1.01E-01	1.37E-01
rs1515114	2	227098387	3.90E-55	8.74E-01	4.44E-56	9.95E-02	2.65E-02
rs4675812	2	242395674	5.43E-10	8.50E-01	9.18E-11	1.44E-01	6.20E-03
rs9837992	3	70959438	0.00185	0.679	0.00046	0.168	0.151
rs73090625	3	71780696	1.40E-04	8.11E-01	2.07E-05	1.20E-01	6.96E-02
rs11248061	4	980896	1.43E-05	4.02E-02	2.51E-04	7.05E-01	2.54E-01
rs1022186	4	26037388	1.31E-10	8.17E-01	1.16E-11	7.23E-02	1.11E-01
rs11133338	4	55513275	0.000631	0.579	0.000193	0.177	0.244
rs34166099	4	102778580	3.40E-75	8.60E-01	4.95E-76	1.25E-01	1.51E-02
rs2434612	5	158022041	2.17E-08	7.33E-01	5.79E-09	1.96E-01	7.08E-02
rs2446192	5	172352369	0.000292	0.000928	0.235	0.747	0.0173
rs4412192	6	26290377	1.36E-11	4.71E-01	1.40E-11	4.85E-01	4.45E-02
rs3130070	6	31591808	7.94E-31	1.12E-02	7.02E-29	9.89E-01	2.16E-04
rs11755266	6	35162141	1.36E-25	6.10E-01	7.58E-26	3.39E-01	5.08E-02
rs2489623	6	127455821	3.00E-08	9.08E-01	2.44E-09	7.39E-02	1.78E-02
rs17717355	8	9198695	1.50E-131	6.84E-01	6.72E-132	3.06E-01	9.95E-03
rs17153747	8	11611353	2.19E-11	8.71E-01	3.08E-12	1.22E-01	6.25E-03
rs77913157	8	19949198	0	0.86	0	0.132	0.00834
rs10505258	8	116602855	8.06E-64	5.49E-01	1.47E-64	9.94E-02	3.52E-01
rs6420185	8	144303071	9.00E-40	8.63E-01	1.17E-40	1.13E-01	2.47E-02
rs686030	9	15304782	7.54E-82	6.61E-01	1.31E-82	1.14E-01	2.25E-01
rs117323391	9	107511975	1.43E-278	8.61E-01	2.09E-279	1.26E-01	1.23E-02

rs56343119	9	136237672	7.23E-27	8.63E-01	9.48E-28	1.13E-01	2.40E-02
rs2792751	10	113940329	4.64E-59	5.88E-01	2.34E-59	2.97E-01	1.16E-01
rs11039324	11	47665686	1.15E-56	8.89E-01	1.29E-57	1.00E-01	1.09E-02
rs11021232	11	95320808	4.03E-04	7.21E-01	4.98E-05	8.90E-02	1.89E-01
rs4938340	11	116980622	9.881313e-324	8.75E-01	0.00E+00	1.11E-01	1.41E-02
rs67385163	12	6864890	3.54E-05	3.12E-01	6.51E-05	5.76E-01	1.12E-01
rs3809114	12	57848639	6.35E-32	1.93E-05	3.28E-27	1.00E+00	2.49E-07
rs10774625	12	111910219	1.50E-10	8.83E-01	1.51E-11	8.88E-02	2.79E-02
rs4899539	14	75384162	4.42E-09	1.92E-01	1.82E-08	7.93E-01	1.46E-02
rs113629348	15	58545668	0	0.857	0	0.12	0.0228
rs2058914	15	63831984	7.02E-18	7.87E-01	1.84E-18	2.07E-01	6.05E-03
rs117254618	16	56892818	0	0.795	0	0.186	0.0184
rs72771478	16	57002118	0	0.807	0	0.175	0.0187
rs1109166	16	67977382	2.61E-69	4.24E-02	5.90E-68	9.57E-01	3.56E-04
rs78608249	17	7455707	8.28E-07	2.56E-01	1.77E-06	5.47E-01	1.96E-01
rs2518026	17	8052363	1.27E-06	7.93E-01	2.64E-07	1.65E-01	4.15E-02
rs241776	17	26644308	1.12E-21	5.30E-02	1.92E-20	9.12E-01	3.52E-02
rs2302774	17	38183090	8.26E-28	8.92E-01	9.48E-29	1.02E-01	5.74E-03
rs231517	17	41961789	8.85E-133	9.05E-01	7.59E-134	7.77E-02	1.69E-02
**rs17561950**	17	76392144	1.13E-42	8.25E-02	2.52E-42	1.84E-01	7.34E-01
rs4464148	18	46459032	2.46E-192	8.62E-01	2.83E-193	9.91E-02	3.85E-02
rs7237346	18	47058417	0	0.866	0	0.106	0.0279
rs144025261	19	41756563	4.00E-05	6.52E-01	3.86E-06	6.26E-02	2.85E-01
rs7412	19	45412079	1.78E-304	8.93E-01	1.69E-305	8.49E-02	2.23E-02
rs28850399	19	52314227	3.00E-08	8.74E-01	4.17E-09	1.21E-01	5.14E-03

rs6059958	20	30143278	8.60E-06	7.95E-01	9.53E-07	8.80E-02	1.17E-01
rs7268701	20	32901960	7.40E-08	9.36E-01	4.05E-09	5.12E-02	1.28E-02
rs2232170	22	31537122	0.00312	0.186	0.00922	0.55	0.252

Bold, SNPs have high probability to be a common causal genetic variant of a correlated trait pair. PP.H0.abf, PP.H1.abf, PP.H2.abf, PP.H3.abf, Coloc H4 Prob: the posterior probability of each possible configuration, referring to ABF analysis. H0: neither trait has a genetic association in the region. H1: Only trait 1 has a genetic association in the region. H2: only trait 2 has a genetic association in the region. H3: both traits are associated, but with different causal variants. H4: both traits are associated and share a single causal variant. ALS: Amyotrophic lateral sclerosis; ApoA1: apolipoprotein A1; BP: base pair; CHR: chromosome; HDL-C: high-density lipoprotein cholesterol; SNP: single nucleotide polymorphisms.
